# Watson‐Crick Base Pairing of *N*‐Methoxy‐1,3‐Oxazinane (MOANA) Nucleoside Analogues within Double‐Helical DNA

**DOI:** 10.1002/open.202300085

**Published:** 2023-07-04

**Authors:** Mark N. K. Afari, Kasper Nurmi, Pasi Virta, Tuomas Lönnberg

**Affiliations:** ^1^ Department of Chemistry University of Turku Henrikinkatu 2 20500 Turku Finland

**Keywords:** base pairing, DNA-templated ligation, dynamic base filling, oligonucleotides, oxazinanes

## Abstract

Hairpin oligodeoxynucleotides incorporating a (2*R*,3*S*)‐4‐(methoxyamino)butane‐1,2,3‐triol residue in the middle of the double‐helical stem and opposite to either one of the canonical nucleobases or an abasic 2‐(hydroxymethyl)tetrahydrofuran‐3‐ol spacer were synthesized. Under mildly acidic conditions, aromatic aldehydes reacted reversibly with these oligonucleotides, converting the (2*R*,3*S*)‐4‐(methoxyamino)butane‐1,2,3‐triol unit into a 2‐aryl‐*N*‐methoxy‐1,3‐oxazinane nucleoside analogue. The equilibrium of this reaction was found to be dependent on both the aldehyde and the nucleobase opposite to the modified residue. 9‐Formyl‐9‐deazaadenine, combining a large stacking surface with an array of hydrogen bond donors and acceptors, showed the highest affinity as well as selectivity consistent with the rules of Watson‐Crick base pairing. 5‐Formyluracil or indole‐3‐carbaldehyde, lacking in either stacking or hydrogen bonding ability, were incorporated with a much lower affinity and selectivity.

## Graphical abstract

Base filling within double‐helical oligonucleotides through formation of *N*‐methoxy‐1,3‐oxazinane nucleoside analogues was demonstrated. The most stable conjugates were obtained with 9‐deazaadenine as the base moiety of the modified residue and the stability constants depended on the opposite canonical nucleobase in a way consistent with Watson‐Crick base pairing. Respective conjugates with uracil or indole as the base moiety were less stable.

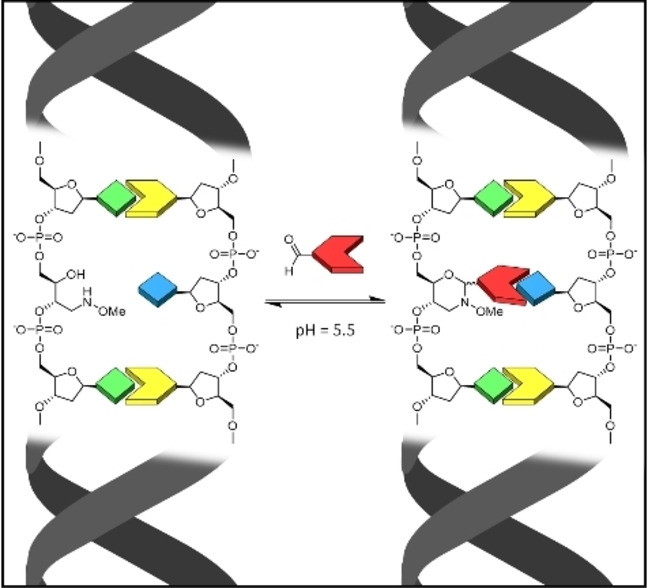

## Introduction

Incorporation of non‐canonical or completely artificial nucleobases into oligonucleotides can be motivated by the desire to stabilize double helices,[^1^, ^2^, ^3^, ^4^, ^5^, ^6^, ^7^, ^8^, ^9^, ^10^, ^11^, ^12^, ^13^, ^14^, ^15^, ^16^, ^17^] to expand the genetic alphabet,[^18^, ^19^, ^20^, ^21^, ^22^, ^23^] to confer resistance to nucleases,^[24]^ to enhance translation,^[25]^ to introduce metal ion binding sites[^26^, ^27^, ^28^, ^29^, ^30^, ^31^] or to gain insight into the factors governing the folding and function of nucleic acids.^[32]^ The established methods for introduction of modified nucleosides, namely automated solid‐phase synthesis and enzymatic polymerization, both involve labor‐intensive preparation of the respective building blocks (nucleoside 3′‐phosphoramidites or 5′‐triphosphates, respectively). The convertible nucleoside approach[^33^, ^34^] represents an important improvement, allowing post‐synthetic derivatization of selected residues with various functional groups. Typically, however, the base pairing properties of these derivatives remain unaltered along with the nucleobase core. Post‐synthetic introduction of truly diverse nucleobase analogues to a predetermined site within an oligonucleotide scaffold would involve efficient formation of the *N*‐glycosidic bond or a close mimic of it in aqueous solution – a challenging feat.

**Scheme 1:**
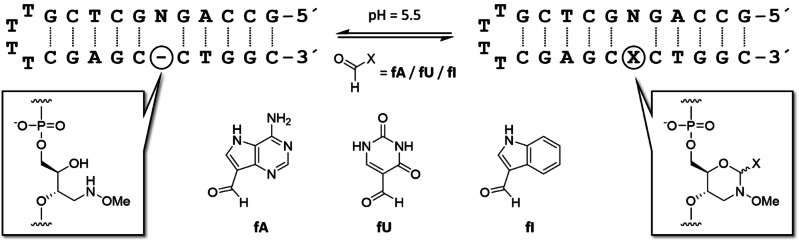
Incorporation of nucleobase analogues into hairpin oligonucleotides through *N*‐methoxy‐1,3‐oxazinane formation. “N” denotes any canonical nucleobase or an abasic 2‐(hydroxymethyl)tetrahydrofuran‐3‐ol spacer.

We have recently demonstrated post‐synthetic introduction of diverse nucleobase surrogates as aldehydes through condensation with a (2*R*,3*S*)‐4‐(methoxyamino)butane‐1,2,3 triol unit.^[35]^ In the resulting *N*‐methoxy‐1,3‐oxazinane (MOANA) nucleotide analogue, the aldehyde plays the role of the nucleobase and the oxazinane ring that of the ribose sugar. The oxazinane formation is readily reversible under conditions where Watson‐Crick base pairing rules apply, potentially allowing templated sequence specific incorporation of nucleobase analogues. Such a “base filling” approach, based on imine formation, has been described previously on a PNA[^36^, ^37^] and, with somewhat lower selectivity, on an 2‐aminobutane‐1,3‐diol‐modified DNA scaffold.^[38]^ The modest selectivity in the latter case was attributed to flexibility of the backbone. The MOANA backbone, while allowing equilibration of α and β pseudoanomers, is otherwise more rigid and could, hence, be expected to provide more stringent selectivity. In the present paper we report affinities of two nucleobase analogues bearing an aldehyde handle, namely 9‐formyl‐9‐deazaadenine (**fA**) and 5‐formyluracil (**fU**), for hairpin oligonucleotides placing each of the canonical nucleobases or an abasic site opposite to the (2*R*,3*S*)‐4‐(methoxyamino)butane‐1,2,3 triol scaffold (Scheme 1). For reference, indole‐3‐carbaldehyde (**fI**), featuring a purine‐sized ring system but no hydrogen bond donors or acceptors on the “Watson‐Crick face”, was also studied. **fA** and **fU** were chosen instead of the previously employed formylmethyl nucleobases for increased rigidity and a more natural‐like structure of the resulting MOANA nucleotide analogue.

## Results and Discussion

### Synthesis of 9‐formyl‐9‐deazaadenine

**Scheme 2:**
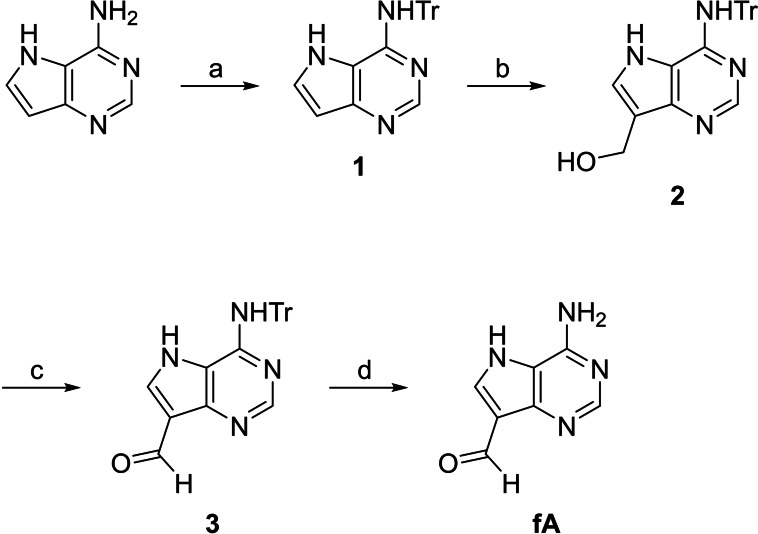
Synthesis of 9‐formyl‐9‐deazaadenine (fA). Reagents and conditions: a) TrCl, pyridine, 70 °C, 18 h; b) formaldehyde, K_2_CO_3_, 1,4‐dioxane, H_2_O, 50 °C, 16 h; c) Dess—Martin periodinane, CH_2_Cl_2_, THF, 25 °C, 16 h; d) TFA, CH_2_Cl_2_, 25 °C, 16 h.

For an adenine analogue that could be introduced to appropriately modified oligonucleotides through *N*‐methoxy‐1,3‐oxazinane formation, 9‐formyl‐9‐deazaadenine (**fA**) was synthesized as outlined in Scheme 2. The exocyclic amino function of 9‐deaazaadenine was first tritylated, affording intermediate **1**. The C9 position was then hydroxymethylated by treatment with aqueous formaldehyde under alkaline conditions. The resulting alcohol **2** was oxidized to the corresponding aldehyde **3** under Dess‐Martin conditions. Finally, the trityl protection was removed by treatment with trifluoroacetic acid, affording the desired product **fA**.

### Oligonucleotide synthesis

Five hairpin‐forming oligodeoxynucleotides were synthesized by an automated synthesizer, each incorporating the previously reported^[39]^ MOANA building block at position 6 and a variable residue at position 21 (Table 1). On completion of chain assembly, the oligonucleotides were deprotected and released from the solid support by conventional ammonolysis. The crude products were purified by RP‐HPLC, characterized by ESI‐TOF‐MS and quantified by UV/vis spectrophotometry. As reported previously, the 4‐hydroxybenzaldehyde protection of the MOANA building block was removed during ammonolysis and chromatographic purification, leaving the naked oligonucleotide scaffolds ready for subsequent experiments.

**Table 1:** Sequences and of the oligonucleotides used in this study. The variable residues paired with each other on formation of a hairpin are underlined.

Oligonucleotide	Sequence^[a]^
**ON1a**	5′‐GCCAGAGCTCGTTTTCGAGCXCTGGC‐3′
**ON1c**	5′‐GCCAGCGCTCGTTTTCGAGCXCTGGC‐3′
**ON1g**	5′‐GCCAGGGCTCGTTTTCGAGCXCTGGC‐3′
**ON1t**	5′‐GCCAGTGCTCGTTTTCGAGCXCTGGC‐3′
**ON1s**	5′‐GCCAGSGCTCGTTTTCGAGCXCTGGC‐3′

[a] “X” denotes the MOANA residue and “S” the abasic 2‐(hydroxymethyl)tetrahydrofuran‐3‐ol spacer.

### Duplex stability of the hairpin oligonucleotides under acidic conditions

The formation and hydrolysis of *N*‐methoxy‐1,3‐oxazinanes is acid‐catalyzed and impractically slow at neutral pH.^[39]^ On the other hand, excessively acidic conditions would prevent hybridization of the oligonucleotide scaffolds into the desired hairpin structures. To ensure stability of the hairpins under conditions where incorporation and dissociation of the nucleobase analogues would be reasonably fast, their UV melting profiles were measured at pH 5.5 (100 mM triethylammonium acetate buffer). All hairpins showed a typical monophasic sigmoidal melting curve (Figures S18–S22 in the Supporting Information), with the melting temperatures ranging from 54 to 57 °C. At ambient temperature (23 °C), the oligonucleotides were estimated to exist almost entirely in the hairpin form. Therefore, pH=5.5 and *T*=23 °C were deemed as an acceptable tradeoff between duplex stability and reaction rate and all subsequent experiments were carried out under these conditions.

### Kinetics of oxazinane formation and dissociation

To establish sufficiently long equilibration times for the affinity measurements, rates of dissociation of aldehydes **fA**, **fU** and **fI** from the hairpin oligonucleotides were determined. The aldehyde and the oligonucleotide were first incubated for several days at pH 5.5 and 23 °C and at a concentration where a significant fraction of the aldehyde was expected to be incorporated into the oligonucleotide through N‐methoxy‐1,3‐oxazinane formation (500 μM). The reaction mixtures were then diluted to a concentration where nearly quantitative dissociation was expected (1.0 μM) and their composition monitored by RP‐HPLC at appropriate time intervals. All oligonucleotide components eluted as a single broad peak so quantification was based on the relative peak area of the free aldehyde (illustrative examples of chromatograms presented in Figures S23–S37 in the Supporting Information). Formation of the expected conjugates could, however, be verified by UPLC‐MS (chromatograms and spectra presented in Figures S38–S67 in the Supporting Information). It is worth pointing out that in each case incorporation of only a single aldehyde molecule was observed, ruling out non‐specific imine formation with exocyclic amino groups of the nucleobases.

The time‐concentration profiles (Figures S68–S70 in the Supporting Information) were in reasonable agreement with the first‐order rate law, given the small size of the aldehyde peak. First‐order rate constants for the dissociation reactions were obtained by non‐linear least‐squares fitting of Equation 1 to the experimental data.

(1) $${\left[aldehyde\right]}_{t}=\;\left({\left[aldehyde\right]}_{eq}-{\left[aldehyde\right]}_{0}\right)\left(1-e^{-kt}\right)+{\left[aldehyde\right]}_{0}$$

[aldehyde]_0_, [aldehyde]_t_ and [aldehyde]_eq_ are concentrations of the free aldehyde at the beginning of the reaction, at time point t and at equilibrium, respectively, and *k* is the observed first‐order rate constant. The results are summarized in Table 2. The dissociation rate mainly depended on the aldehyde, with **fA** being released somewhat faster than **fU** or **fI**. Based on the observed rate constants, appropriate equilibration times were deemed to be 48 h for **fA** and 120 h for **fU** and **fI**. While the dissociation rates observed may appear impractically high, we would like to point out that adjusting the pH to physiological (7.40) would cause a rate retardation of nearly two orders of magnitude, effectively “freezing” the equilibrium. Furthermore, we have shown previously^[39]^ that the rate can be controlled in a predictable way by introduction of electron‐withdrawing or ‐donating substituents on the arene moiety of the aldehyde.

**Table 2:** Observed first‐order rate constants for the release of aldehydes **fA**, **fU** and **fI** from hairpin oligonucleotides **ON1a**, **ON1c**, **ON1g**, **ON1t** and **ON1s**; *T*=23 °C; pH=5.5 (100 mM triethylammonium acetate buffer).

Oligonucleotide	*k*(**fA**)/10^−5^ s^−1^	*k*(**fU**)/10^−5^ s^−1^	*k*(**fI**)/10^−5^ s^−1^
**ON1a**	3.4±0.8	2.5±0.6	2±1
**ON1c**	9±1	1.4±0.1	1.3±0.5
**ON1g**	4.6±0.2	0.4±0.3	2±1
**ON1t**	4±2	1.5±0.5	0.6±0.4
**ON1s**	7±4	1.1±0.6	4.0±0.9

### Affinity of the aldehydes for the hairpin oligonucleotides

**Figure 1:**
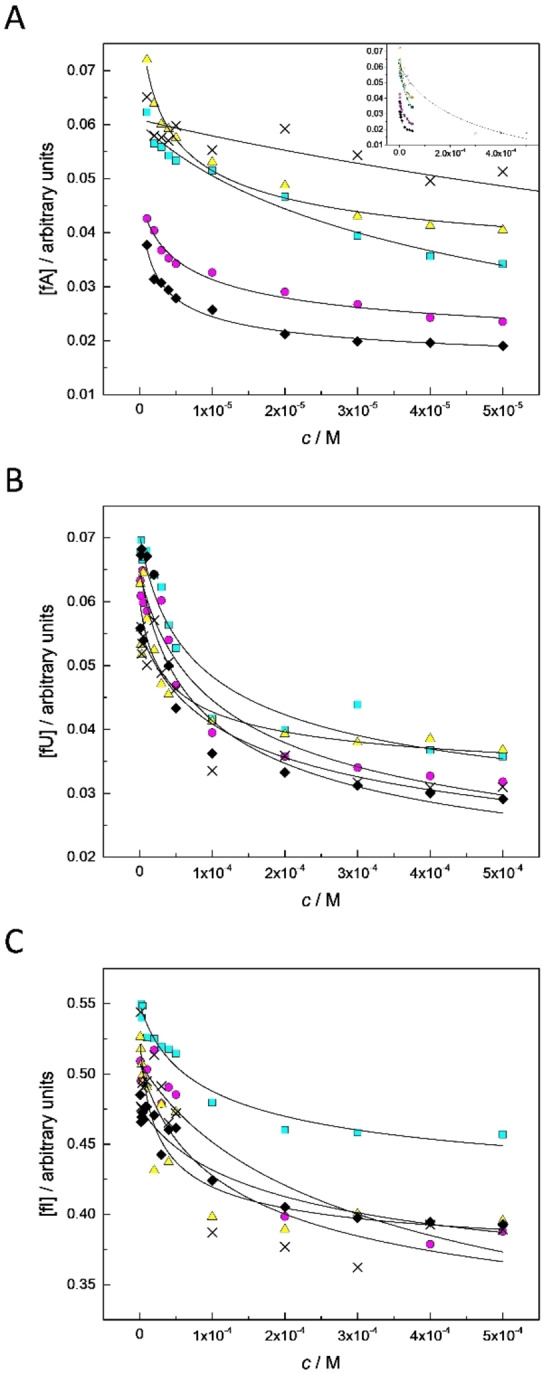
Relative peak areas of free **fA** (A), **fU** (B) and **fI** (C) as a function of concentration of equimolar mixtures of the aldehyde and the hairpin oligonucleotide **ON1a** (cyan squares), **ON1c** (magenta circles), **ON1 g** (yellow triangles), **ON1 t** (black diamonds) and **ON1 s** (crosses); *T*=23 °C; pH=5.5 (100 mM triethylammonium acetate buffer). In panel A, an inset has been added to show the leveling off with all aldehyde‐oligonucleotide combinations.

For determination of the stability constants of the covalent conjugates of aldehydes **fA**, **fU** and **fI** and hairpin oligonucleotides **ON1a**, **ON1c**, **ON1g**, **ON1t** and **ON1s**, a series of equimolar (1–500 μM) mixtures of each aldehyde and oligonucleotide were first prepared and incubated for an appropriate time period (48 h for **fA** and 120 h for **fU** and **fI**) at pH 5.5 and 23 °C. The mixtures were then analyzed by RP‐HPLC as described above. As expected, the relative peak area of the free aldehyde decreased with increasing total concentration of the aldehyde and the oligonucleotide and leveled off at higher concentrations (Figure 1). As the total aldehyde and oligonucleotide concentrations of all mixtures were equimolar, decrease in the concentration of the free aldehyde translates to increase in the concentration of the aldehyde incorporated to the hairpin oligonucleotide. The stability constants were determined by non‐linear least‐squares fitting of Equation 2 to the experimental data.

(2) $$A{\left(aldehyde\right)}_{c}=A{\left(aldehyde\right)}_{0}+\left(\begin{array}{c} A{\left(aldehyde\right)}_{\infty }-\\ A{\left(aldehyde\right)}_{0} \end{array}\right)\left(1+\frac{1-\sqrt{4Kc+1}}{2Kc}\right)$$

*A*(aldehyde)_o_, *A*(aldehyde)_c_ and *A*(aldehyde)_∞_ are relative peak areas of the free aldehyde at zero, *c* and infinite concentration of the aldehyde and the oligonucleotide, respectively. By definition, concentration of the free aldehyde is equal to total aldehyde concentration at *c*=0 (*i. e*., infinite dilution) and zero at c→∞. In practice, more reliable results were obtained when these parameters were also fitted. *K* is the stability constant of the oligonucleotide‐aldehyde conjugate and *c* the total concentration of the oligonucleotide and the aldehyde. The stability constants obtained are summarized in Table 3.

**Table 3:** Stability constants for the covalent conjugates of aldehydes **fA**, **fU** and **fI** with hairpin oligonucleotides **ON1a**, **ON1c**, **ON1g**, **ON1t** and **ON1s**; *T*=23 °C; pH=5.5 (100 mM triethylammonium acetate buffer).

Oligonucleotide	*K*(**fA**)/10^3^ M^−1^	*K*(**fU**)/10^3^ M^−1^	*K*(**fI**)/10^3^ M^−1^
**ON1a**	20±10	13±6	12±5
**ON1c**	200±100	9±5	3±2
**ON1g**	300±200	30±30	50±40
**ON1t**	700±400	12±10	5±3
**ON1s**	3±2	8±5	8±8

With hairpin oligonucleotides **ON1c**, **ON1g** and **ON1t**, the adenine analogue **fA** exhibited 1–2 orders of magnitude higher affinity than **fU** or **fI**. The affinity for **ON1a** and **ON1s** was much lower and comparable to that of **fU** and **fI**. Gratifyingly, the highest stability constant was observed when **fA** was incorporated opposite to thymine, consistent with formation of a Watson‐Crick base pair. The relatively high affinity for **ON1c** and **ON1g** suggests hydrogen bonding also with cytosine and guanine. The apparent stability of the latter putative base pair was unexpected but could be understood if the base moiety of the MOANA residue adopted the *syn* conformation, thereby alleviating the steric crowding of a purine‐purine mispair. The importance of hydrogen bonding interactions is further underlined by the very low affinity for **ON1s**, with an abasic site opposite to **fA**. Finally, affinity for **ON1a** was intermediate between those for **ON1s** and **ON1c**, possibly indicating formation of a weak mispair.

Nearly all of the conjugates formed by the uracil analogue **fU** were less stable than those formed by **fA**. The sole exception was hairpin **ON1s**, in which case a similar low stability constant was obtained. Discrimination between canonical nucleobases opposite to the MOANA residue was also weaker and the preferred partner was guanine, rather than the Watson‐Crick partner adenine. Uracil‐guanine wobble base pairs can be quite strong[^40^, ^41^, ^42^] and in the present case might be more compatible with the modified backbone than the canonical base pair with adenine. In any case, this putative wobble base pair was still much weaker than those between **fA** and cytosine, guanine or thymine.

One possible explanation for the much higher affinity of **fA** compared to **fU** could be its larger stacking surface. To test this hypothesis, **fI** was used as a control featuring a similar stacking surface as **fA** but no hydrogen bond donors or acceptors on its “Watson‐Crick face”. With the exception of hairpin **ON1 s**, the stability constants were in all cases lower than those of **fA** and in most cases also lower than those of **fU**. Clearly, incorporation of aldehydes into the hairpin oligonucleotide scaffolds is not driven only by base stacking but hydrogen bonding also plays an important role.

In summary, stability constants of the **fA** conjugates were high and the observed dependence on the opposite nucleobase suggests Watson‐Crick base pairing. Stability constants of the **fU** and **fI** conjugates were much lower and in both cases purines were favored over pyrimidines as the opposite nucleobase. Perhaps the more strongly stacking purine bases are better able to preorganize a hydrophobic binding pocket for the aldehyde and in the case of **fU** and, especially, **fI**, such preorganization is more important than a correct pattern of hydrogen bond donors and acceptors.

### Duplex stability of hairpin‐fA conjugates

With **fA**, the stability constants obtained were sufficiently high that a significant fraction of **fA** should remain incorporated into the hairpin oligonucleotide even under conditions of a typical UV melting experiment (*i. e*., low micromolar concentration). Encouraged by this result, we determined the melting temperatures of all hairpin‐**fA** conjugates to explore the impact of a single MOANA residue on the stability of a DNA double helix. 500 μM mixtures of **fA** and either **ON1a**, **ON1c**, **ON1g**, **ON1t** or **ON1 s** were first prepared and preincubated as described above, after which they were diluted to 1.0 μM concentration by addition of 20 mM cacodylate buffer (pH=7.4), the ionic strength of which had been adjusted to 0.10 M with sodium perchlorate. For reference, an otherwise identical set of samples but lacking **fA** was also prepared. At pH 7.4, dissociation of the conjugates should be approximately two orders of magnitude slower than at pH 5.5, translating to half‐lives between 9 and 200 days at room temperature. At the high end of the temperature ramp (90 °C), however, the reaction would be much faster, possibly proceeding to a significant extent during the UV melting experiment.

All hairpin‐**fA** conjugates showed monophasic sigmoidal melting profiles (Figures S71–S75 in the Supporting Information). The curves were reproducible over at least three heating and cooling cycles, contrary to what might be expected if the conjugates dissociated during measurement. On the other hand, monophasicity of the curves is in apparent conflict with the coexistence of two oligonucleotide populations – one with and one without **fA**. The highest melting temperature (66.7±0.1 °C) was obtained with **ON1t‐fA**, *i. e*. the conjugate that also showed the highest stability constant. **ON1c‐fA** and **ON1g‐fA** also reasonably followed this pattern, with melting temperatures of 63.9±0.1 and 65.0±0.1 °C, respectively. The melting temperatures of **ON1a‐fA** and **ON1s‐fA** (66.2±0.1 and 64.4±0.4 °C, respectively), however, were comparable to those of **ON1c‐fA**, **ON1g‐fA** and **ON1t‐fA** despite their much lower stability constants. Comparison with the corresponding data in the absence of **fA** (Figures S76–S80 in the Supporting Information) eliminated these discrepancies to some extent – a substantial increase in melting temperature (+3.7 °C) on addition of **fA** was only observed with **ON1 t**. Nevertheless, at least with only a single MOANA residue in the middle of a DNA double helix, correlation between duplex and base pair stability appears rather tenuous. All melting temperatures obtained are summarized in Table 4.

**Table 4:** Melting temperatures of the hairpin oligonucleotides **ON1a**, **ON1c**, **ON1g**, **ON1t** and **ON1s** in the absence and presence of the aldehyde **fA**; [oligonucleotides]=1.0 μM; [**fA**]=0/1.0 μM; pH=7.40 (20 mM cacodylate buffer); *I*(NaClO_4_)=0.10 M.

Oligonucleotide	*T*_m_ (no aldehyde)/°C	*T*_m_ (**fA**)/°C	*ΔT*_m_/°C
**ON1a**	65.7±0.1	66.2±0.1	+0.5
**ON1c**	63.3±0.1	63.9±0.1	+0.6
**ON1g**	66.3±0.1	65.0±0.1	−1.3
**ON1t**	63.0±0.1	66.7±0.1	+3.7
**ON1s**	64.1±0.1	64.4±0.4	+0.3

## Conclusions

Aldehyde derivatives of 9‐deazaadenine and uracil were readily incorporated in the middle of the double‐helical stem of hairpin oligonucleotides through formation of an *N*‐methoxy‐1,3‐oxazinane with a 4‐methoxyamino‐1,2,3‐butanetriol residue. With the adenine analogue, stability of the conjugates thus obtained followed the rules of Watson‐Crick base pairing, with dissociation constants in the micromolar range. Respective conjugates with the uracil analogue or indole‐3‐carbaldehyde were much less stable, underlining the importance of both hydrogen bonding and base stacking for efficient incorporation of an aldehyde. Correlation between affinity of an aldehyde for a hairpin oligonucleotide and duplex stability of the resulting conjugate could not be established unambiguously.

## Experimental

**General methods**: Unless otherwise stated, all chemicals were commercial products that were used as received. Solvents were dried over activated 4 Å molecular sieves. HPLC elution buffers were prepared using freshly distilled Et_3_N. NMR spectra were recorded on a Bruker Biospin 500 MHz NMR spectrometer, the chemical shifts (δ, ppm) being referenced to residual solvent signals. Mass spectra were recorded on Bruker Daltonics micrOTOF−Q and Waters ACQUITY RDa mass spectrometers.

***N*** ^**6**^ **‐Trityl‐9‐deazaadenine (1)**: 9‐Deazaadenine (1.00 g, 7.47 mmol) was co‐evaporated from anhydrous pyridine (2×7 mL). The residue was dissolved in anhydrous pyridine (15 mL) and TrCl (8.32 g, 29.8 mmol) was added. The reaction mixture was stirred at 70 °C for 18 h, after which it was evaporated to dryness. The residue was dissolved in CH_2_Cl_2_ (50 mL) and washed with saturated aqueous NaHCO_3_ (100 mL). The aqueous phase was back extracted with CH_2_Cl_2_ (4×100 mL) and the combined organic phases dried over Na_2_SO_4_ and evaporated to dryness. The residue was purified by silica gel column chromatography eluting with a mixture of MeOH and CH_2_Cl_2_ (stepwise gradient, 10 : 90–30 : 70, *v*/*v*), affording 0.973 g (34 %) of the desired product **1**. ^1^H NMR (500 MHz, DMSO‐*d*_6_) δ 11.68 (s, 1H, H7), 8.04 (s, 1H, N^6^H), 7.82 (s, 1H, H2), 7.57 (t, *J*=2.9 Hz, 1H, H8), 7.35 (m, 6H, Ph−H3 & H5), 7.28 (m, 6H, Ph−H2 & H6), 7.20 (m, 3H, Ph−H4), 6.37 (dd, *J*=1.9, 3.0 Hz, 1H, H9). ^13^C NMR (126 MHz, DMSO‐*d*_6_) δ 149.0 (C6), 148.7 (C4), 146.2 (C2), 145.5 (Ph−C1), 129.4 (Ph−C3 & C5), 128.5 (C8), 128.0 (Ph−C2&C6), 126.8 (pH−C4), 115.1 (C5), 101.3 (C9), 71.0 (CPh_3_). HRMS (ESI^+^‐TOF): *m*/*z* calcd. for [C_25_H_21_N_4_]: 377.1761; found: 377.1765 [M+H]^+^.

***N*** ^**6**^ **‐Trityl‐9‐(hydroxymethyl)‐9‐deazaadenine (2)**: *N* ^6^‐Trityl‐9‐deazaadenine (**2**, 0.973 g, 2.59 mmol) was dissolved in a mixture of 1,4‐dioxane (45 mL), H_2_O (11.5 mL) and 37 % aqueous formaldehyde (17 mL). K_2_CO_3_ (0.871 g, 6.30 mmol) was added and the reaction mixture stirred at 50 °C for 16 h, after which it was evaporated to dryness. The residue was suspended in CH_2_Cl_2_ (50 mL) and washed with saturated aqueous NaHCO_3_ (100 mL). The aqueous phase was back extracted with CH_2_Cl_2_ (50 mL) and the combined organic phases dried over Na_2_SO_4_ and evaporated to dryness. The residue was purified by silica gel column chromatography eluting with a mixture of MeOH and CH_2_Cl_2_ (10 : 90, *v*/*v*), affording 0.458 g (43 %) of the desired product **2**. ^1^H NMR (500 MHz, DMSO‐*d*_6_) δ 11.35 (s, 1H, H7), 7.81 (s, 1H, N^6^H), 7.76 (s, 1H, H2), 7.44 (d, *J*=2.8 Hz, 1H, H8), 7.33 (m, 6H, Ph−H3 & H5), 7.28 (m, 6H, Ph−H2 & H6), 7.19 (m, 3H, Ph−H4), 4.70 (t, *J*=5.3 Hz, 1H, OH), 4.56 (d, *J*=5.3 Hz, 2H, CH_2_). ^13^C NMR (126 MHz, DMSO‐*d*_6_) δ 148.9 (C2 & C4), 148.7 (C6), 145.7 (Ph−C1), 129.3 (Ph−C3 & C5), 127.9 (Ph−C2&C6), 126.7 (pH−C4), 126.4 (C8), 116.8 (C9), 115.3 (C5), 70.9 (CPh_3_), 54.3 (CH_2_). HRMS (ESI^+^‐TOF): *m*/*z* calcd. for [C_26_H_23_N_4_O]: 407.1866; found: 407.1873 [M+H]^+^.

**9‐Formyl‐9‐deazaadenine (fA)**: *N* ^6^‐Trityl‐9‐(hydroxymethyl)‐9‐deazaadenine (**3**, 0.231 g, 0.568 mmol) was dissolved in THF (31 mL). A solution of Dess‐Martin periodinane in CH_2_Cl_2_ (8–12 %, 4.7 mL) was added and the resulting mixture stirred at 25 °C for 16 h, after which it was evaporated to dryness. The residue was suspended in CH_2_Cl_2_ (50 mL) and washed with saturated aqueous NaHCO_3_ (150 mL). The aqueous phase was back extracted with CH_2_Cl_2_ (50 mL) and the combined organic phases washed with saturated aqueous Na_2_S_2_O_3_ and NaHCO_3_ (150 mL), dried over Na_2_SO_4_ and evaporated to dryness. The residue was passed through a silica gel column eluting with a mixture of Et_3_N, MeOH and CH_2_Cl_2_ (1 : 4 : 95, *v*/*v*). Intermediate **3** thus obtained (38.5 mg, 95.2 μmol) was dissolved in CH_2_Cl_2_ (2.0 mL). TFA (100 μL) was added and the resulting mixture stirred at 25 °C for 16 h. Et_3_N (380 μL) was added and the mixture evaporated to dryness. The residue was purified by RP‐HPLC on a Hypersil ODS C18 column (250×10 mm, 5 μm) eluting with 10 % MeCN in 50 mM aqueous triethylammonium acetate. The flow rate was 3.0 mL min^−1^ and the detection wavelength 260 nm. The main fraction, eluting at 6.2 min, contained the desired product **fA** but also triethylammonium trifluoroacetate as a major impurity. As the planned experiments involving **fA** and oligonucleotides would be carried out in relatively concentrated triethylammonium acetate buffers, this contamination was deemed acceptable. The yield of **fA** was determined as 2.24 μmol (2.3 %) by ^1^H NMR using DMF as an internal standard. ^1^H NMR (500 MHz, DMSO‐*d*_6_) δ 10.03 (s, 1H, CHO), 9.16 (br, 2H, NH_2_), 8.60 (s, 1H, H2), 8.56 (s, 1H, H8). ^13^C NMR (126 MHz, DMSO‐*d*_6_) δ 185.5 (CHO), 172.4 (C6), 153.4 (C2), 147.8 (C4), 138.4 (C8), 114.6 (C5), 114.0 (C9). HRMS (ESI^+^‐TOF): *m*/*z* calcd. for [C_7_H_7_N_4_O]: 163.0614; found: 163.0615 [M+H]^+^.

**Oligonucleotide synthesis**: The hairpin oligonucleotide scaffolds **ON1a**, **ON1c**, **ON1g**, **ON1t** and **ON1s** (Table 1) were synthesized by an ÄKTA oligopilot plus 10 DNA/RNA synthesizer in 2 μmol scale on CPG support following conventional phosphoramidite strategy. The previously reported^[39]^ 4‐(benzoyloxy)benzylidene‐protected building block was used for introduction of the (2*R*,3*S*)‐4‐(methoxyamino)butane‐1,2,3‐triol residue. Recycling time recommended for commercially available DNA building blocks (120 s) was employed throughout the syntheses, with 5‐(benzylthio)‐1H‐tetrazole as the activator. Trityl response monitoring revealed essentially quantitative yields for all couplings. After chain assembly, the oligonucleotides were released from the solid support and the phosphate and base protections and the benzoyl protection of the modified building block removed by incubation in 25 % aqueous NH_3_ at 55 °C for 18 h. The crude products were purified by RP‐HPLC on a Thermo Scientific Hypersil ODS C18 column (250×10 mm, 5 μm) eluting with a gradient (5–20 % over 25 min) of MeCN in 50 mM aqueous triethylammonium acetate buffer (pH=7.0), the flow rate being 3.0 mL min^−1^ and the detection wavelength 260 nm. The purified oligonucleotides were characterized by UPLC‐ESI‐TOF‐MS and quantified by UV spectrophotometry using molar absorptivities calculated by an implementation of the nearest‐neighbors method.

**Kinetic and equilibrium studies**: For both kinetic and equilibrium studies, a 500 μM stock solution of a hairpin oligonucleotide (**ON1a**, **ON1c**, **ON1g**, **ON1t** or **ON1 s**) and an aldehyde (**fA**, **fU** or **fI**) was first prepared as follows. Appropriate volumes of stock solutions of the oligonucleotide in H_2_O and the aldehyde in either DMSO (for **fA** and **fI**) or H_2_O (for **fU**) were mixed and lyophilized. The residues were dissolved in a 200 mM aqueous triethylammonium acetate buffer (pH=5.5) and the resulting solutions incubated at 23 °C for either 48 (in the case of **fA**) or 120 h (in the case of **fU** and **fI**). In each case, formation of the expected conjugates was verified by UPLC‐MS (chromatograms and spectra presented in Figures S38–S67 in the Supporting Information).

The rates of dissociation of the hairpin‐aldehyde conjugates were determined by diluting the aforementioned 500 μM stock solutions to 1.0 μM with 200 mM aqueous triethylammonium acetate buffer (pH=5.5) and analyzing the composition of the solutions thus obtained at appropriate time intervals by RP‐HPLC on a Thermo Scientific Hypersil ODS C18 column (250×4.6 mm, 5 μm) eluting with a linear gradient of of MeCN in 50 mM aqueous triethylammonium acetate buffer (pH=7.0), the flow rate being 1.0 mL min^−1^. With **fA** and **fU**, the MeCN content was increased from 5 to 29 % over 15 min. With the more hydrophobic **fI**, this gradient was followed by a slightly steeper one, from 29 to 40 % over 5 min and, finally, 5 min of isocratic elution at 40 %. The detection wavelength was 260 nm for **fA** and **fU** and 300 nm for **fI**. Retention times for **fA**, **fU**, **fI** and the oligonucleotides were 6.9, 4.2, 18.5 and 8.5 min, respectively. Peak areas of the aldehydes were normalized against the sum of all peak areas and plotted as a function of time. Dissociation rate constants were obtained by non‐linear least squares fitting of the first‐order rate law (Eq. 1) to these plots.

For determination of the stability constants of the hairpin‐aldehyde conjugates, the aforementioned 500 μM stock solutions were first diluted with 200 mM aqueous triethylammonium acetate buffer (pH=5.5) to various concentrations spanning a range of 1–500 μM. These solutions were then allowed to equilibrate for either 48 (in the case of **fA**) or 120 h (in the case of **fU** and **fI**), after which their composition was analyzed by RP‐HPLC as described above. To avoid detector overflow, the more concentrated samples were diluted to 1 μM with 200 mM aqueous Et_3_N before analysis. The normalized aldehyde peak areas were plotted as a function of concentration of the original reaction solutions and the stability constants obtained by non‐linear least‐squares fitting of Equation 2 to these plots.

**UV melting temperature measurements**: Samples of the naked hairpin oligonucleotides were prepared by dilution of the appropriate stock solutions to 1.0 μM concentration with 100 mM triethylammonium acetate buffer (pH=5.5). Samples of the hairpin‐aldehyde conjugates were prepared by dilution of the aforementioned 500 μM stock solutions to 1.0 μM concentration with 20 mM cacodylate buffer (pH=7.4), the ionic strength of which was adjusted to 0.10 M with NaClO_4_. The samples were placed in quartz cuvettes with optical path length of 10 mm. Melting profiles were acquired by a PerkinElmer Lambda 35 UV/vis spectrophotometer equipped with a Peltier temperature control unit. Three heating and cooling ramps with a slope of 0.5 °C min^−1^ were carried out between 10 and 90 °C and absorbance at 260 nm was recorded at 0.5 °C intervals. Melting temperatures were obtained as the center points of Gaussian peaks fitted to the first derivative curves of the UV melting profiles.

## Conflict of interest

The authors declare no conflict of interest.

1

## Supporting information

As a service to our authors and readers, this journal provides supporting information supplied by the authors. Such materials are peer reviewed and may be re‐organized for online delivery, but are not copy‐edited or typeset. Technical support issues arising from supporting information (other than missing files) should be addressed to the authors.

Supporting Information

## Data Availability

The data that support the findings of this study are available in the supplementary material of this article.
