# Supplementary material for: Watson‐Crick Base Pairing of N‐Methoxy‐1,3‐Oxazinane (MOANA) Nucleoside Analogues within Double‐Helical DNA
Source: ChemistryOpen. 2023 Jul 4;12(7):e202300085. doi: 10.1002/open.202300085 (PMC10319751; doi:10.1002/open.202300085)
Supplement: Supplementary file 1 — Supporting Information [file OPEN-12-e202300085-s001.pdf]

ChemistryOpen

Supporting Information

Watson-Crick Base Pairing of *N*-Methoxy-1,3-Oxazinane (MOANA) Nucleoside Analogues within Double-Helical DNA

Mark N. K. Afari, Kasper Nurmi, Pasi Virta, and Tuomas Lönnberg*

Contents

Figure S1. ^{1}H NMR spectrum of compound 1.	S3
Figure S2. ^{13}C NMR spectrum of compound 1.	S4
Figure S3. ^{1}H NMR spectrum of compound 2.	S6
Figure S4. ^{13}C NMR spectrum of compound 2.	S8
Figure S5. ^{1}H NMR spectrum of compound fA.	S11
Figure S6. ^{13}C NMR spectrum of compound fA.	S12
Figure S7. HPLC traces of crude hairpin oligonucleotides ON1a, ON1c, ON1g, ON1t and ON1s.	S14
Figure S8. UV and extracted ion UPLC traces of purified hairpin oligonucleotide ON1a.	S15
Figure S9. Mass spectrum of hairpin oligonucleotide ON1a.	S16
Figure S10. UV and extracted ion UPLC traces of purified hairpin oligonucleotide ON1c.	S17
Figure S11. Mass spectrum of hairpin oligonucleotide ON1c.	S18
Figure S12. UV and extracted ion UPLC traces of purified hairpin oligonucleotide ON1g.	S19
Figure S13. Mass spectrum of hairpin oligonucleotide ON1g.	S20
Figure S14. UV and extracted ion UPLC traces of purified hairpin oligonucleotide ON1t.	S21
Figure S15. Mass spectrum of hairpin oligonucleotide ON1t.	S22
Figure S16. UV and extracted ion UPLC traces of purified hairpin oligonucleotide ON1s.	S23
Figure S17. Mass spectrum of hairpin oligonucleotide ON1s; pH = 5.5.	S24
Figure S18. UV melting curve and its first derivative of ON1a; pH = 5.5.	S25
Figure S19. UV melting curve and its first derivative of ON1c; pH = 5.5.	26
Figure S20. UV melting curve and its first derivative of ON1g; pH = 5.5.	S27
Figure S21. UV melting curve and its first derivative of ON1t; pH = 5.5.	S28
Figure S22. UV melting curve and its first derivative of ON1s; pH = 5.5.	S29
Figure S23. HPLC trace of a mixture of fA and ON1a.	S30
Figure S24. HPLC trace of a mixture of fA and ON1c.	S31
Figure S25. HPLC trace of a mixture of fA and ON1g.	S32
Figure S26. HPLC trace of a mixture of fA and ON1t.	S33
Figure S27. HPLC trace of a mixture of fA and ON1s.	S34
Figure S28. HPLC trace of a mixture of fU and ON1a.	S35
Figure S29. HPLC trace of a mixture of fU and ON1c.	S36
Figure S30. HPLC trace of a mixture of fU and ON1g.	S37
Figure S31. HPLC trace of a mixture of fU and ON1t.	S38
Figure S32. HPLC trace of a mixture of fU and ON1s.	S39
Figure S33. HPLC trace of a mixture of fI and ON1a.	S40
Figure S34. HPLC trace of a mixture of fI and ON1c.	S41
Figure S35. HPLC trace of a mixture of fI and ON1g.	S42
Figure S36. HPLC trace of a mixture of fI and ON1t.	S43
Figure S37. HPLC trace of a mixture of fI and ON1s.	S44
Figure S38. UV and extracted ion UPLC traces of a 50 µM mixture of hairpin oligonucleotide ON1a and fA.	S45
Figure S39. Mass spectrum of the covalent conjugate of hairpin oligonucleotide ON1a and fA.	S46
Figure S40. UV and extracted ion UPLC traces of a 50 µM mixture of hairpin oligonucleotide ON1c and fA.	S47
Figure S41. Mass spectrum of the covalent conjugate of hairpin oligonucleotide ON1c and fA.	S48
Figure S42. UV and extracted ion UPLC traces of a 50 µM mixture of hairpin oligonucleotide ON1g and fA.	S49
Figure S43. Mass spectrum of the covalent conjugate of hairpin oligonucleotide ON1g and fA.	S50
Figure S44. UV and extracted ion UPLC traces of a 50 µM mixture of hairpin oligonucleotide ON1t and fA.	S51
Figure S45. Mass spectrum of the covalent conjugate of hairpin oligonucleotide ON1t and fA.	S52

Contents (continued)

Figure S46. UV and extracted ion UPLC traces of a 50 µM mixture of hairpin oligonucleotide ON1s and fA. S53
Figure S47. Mass spectrum of the covalent conjugate of hairpin oligonucleotide ON1s and fA. S54
Figure S48. UV and extracted ion UPLC traces of a 50 µM mixture of hairpin oligonucleotide ON1a and fU. S55
Figure S49. Mass spectrum of the covalent conjugate of hairpin oligonucleotide ON1a and fU. S56
Figure S50. UV and extracted ion UPLC traces of a 50 µM mixture of hairpin oligonucleotide ON1c and fU. S57
Figure S51. Mass spectrum of the covalent conjugate of hairpin oligonucleotide ON1c and fU. S58
Figure S52. UV and extracted ion UPLC traces of a 50 µM mixture of hairpin oligonucleotide ON1g and fU. S59
Figure S53. Mass spectrum of the covalent conjugate of hairpin oligonucleotide ON1g and fU. S60
Figure S54. UV and extracted ion UPLC traces of a 50 µM mixture of hairpin oligonucleotide ON1t and fU. S61
Figure S55. Mass spectrum of the covalent conjugate of hairpin oligonucleotide ON1t and fU. S62
Figure S56. UV and extracted ion UPLC traces of a 50 µM mixture of hairpin oligonucleotide ON1s and fU. S63
Figure S57. Mass spectrum of the covalent conjugate of hairpin oligonucleotide ON1s and fU. S64
Figure S58. UV and extracted ion UPLC traces of a 50 µM mixture of hairpin oligonucleotide ON1a and fI. S65
Figure S59. Mass spectrum of the covalent conjugate of hairpin oligonucleotide ON1a and fI. S66
Figure S60. UV and extracted ion UPLC traces of a 50 µM mixture of hairpin oligonucleotide ON1c and fI. S67
Figure S61. Mass spectrum of the covalent conjugate of hairpin oligonucleotide ON1c and fI. S68
Figure S62. UV and extracted ion UPLC traces of a 50 µM mixture of hairpin oligonucleotide ON1g and fI. S69
Figure S63. Mass spectrum of the covalent conjugate of hairpin oligonucleotide ON1g and fI. S70
Figure S64. UV and extracted ion UPLC traces of a 50 µM mixture of hairpin oligonucleotide ON1t and fI. S71
Figure S65. Mass spectrum of the covalent conjugate of hairpin oligonucleotide ON1t and fI. S72
Figure S66. UV and extracted ion UPLC traces of a 50 µM mixture of hairpin oligonucleotide ON1s and fI. S73
Figure S67. Mass spectrum of the covalent conjugate of hairpin oligonucleotide ON1s and fI. S74
Figure S68. Relative peak area of free fA in a 1.0 µM equimolar mixture of fA and ON1a, ON1c, ON1g, ON1t and ON1s as a function of time. S75
Figure S69. Relative peak area of free fU in a 1.0 µM equimolar mixture of fU and ON1a, ON1c, ON1g, ON1t and ON1s as a function of time. S76
Figure S70. Relative peak area of free fI in a 1.0 µM equimolar mixture of fI and ON1a, ON1c, ON1g, ON1t and ON1s as a function of time. S77
Figure S71. UV melting curve and its first derivative of the covalent conjugate of ON1a and fA. S78
Figure S72. UV melting curve and its first derivative of the covalent conjugate of ON1c and fA. S79
Figure S73. UV melting curve and its first derivative of the covalent conjugate of ON1g and fA. S80
Figure S74. UV melting curve and its first derivative of the covalent conjugate of ON1t and fA. S81
Figure S75. UV melting curve and its first derivative of the covalent conjugate of ON1s and fA. S82
Figure S76. UV melting curve and its first derivative of ON1a; pH = 7.4. S83
Figure S77. UV melting curve and its first derivative of ON1c; pH = 7.4. S84
Figure S78. UV melting curve and its first derivative of ON1g; pH = 7.4. S85
Figure S79. UV melting curve and its first derivative of ON1t; pH = 7.4. S86
Figure S80. UV melting curve and its first derivative of ON1s; pH = 7.4. S87

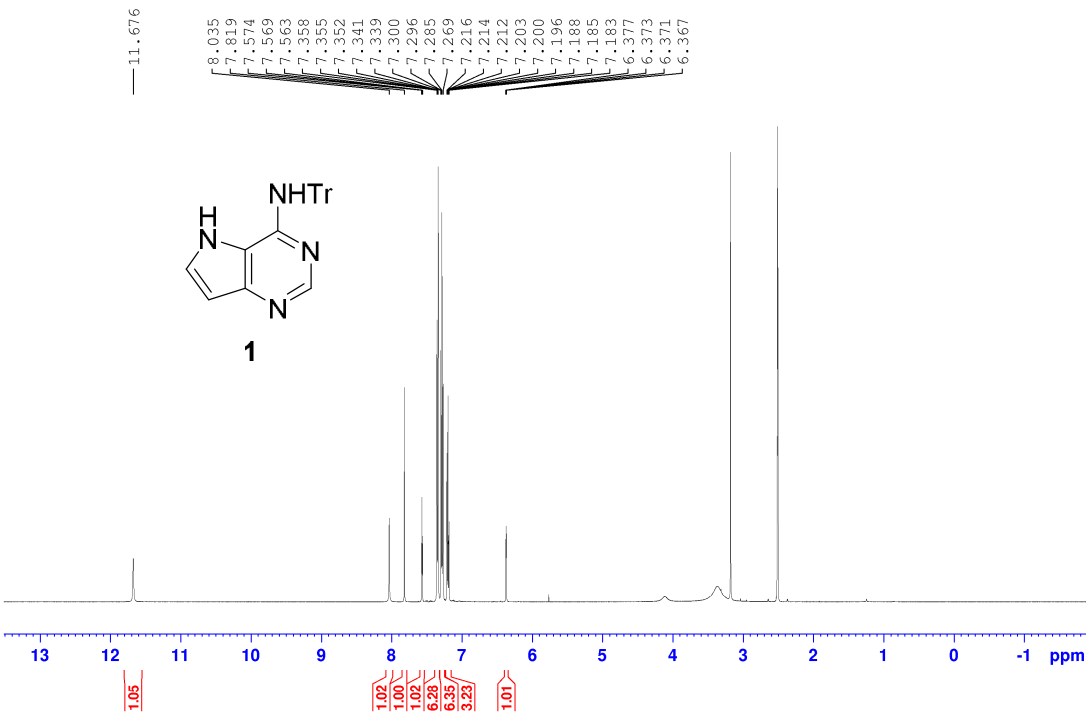

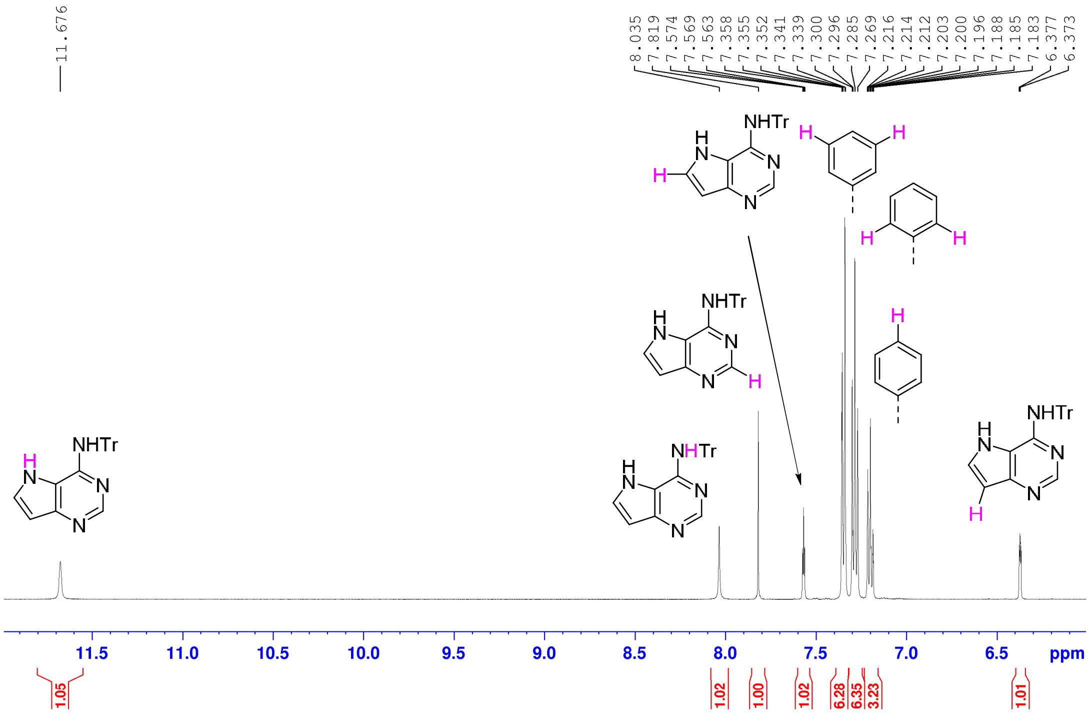

Figure S1. ^{1}H NMR spectrum of compound 1 (500 MHz, DMSO-d_6).

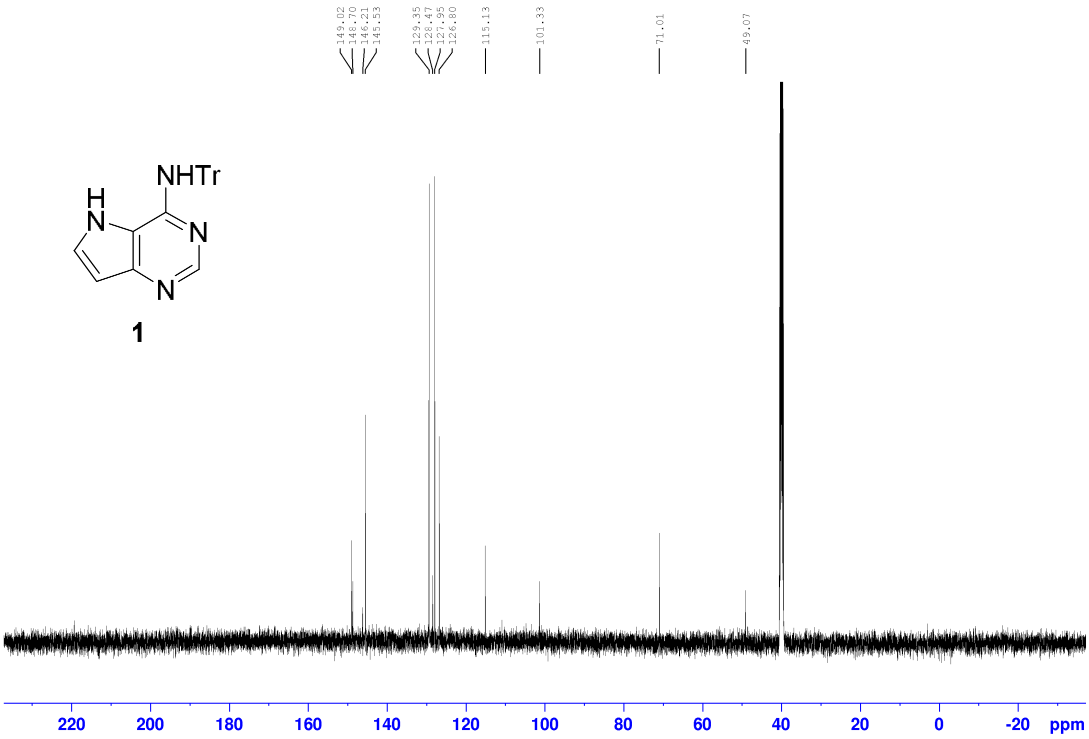

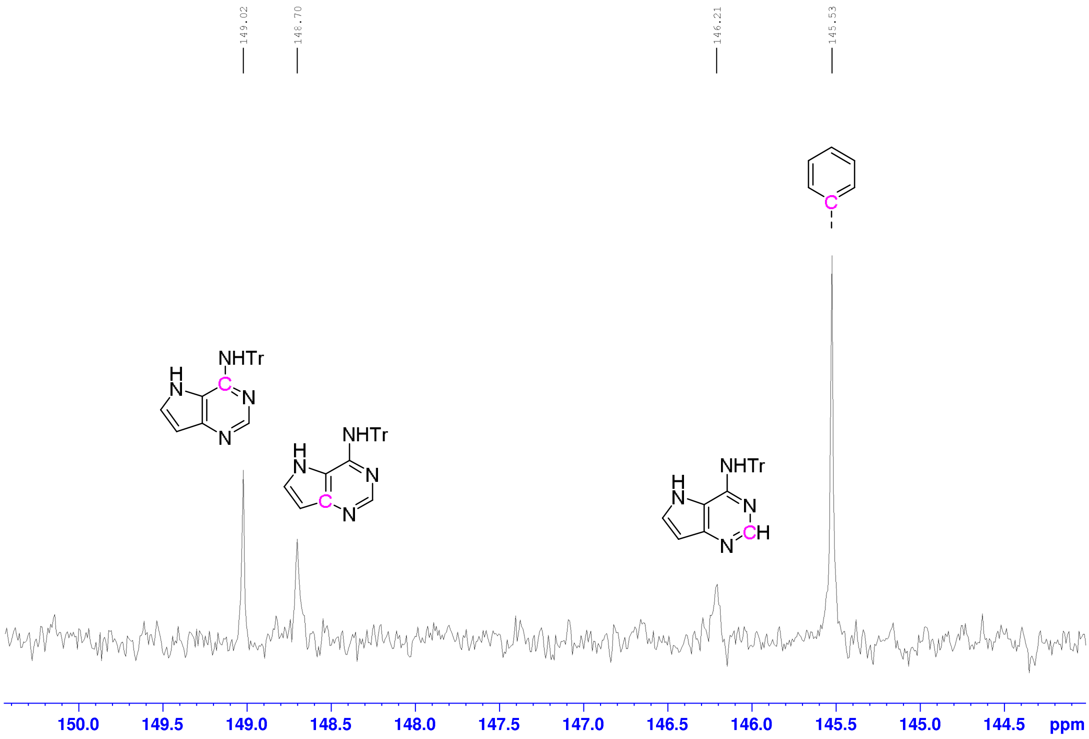

Figure S2. ^{13}C NMR spectrum of compound 1 (126 MHz, DMSO-d_6).

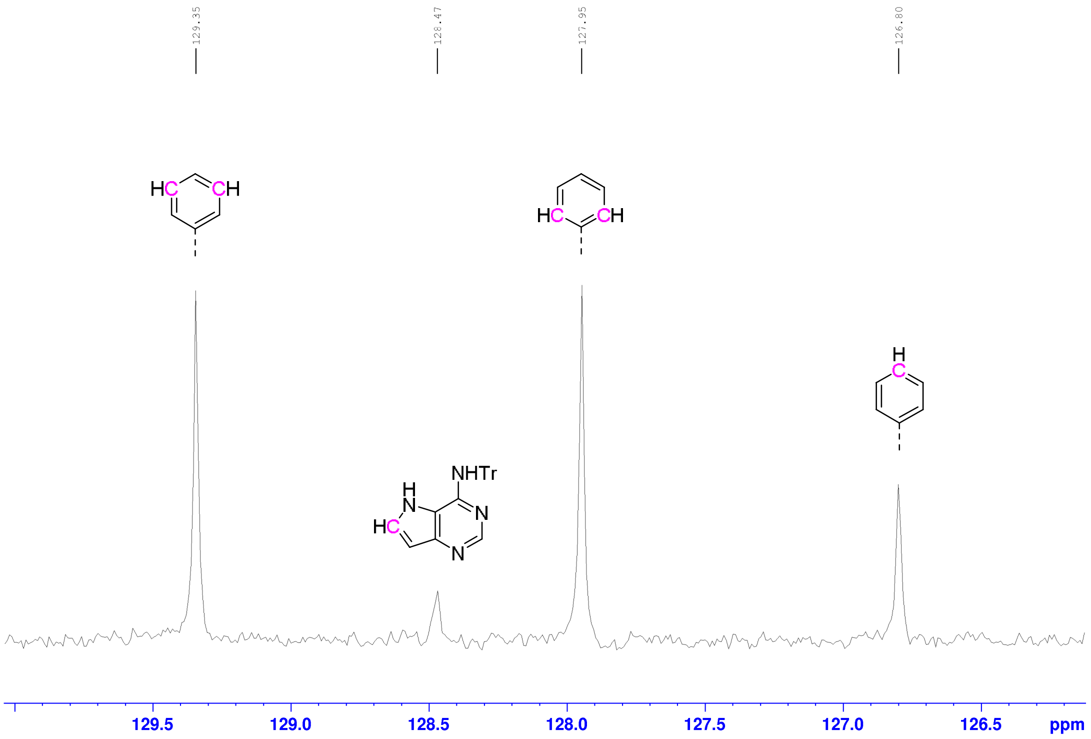

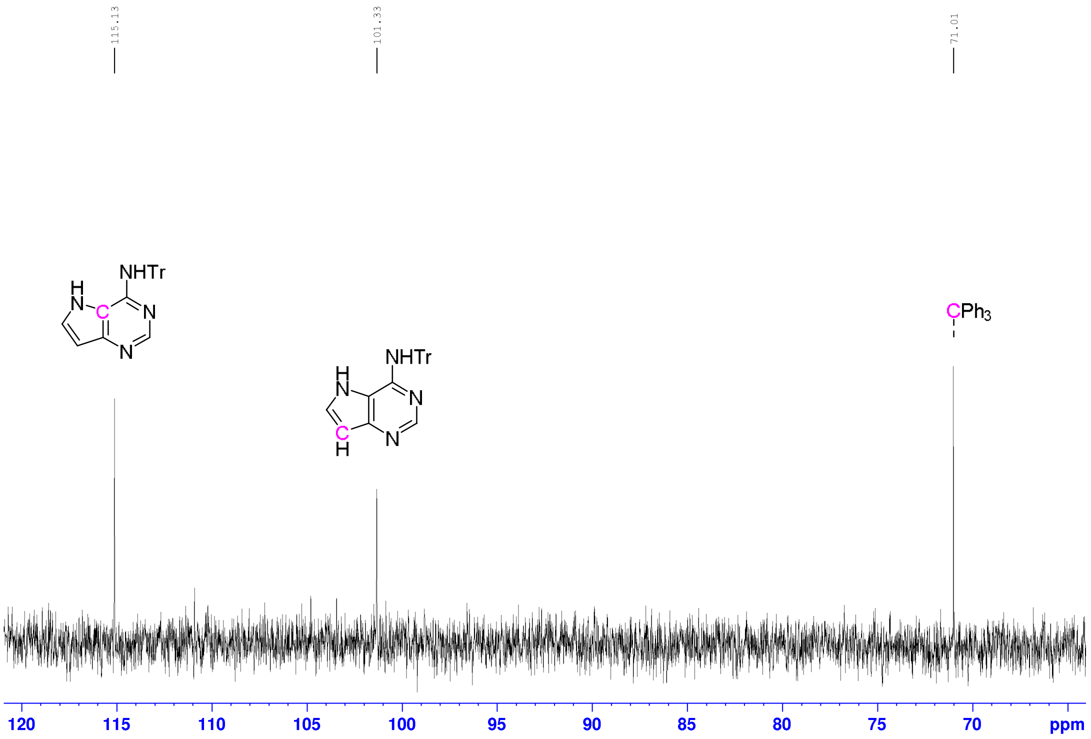

Figure S2 (continued). ^{13}C NMR spectrum of compound 1 (126 MHz, DMSO-d_6).

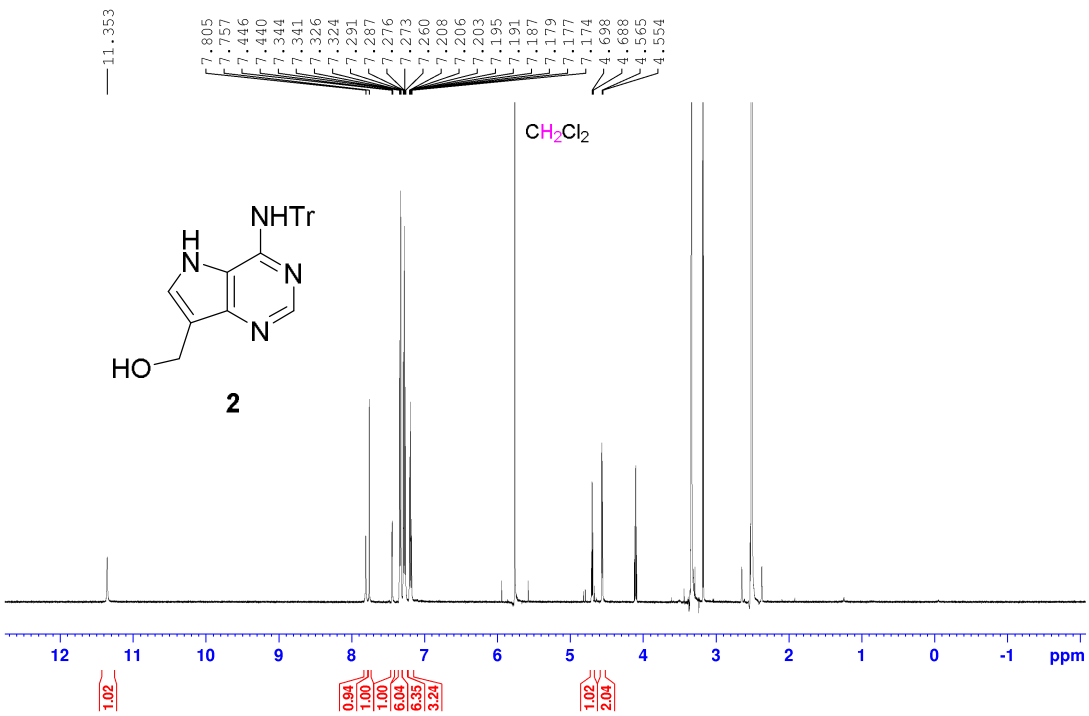

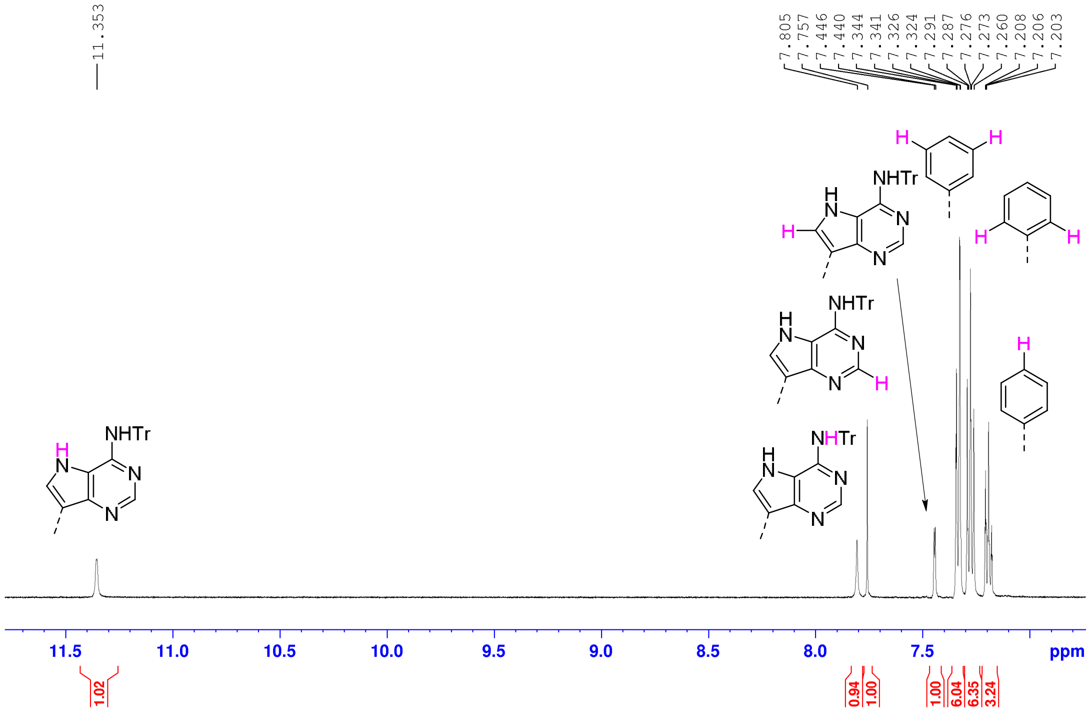

Figure S3. ^{1}H NMR spectrum of compound 2 (500 MHz, DMSO-d_6).

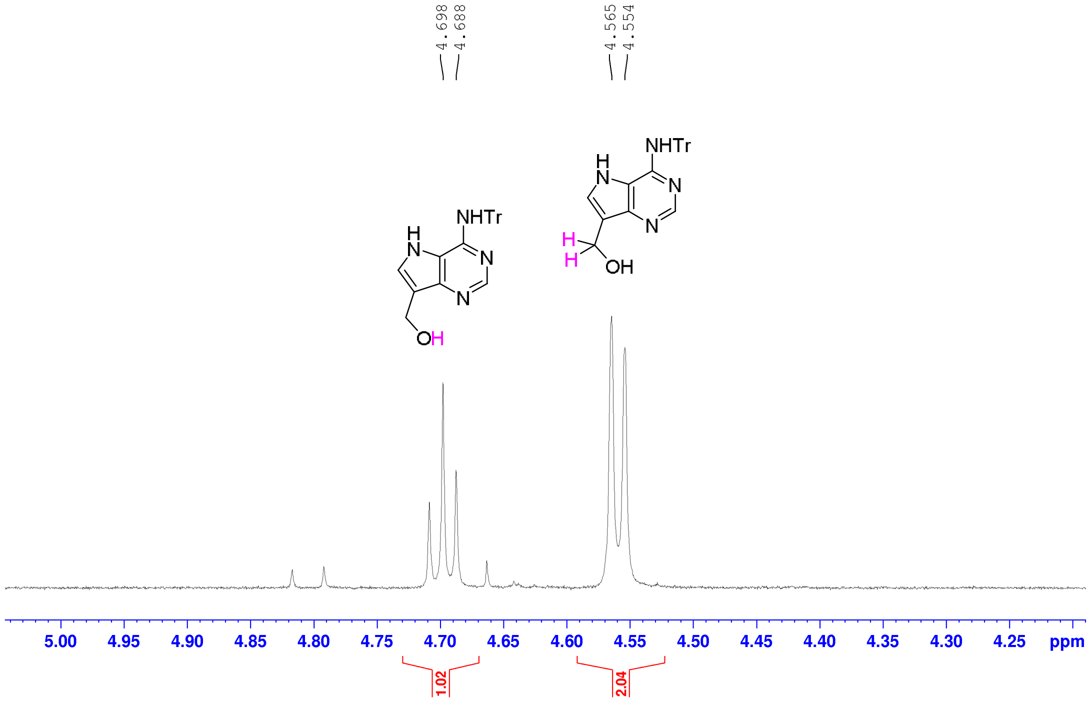

Figure S3 (continued). ^{1}H NMR spectrum of compound 2 (500 MHz, DMSO-d_6).

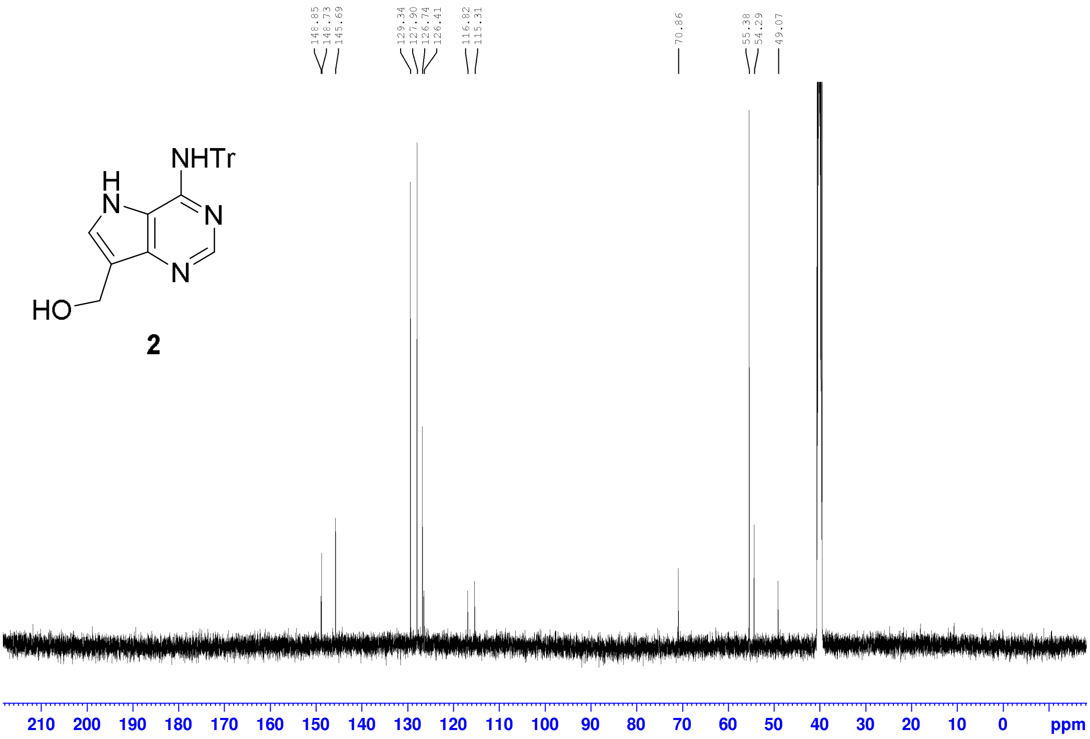

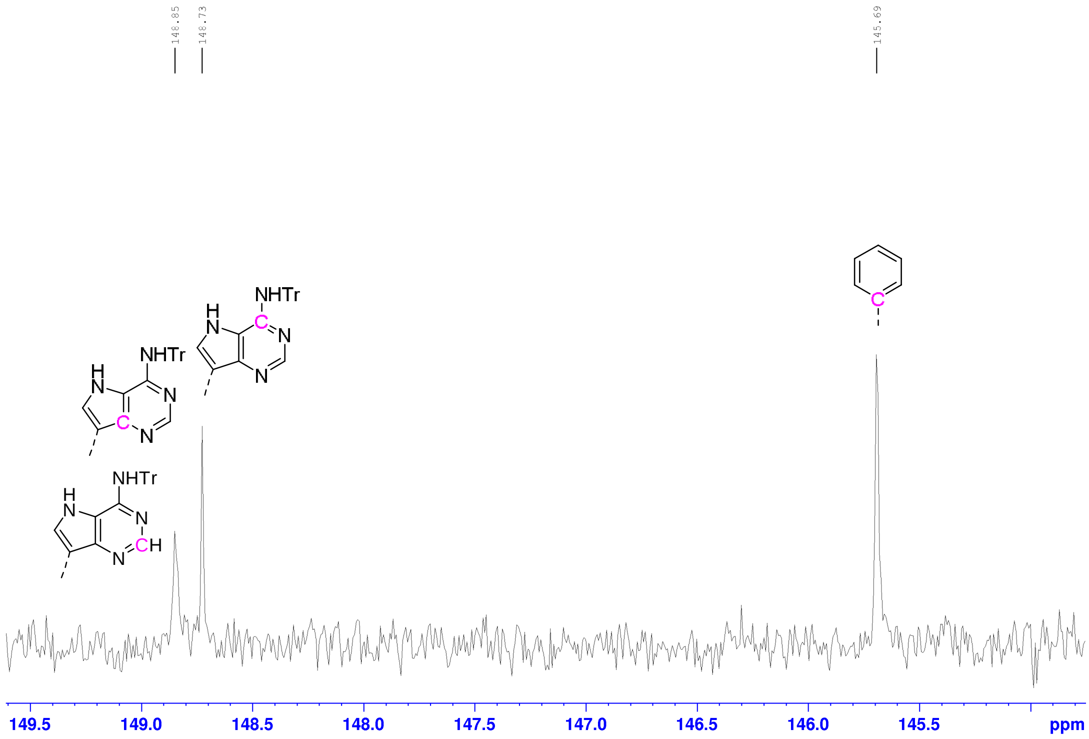

Figure S4. ^{13}C NMR spectrum of compound 2 (126 MHz, DMSO-d_6).

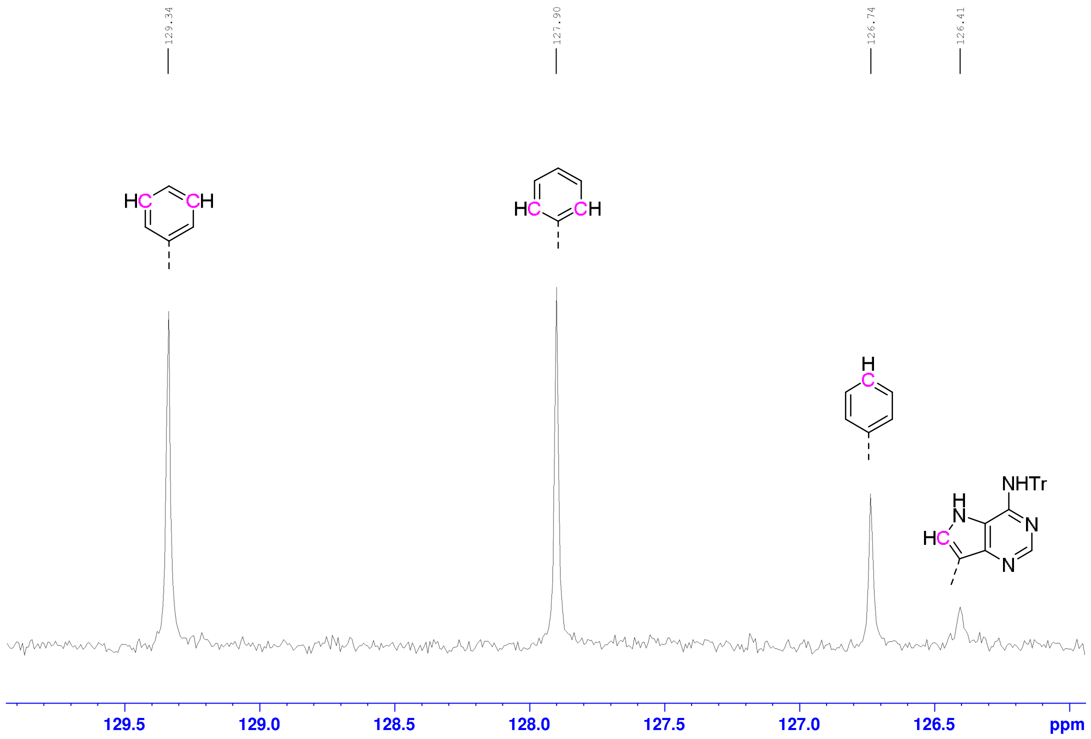

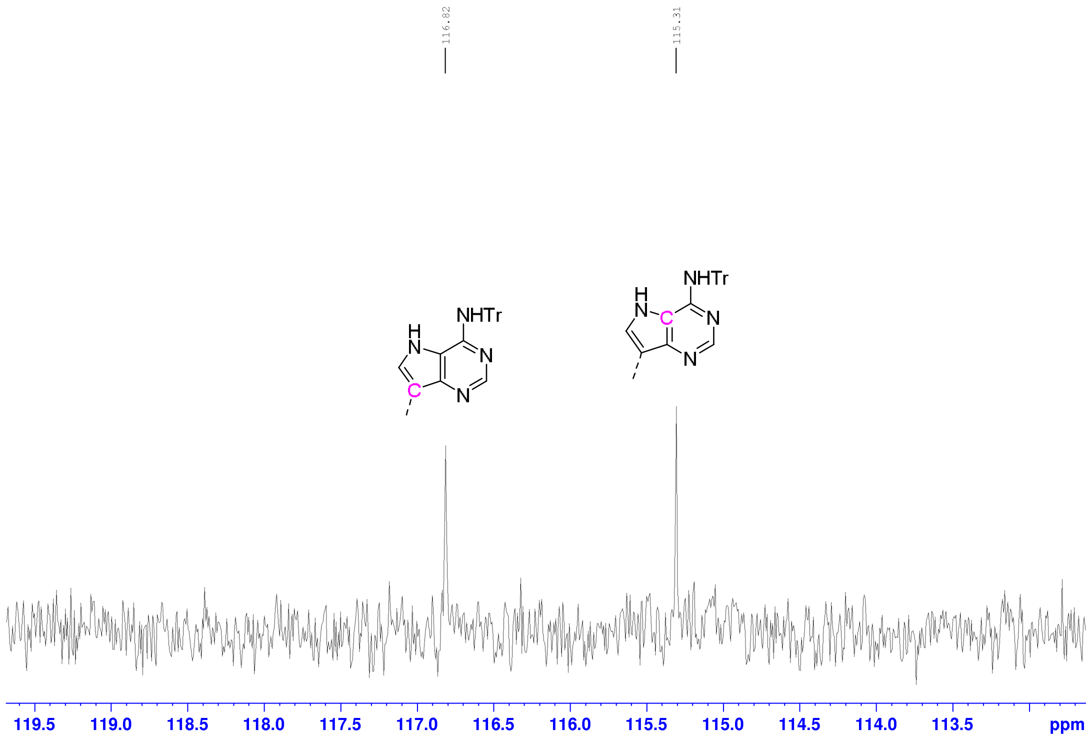

Figure S4 (continued). ^{13}C NMR spectrum of compound 2 (126 MHz, DMSO-d_6).

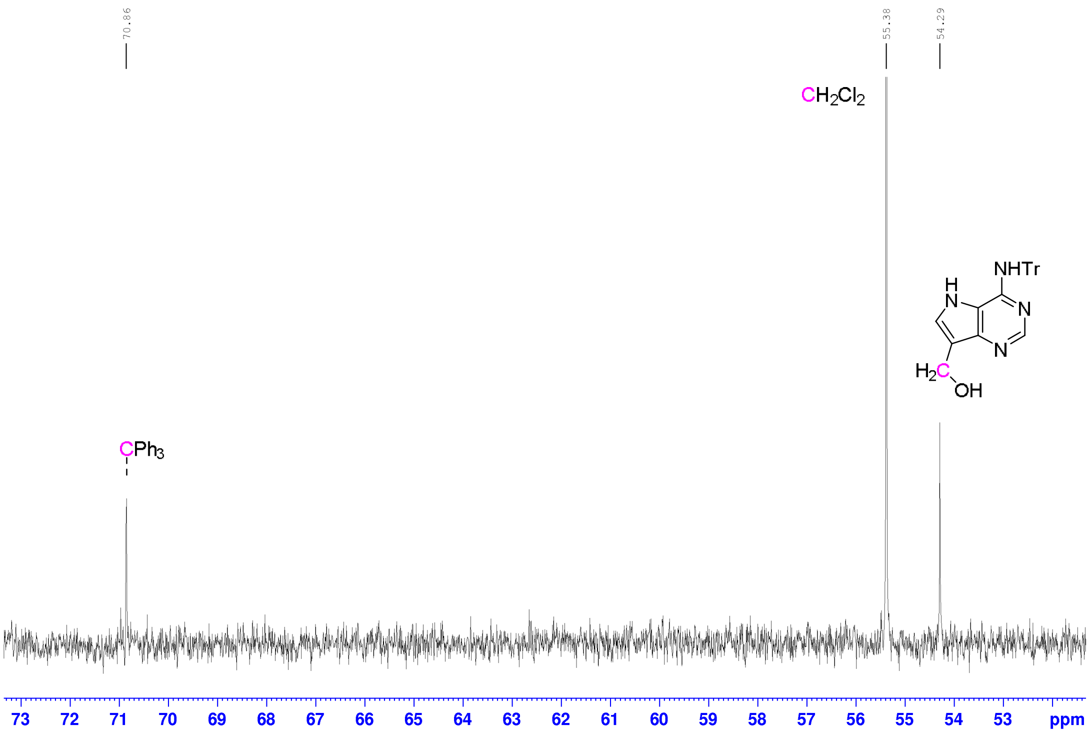

Figure S4 (continued). ^{13}C NMR spectrum of compound 2 (126 MHz, DMSO-d_6).

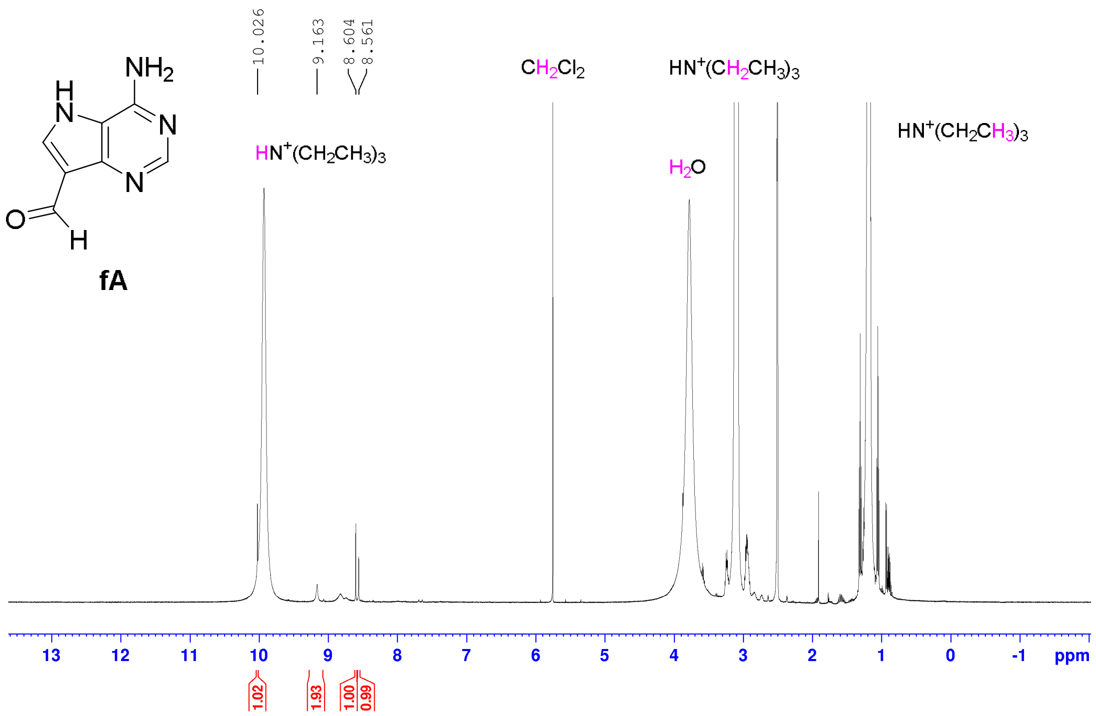

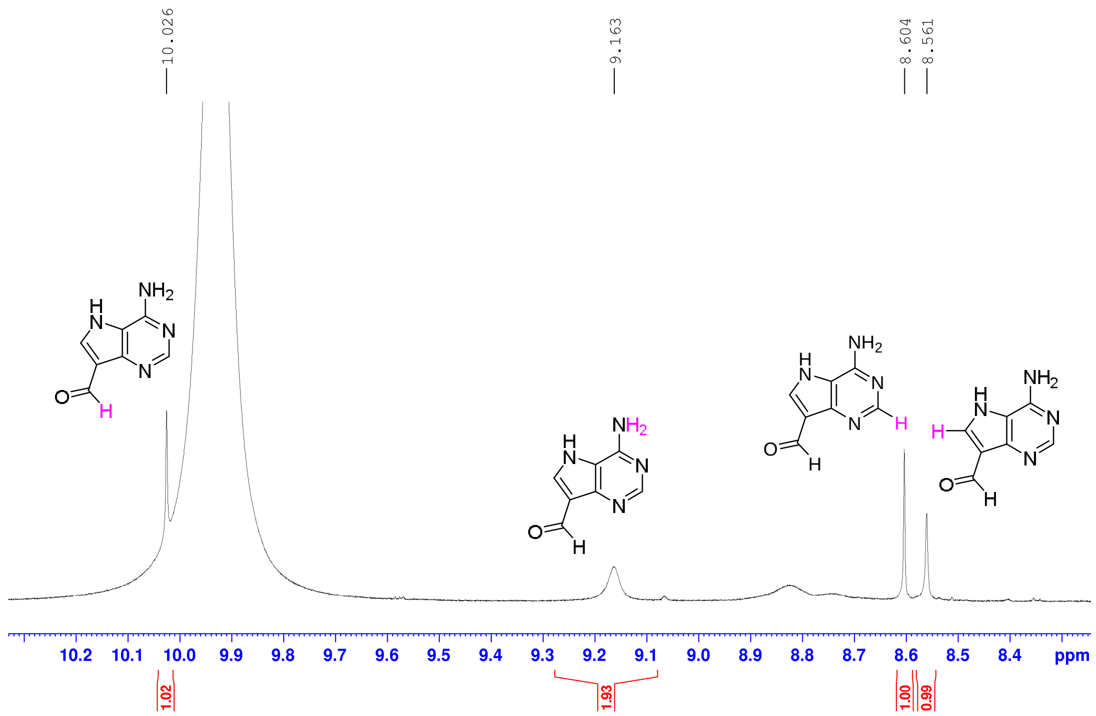

Figure S5. ^{1}H NMR spectrum of compound fA (500 MHz, DMSO-d_6).

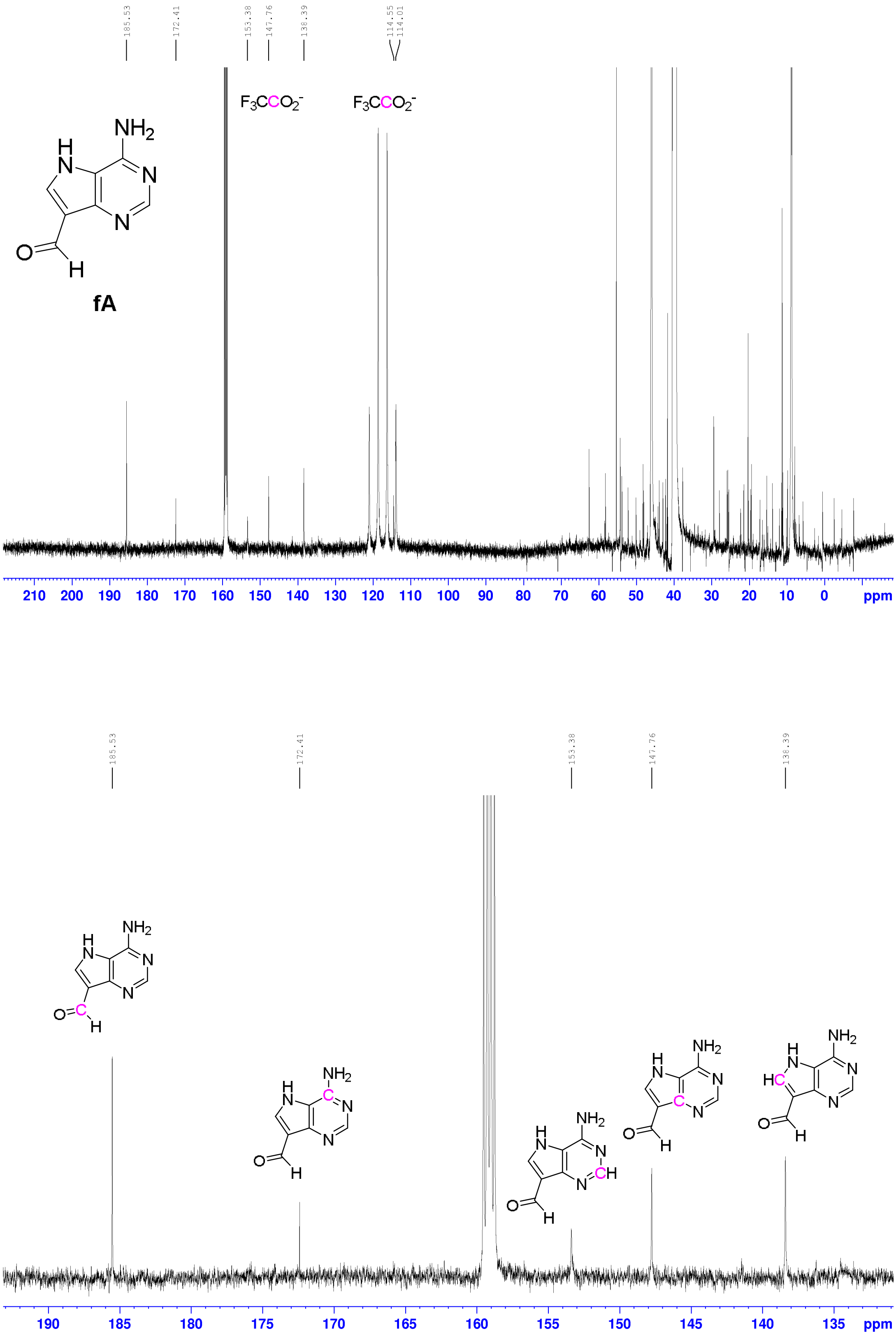

Figure S6. ^{13}C NMR spectrum of compound fA (126 MHz, DMSO-d_6).

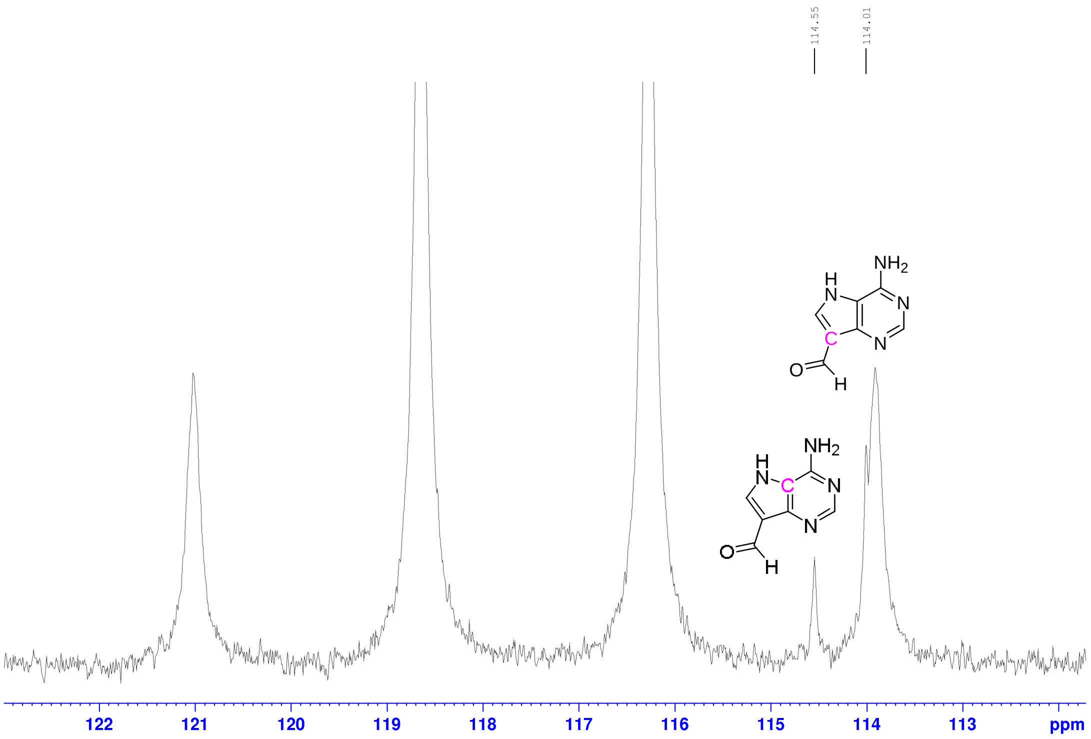

Figure S6 (continued). ^{13}C NMR spectrum of compound 2 (126 MHz, DMSO-d_6).

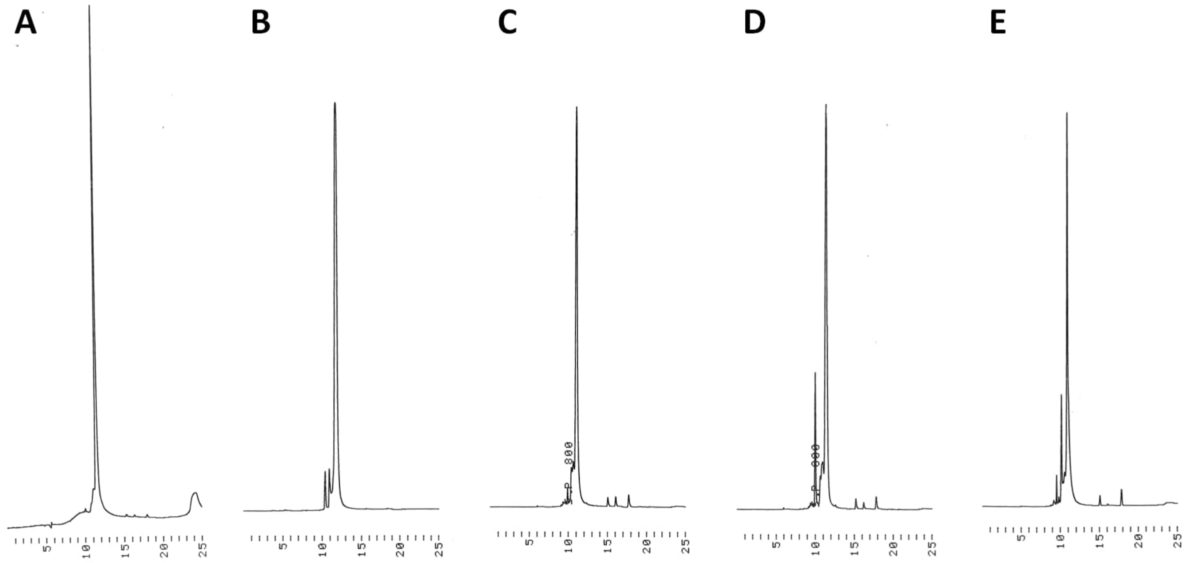

Figure S7. HPLC traces of crude hairpin oligonucleotides ON1a (A), ON1c (B), ON1g (C), ON1t (D) and ON1s (E); Thermo Scientific ODS Hypersil column (250×10 mm, 5 µm); flow rate = 3.0 mL min^{-1}; linear gradient (5—20% over 25 min) of MeCN in 50 mM aqueous triethylammonium acetate buffer (pH = 7.0); λ = 260 nm.

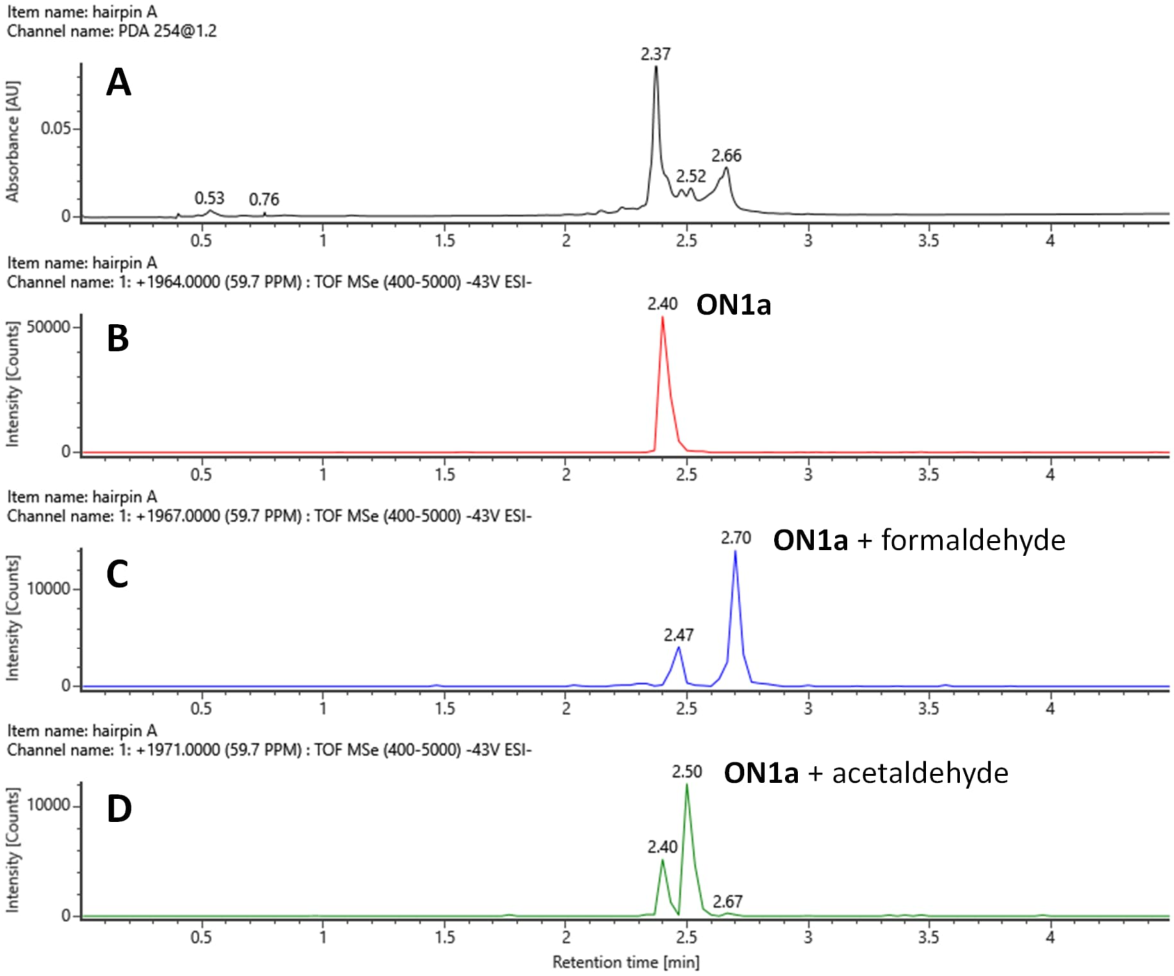

Figure S8. UV (A) and extracted ion (B—D) UPLC traces of purified hairpin oligonucleotide ON1a; ACQUITY Premier OST column (50 × 2.1 mm, 1.7 µm); flow rate = 0.4 mL min-1; linear gradient (5—25% over 4 min) of MeOH in an aqueous solution of hexafluoroisopropanol (40 mM) and triethylamine (7 mM); λ = 254 nm; T = 60 °C. Besides naked ON1a (panel B), peaks for the reversible adducts with formaldehyde (panel C) and acetaldehyde (panel D) were observed.

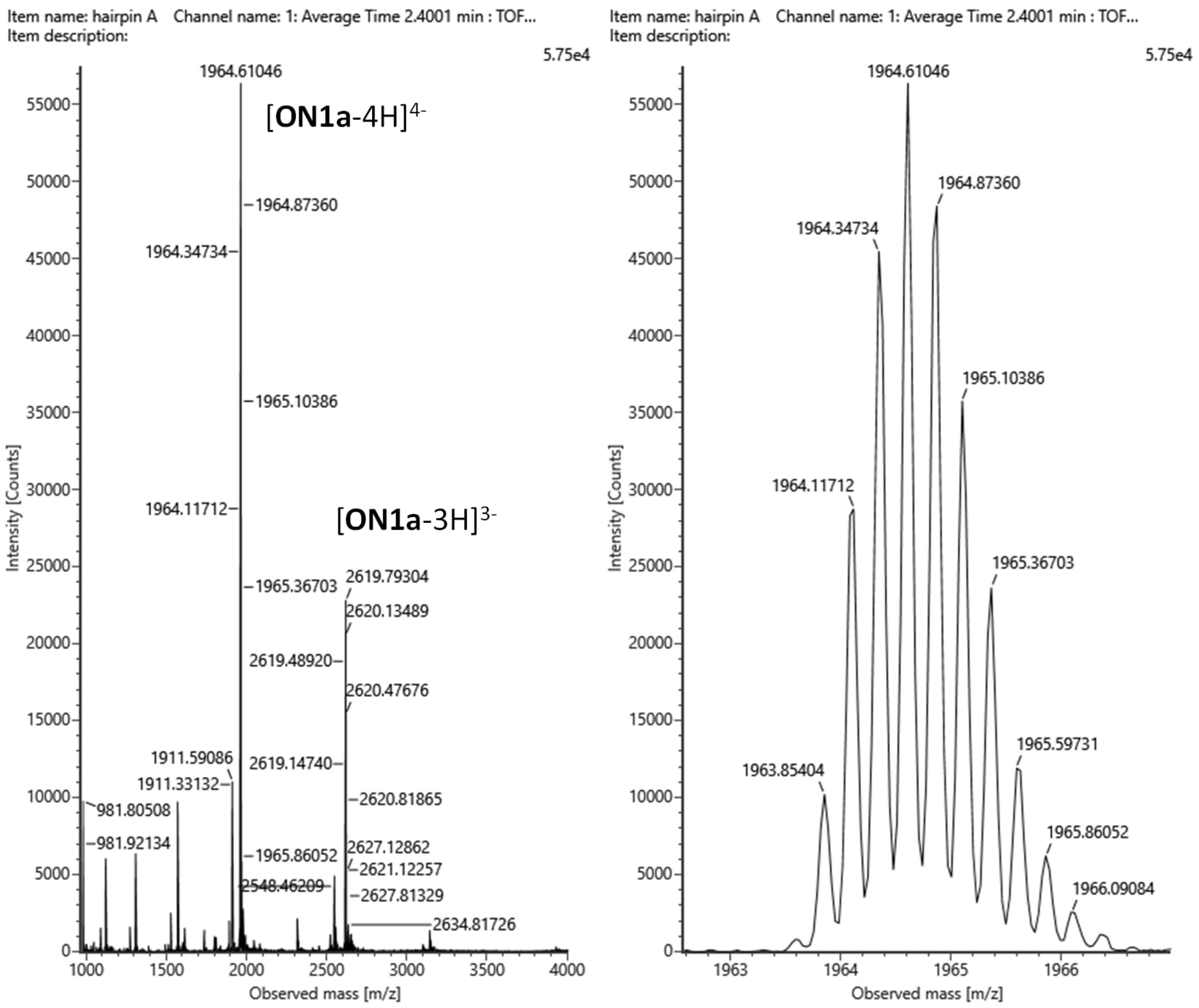

Figure S9. Mass spectrum of hairpin oligonucleotide ON1a.

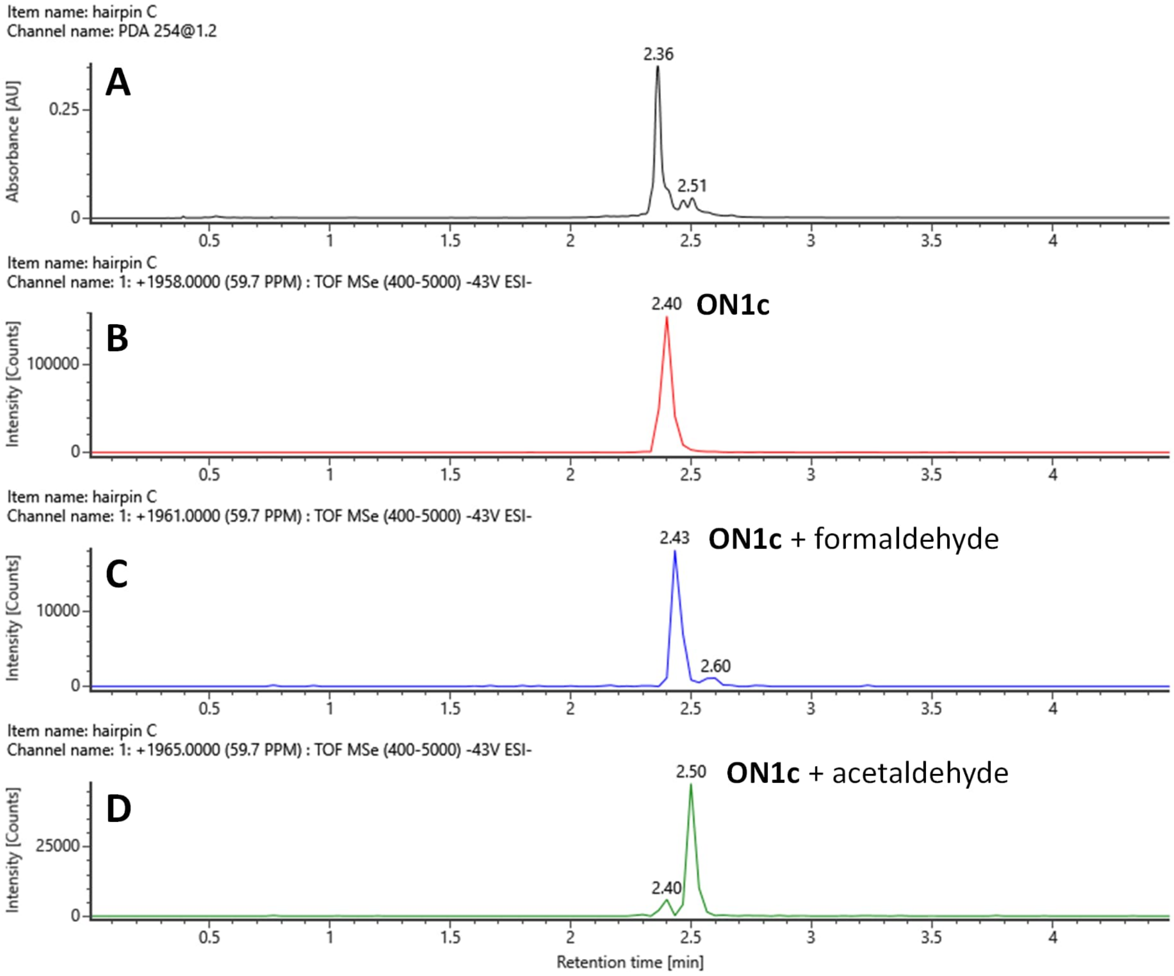

Figure S10. UV (A) and extracted ion (B—D) UPLC traces of purified hairpin oligonucleotide ON1c; ACQUITY Premier OST column (50 × 2.1 mm, 1.7 µm); flow rate = 0.4 mL min-1; linear gradient (5—25% over 4 min) of MeOH in an aqueous solution of hexafluoroisopropanol (40 mM) and triethylamine (7 mM); λ = 254 nm; T = 60 °C. Besides naked ON1c (panel B), peaks for the reversible adducts with formaldehyde (panel C) and acetaldehyde (panel D) were observed.

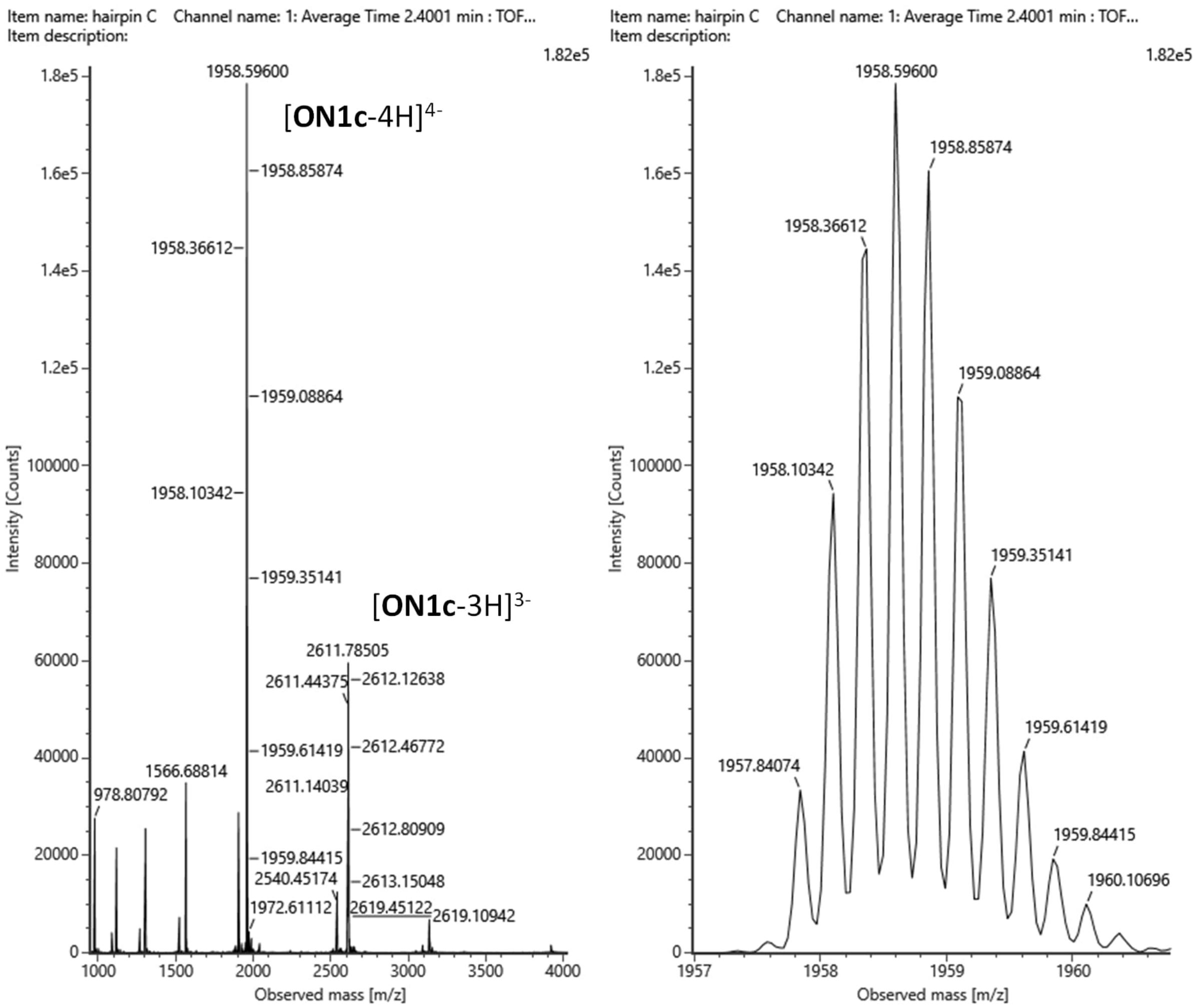

Figure S11. Mass spectrum of hairpin oligonucleotide ON1c.

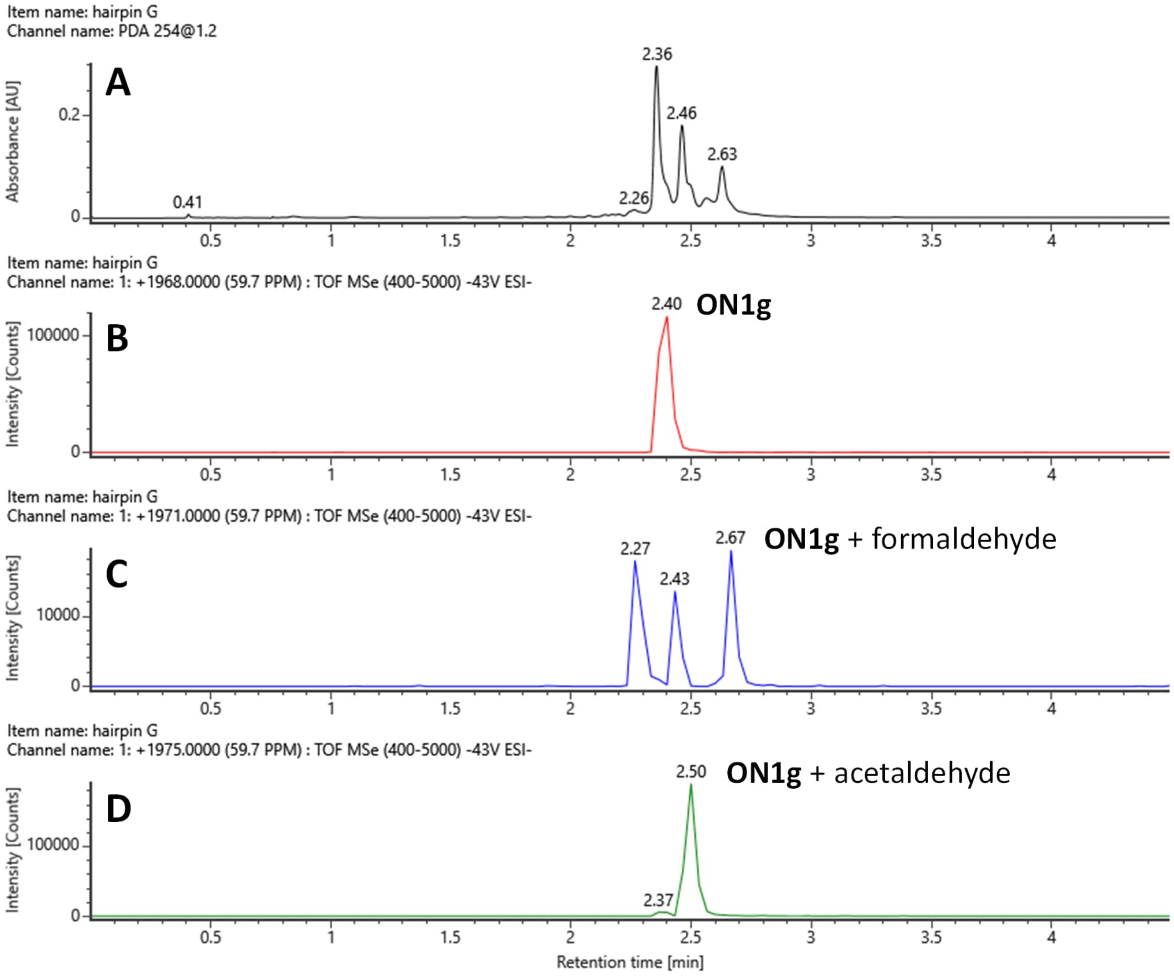

Figure S12. UV (A) and extracted ion (B—D) UPLC traces of purified hairpin oligonucleotide ON1g; ACQUITY Premier OST column (50 × 2.1 mm, 1.7 µm); flow rate = 0.4 mL min-1; linear gradient (5—25% over 4 min) of MeOH in an aqueous solution of hexafluoroisopropanol (40 mM) and triethylamine (7 mM); λ = 254 nm; T = 60 °C. Besides naked ON1g (panel B), peaks for the reversible adducts with formaldehyde (panel C) and acetaldehyde (panel D) were observed.

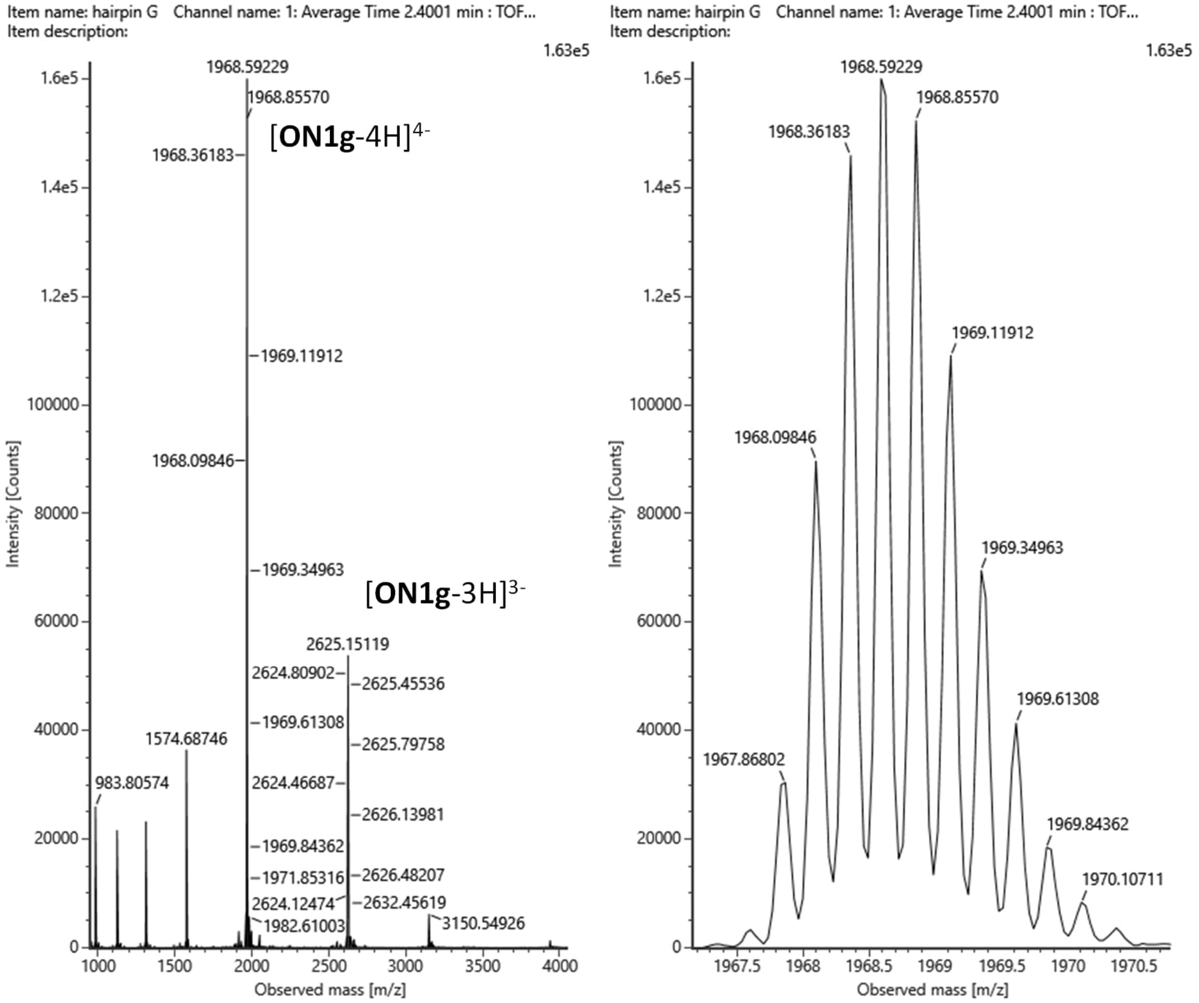

Figure S13. Mass spectrum of hairpin oligonucleotide ON1g.

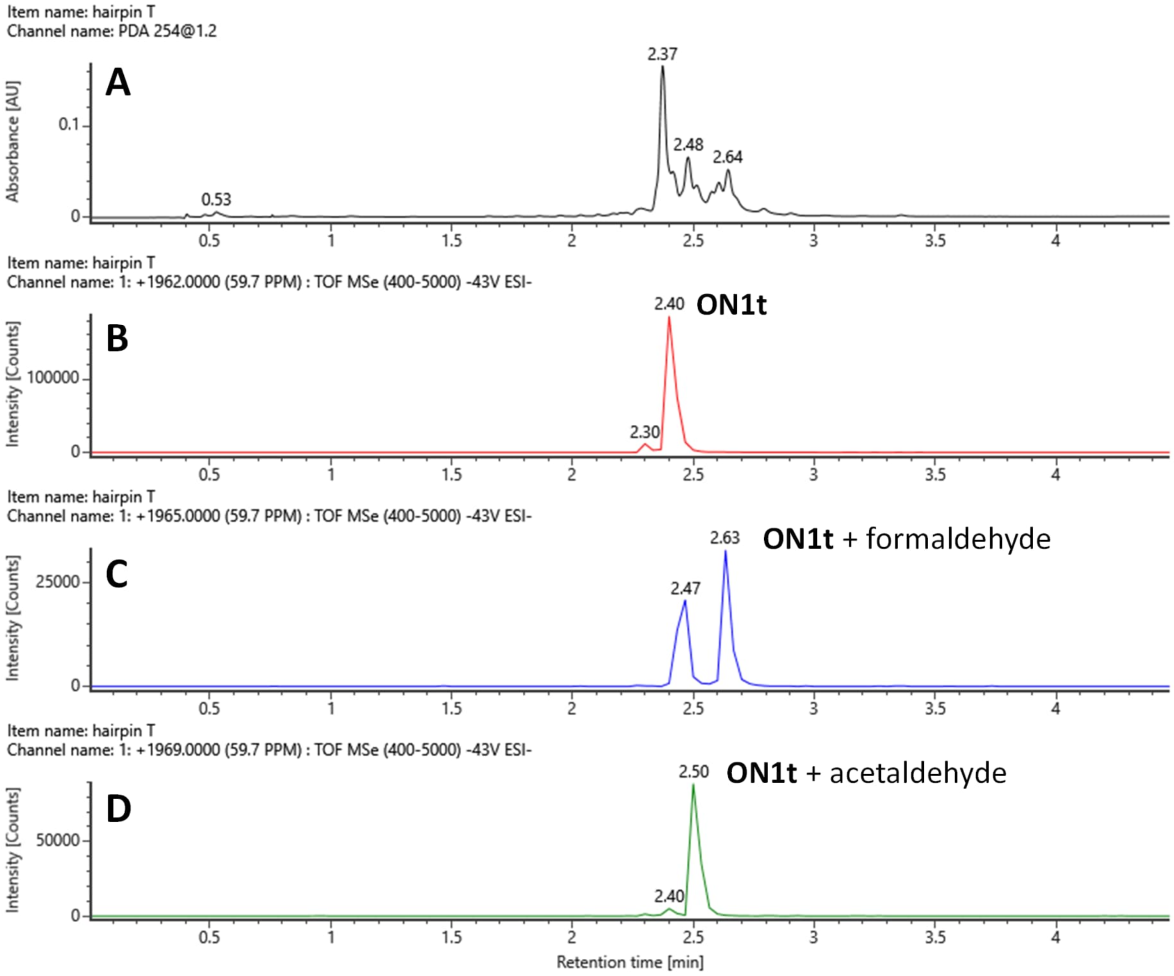

Figure S14. UV (A) and extracted ion (B—D) UPLC traces of purified hairpin oligonucleotide ON1t; ACQUITY Premier OST column (50 × 2.1 mm, 1.7 µm); flow rate = 0.4 mL min-1; linear gradient (5—25% over 4 min) of MeOH in an aqueous solution of hexafluoroisopropanol (40 mM) and triethylamine (7 mM); λ = 254 nm; T = 60 °C. Besides naked ON1t (panel B), peaks for the reversible adducts with formaldehyde (panel C) and acetaldehyde (panel D) were observed.

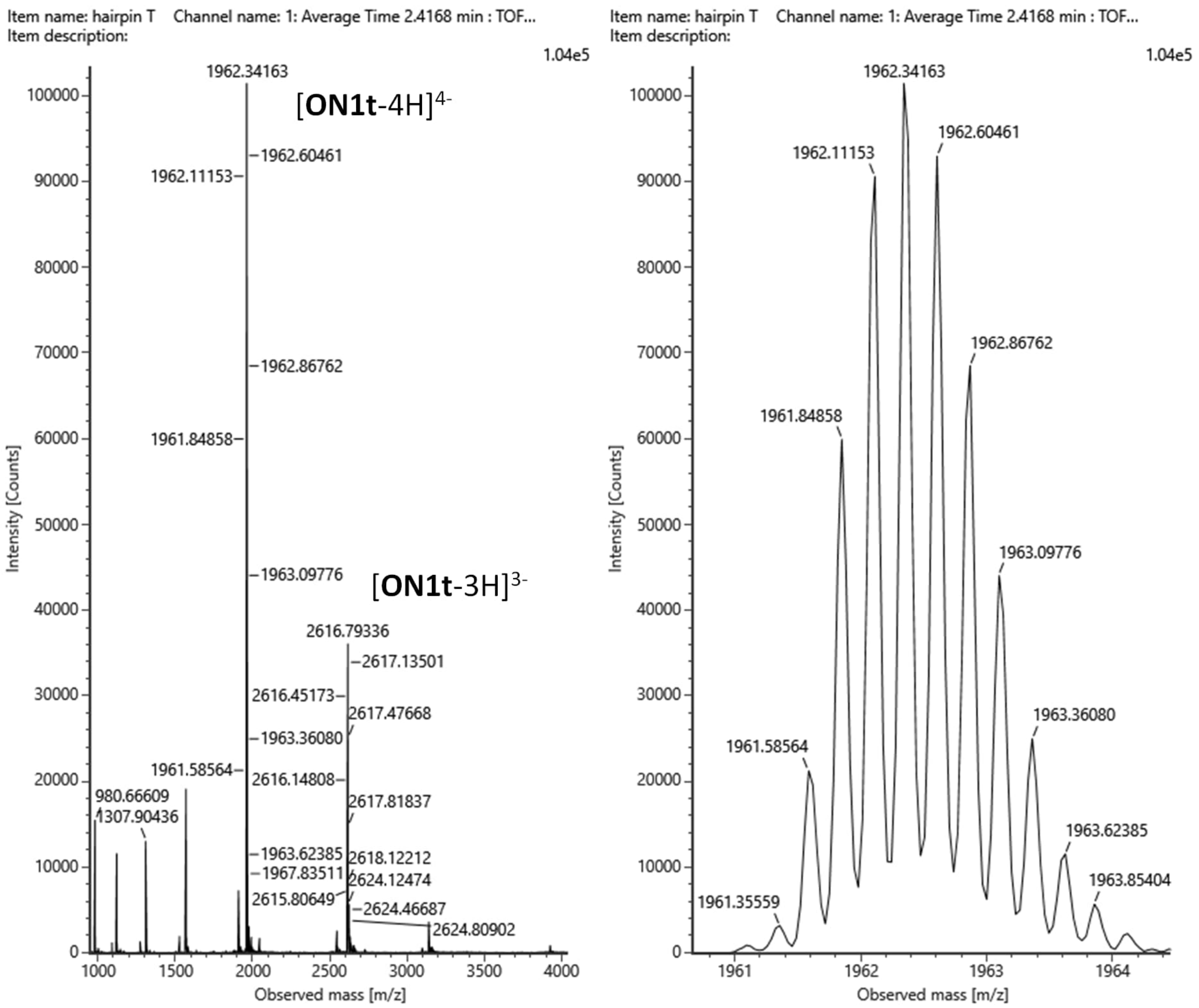

Figure S15. Mass spectrum of hairpin oligonucleotide ON1t.

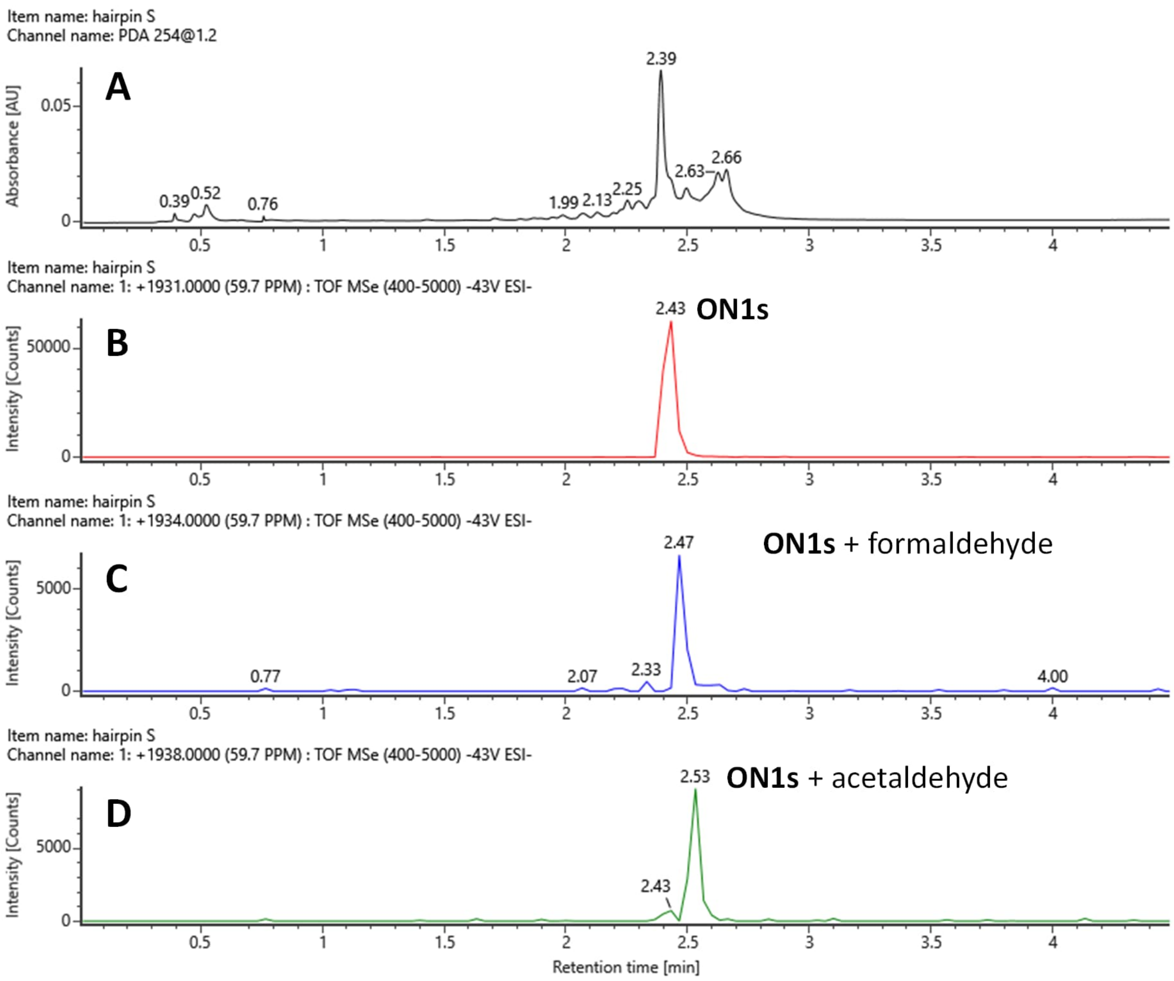

Figure S16. UV (A) and extracted ion (B—D) UPLC traces of purified hairpin oligonucleotide ON1s; ACQUITY Premier OST column (50 × 2.1 mm, 1.7 µm); flow rate = 0.4 mL min-1; linear gradient (5—25% over 4 min) of MeOH in an aqueous solution of hexafluoroisopropanol (40 mM) and triethylamine (7 mM); λ = 254 nm; T = 60 °C. Besides naked ON1s (panel B), peaks for the reversible adducts with formaldehyde (panel C) and acetaldehyde (panel D) were observed.

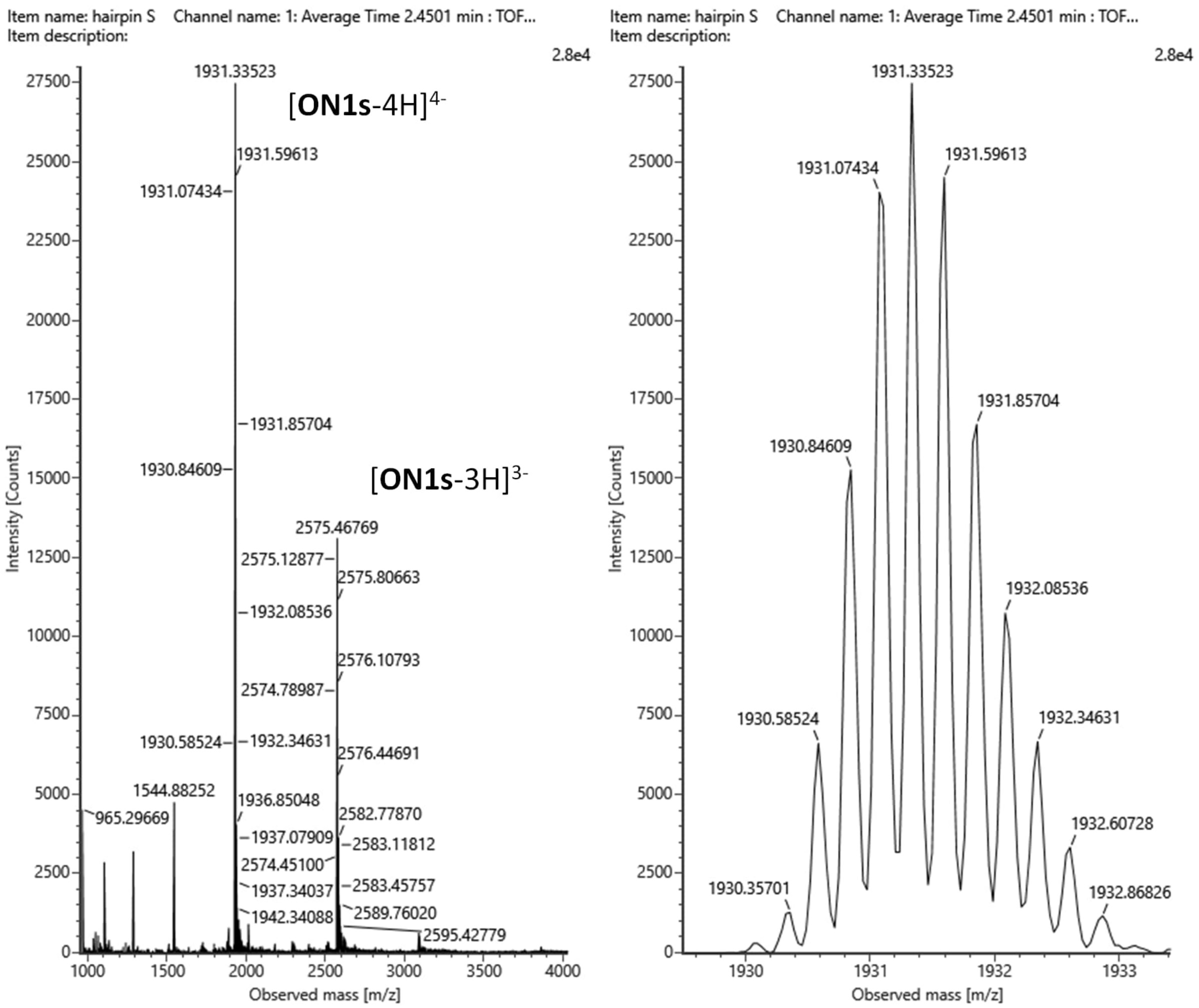

Figure S17. Mass spectrum of hairpin oligonucleotide ON1s.

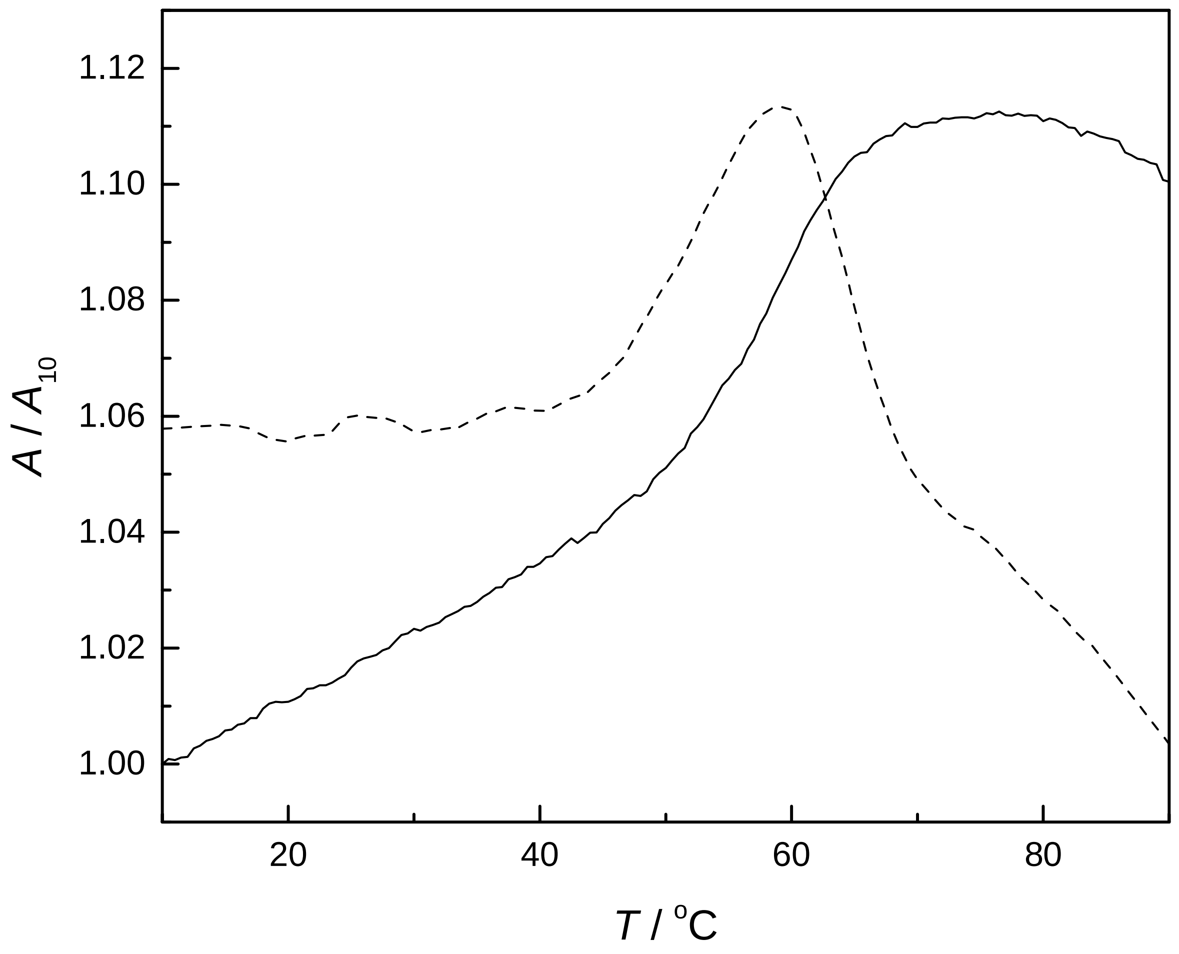

Figure S18. UV melting curve (solid line) and its first derivative (dashed line) of ON1a; pH = 5.5 (100 mM triethylammonium acetate buffer); [ON1a] = 1.0 µM.

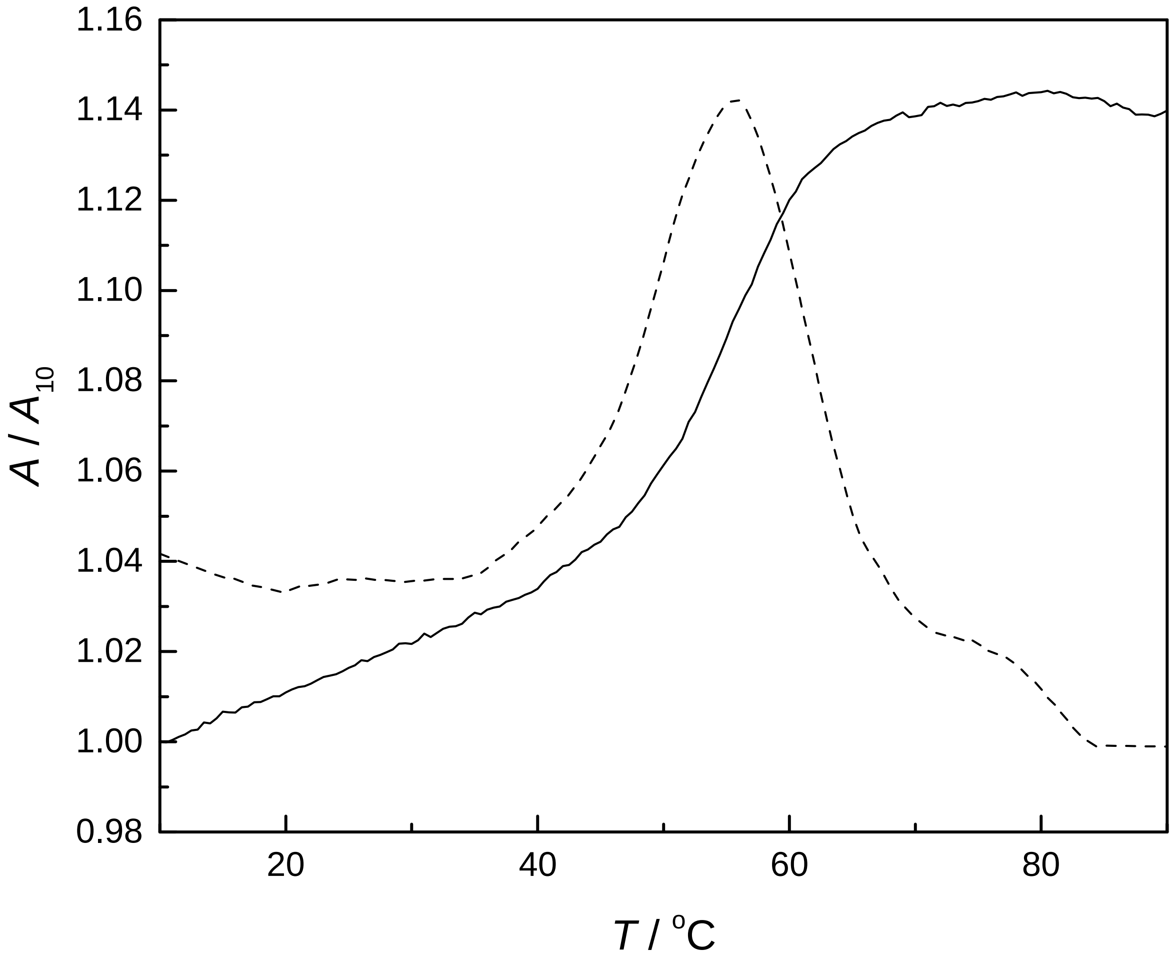

Figure S19. UV melting curve (solid line) and its first derivative (dashed line) of ON1c; pH = 5.5 (100 mM triethylammonium acetate buffer); [ON1c] = 1.0 μM.

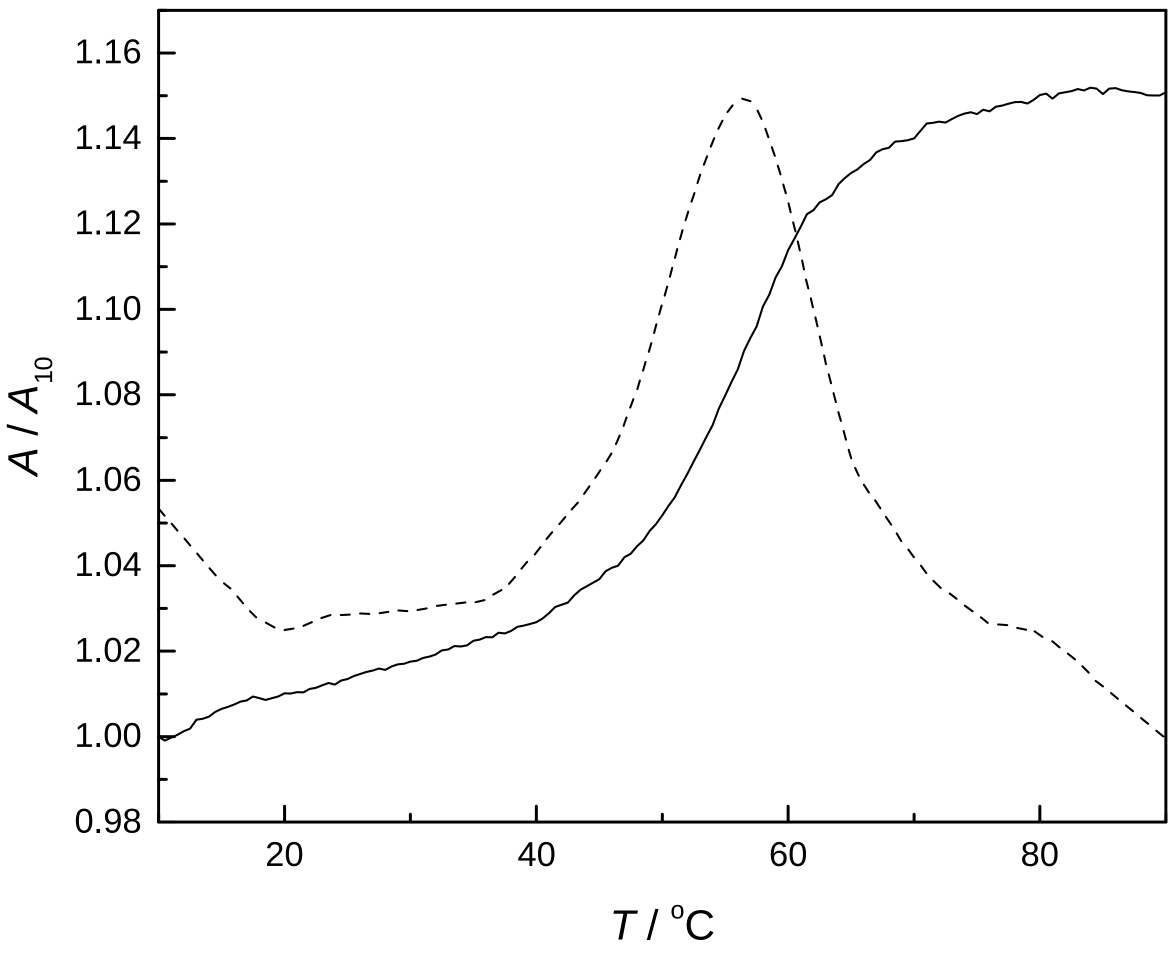

Figure S20. UV melting curve (solid line) and its first derivative (dashed line) of ON1g; pH = 5.5 (100 mM triethylammonium acetate buffer); [ON1g] = 1.0 µM.

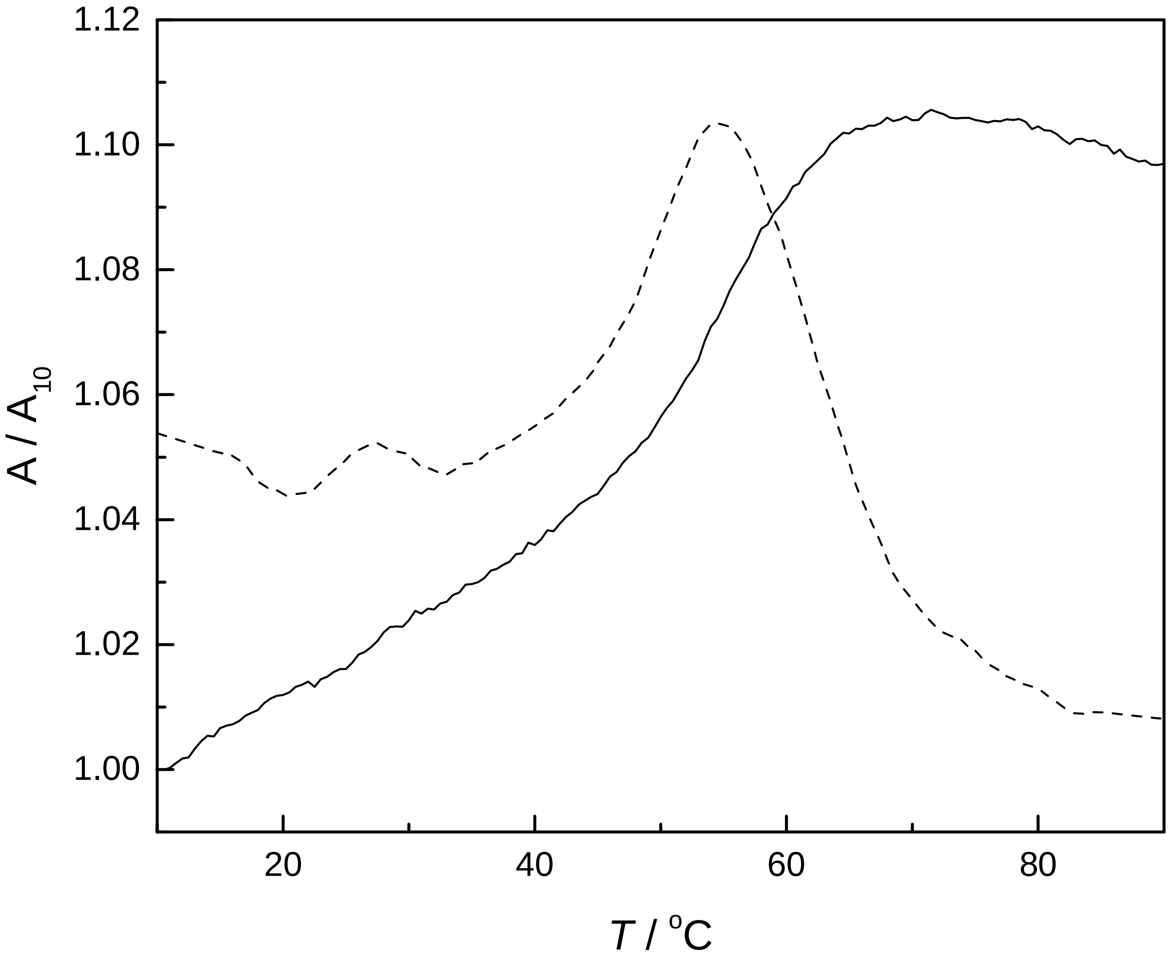

Figure S21. UV melting curve (solid line) and its first derivative (dashed line) of ON1t; pH = 5.5 (100 mM triethylammonium acetate buffer); [ON1t] = 1.0 µM.

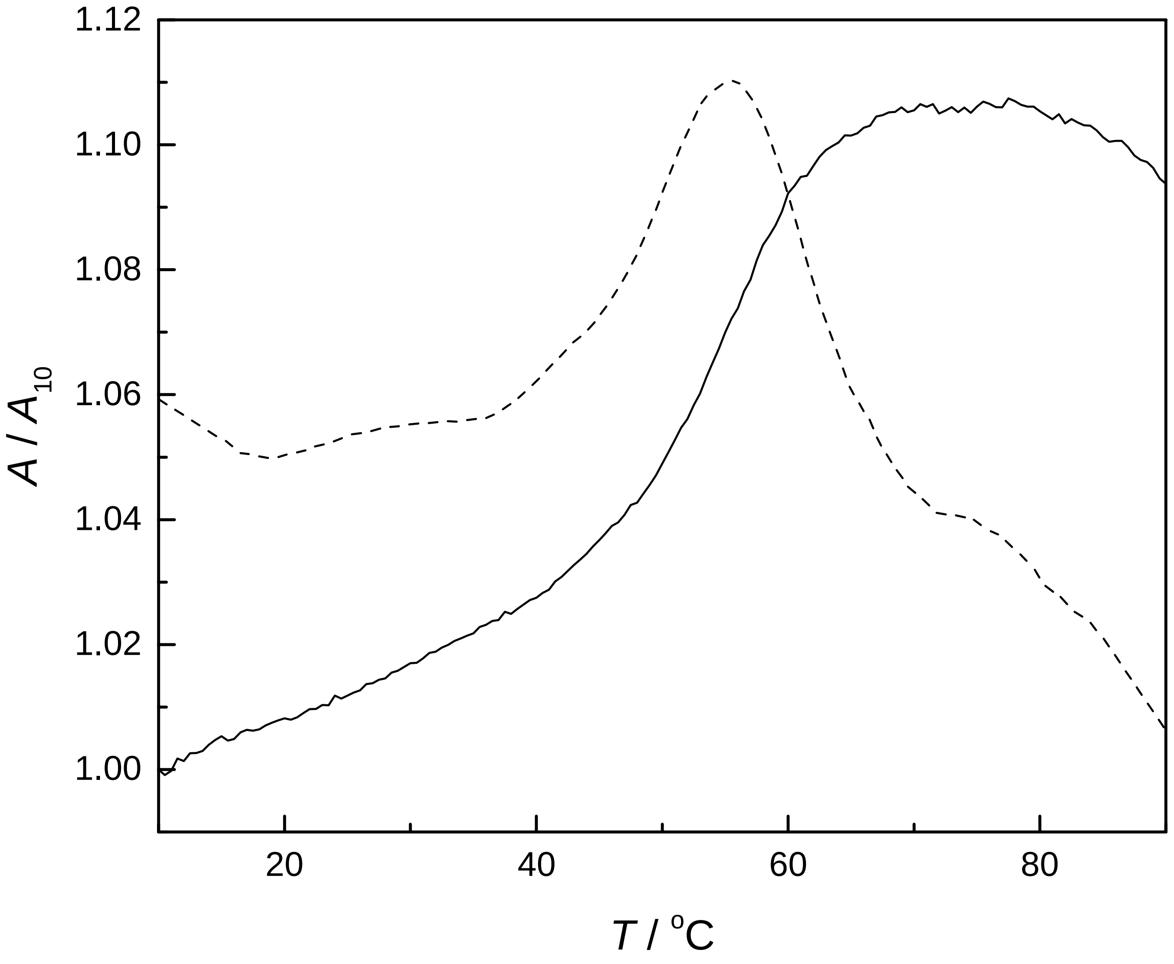

Figure S22. UV melting curve (solid line) and its first derivative (dashed line) of ON1s; pH = 5.5 (100 mM triethylammonium acetate buffer); [ON1s] = 1.0 µM.

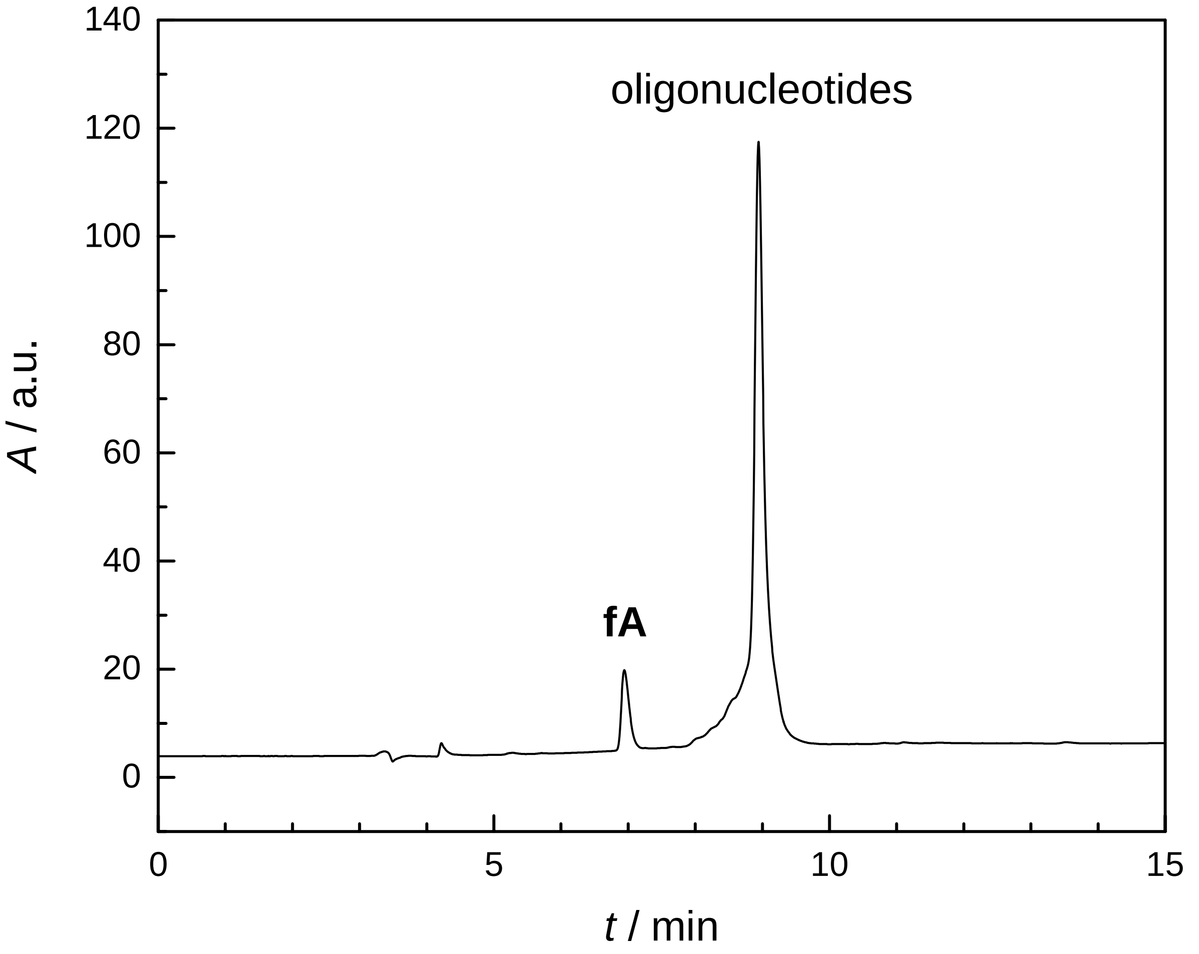

Figure S23. HPLC trace of a mixture of fA and ON1a; Thermo Scientific ODS Hypersil column (250 × 4.6 mm, 5 μm); flow rate = 1.0 mL min^{-1}; linear gradient (5—29% over 15 min) of MeCN in 50 mM aqueous triethylammonium acetate buffer (pH = 7.0); λ = 260 nm.

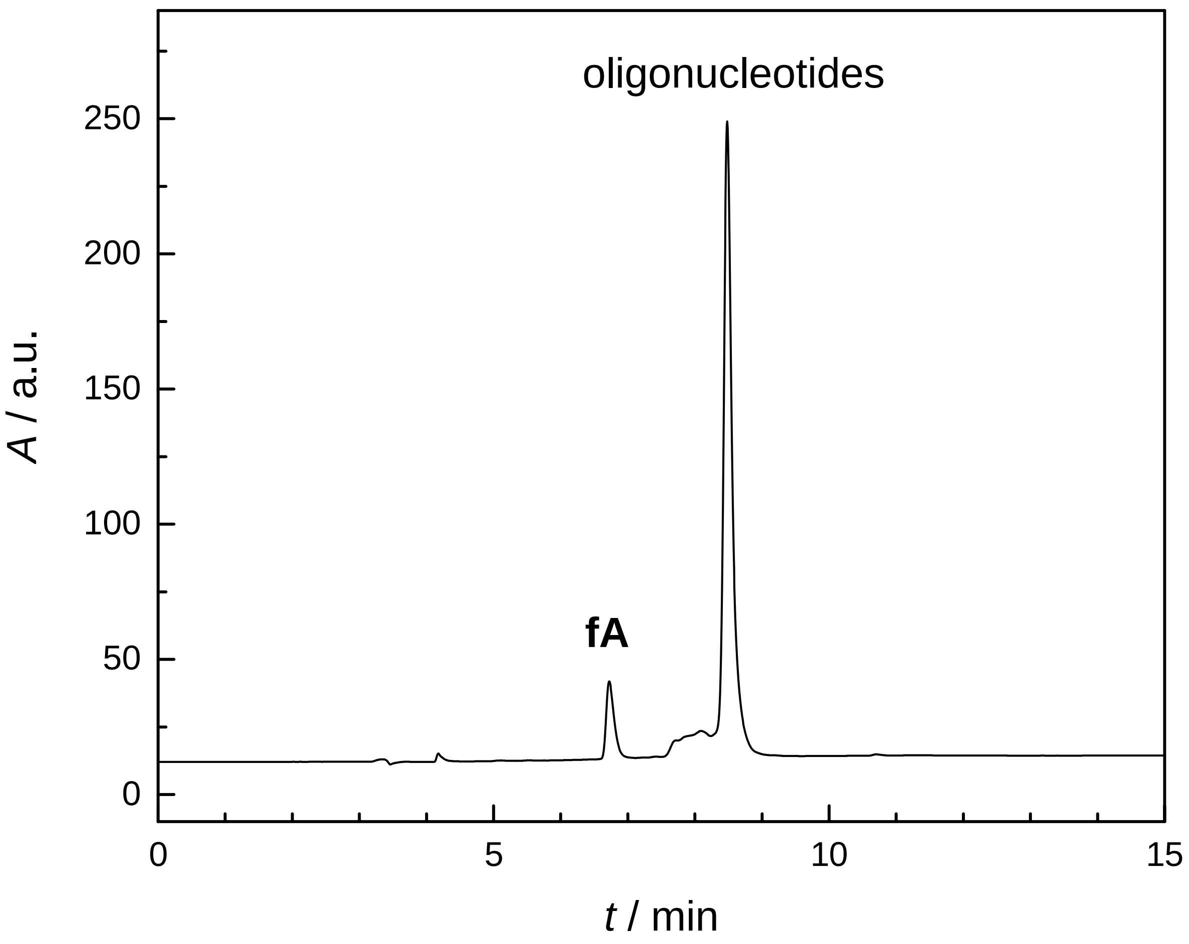

Figure S24. HPLC trace of a mixture of fA and ON1c; Thermo Scientific ODS Hypersil column (250 × 4.6 mm, 5 µm); flow rate = 1.0 mL min^{-1}; linear gradient (5—29% over 15 min) of MeCN in 50 mM aqueous triethylammonium acetate buffer (pH = 7.0); λ = 260 nm.

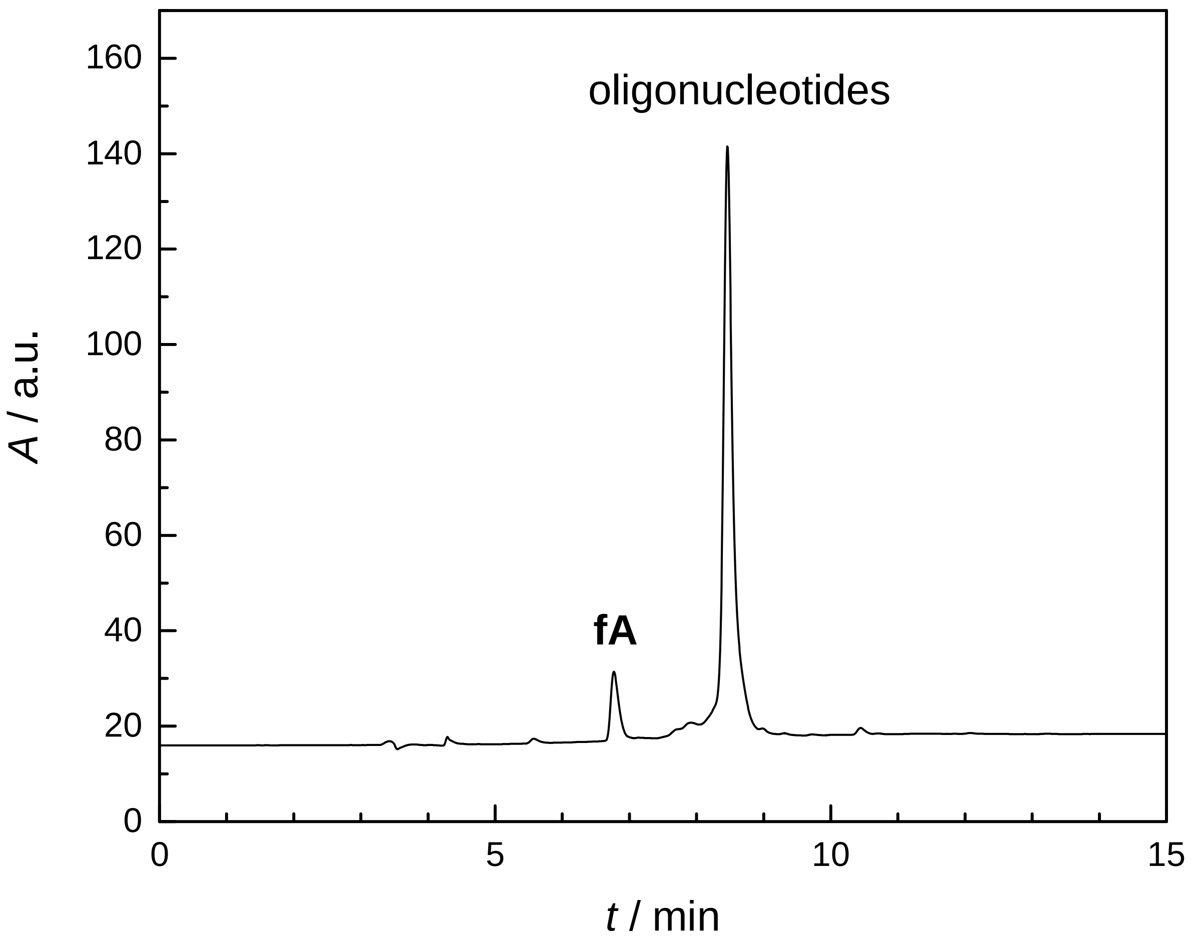

Figure S25. HPLC trace of a mixture of fA and ON1g; Thermo Scientific ODS Hypersil column (250 × 4.6 mm, 5 µm); flow rate = 1.0 mL min^{-1}; linear gradient (5—29% over 15 min) of MeCN in 50 mM aqueous triethylammonium acetate buffer (pH = 7.0); λ = 260 nm.

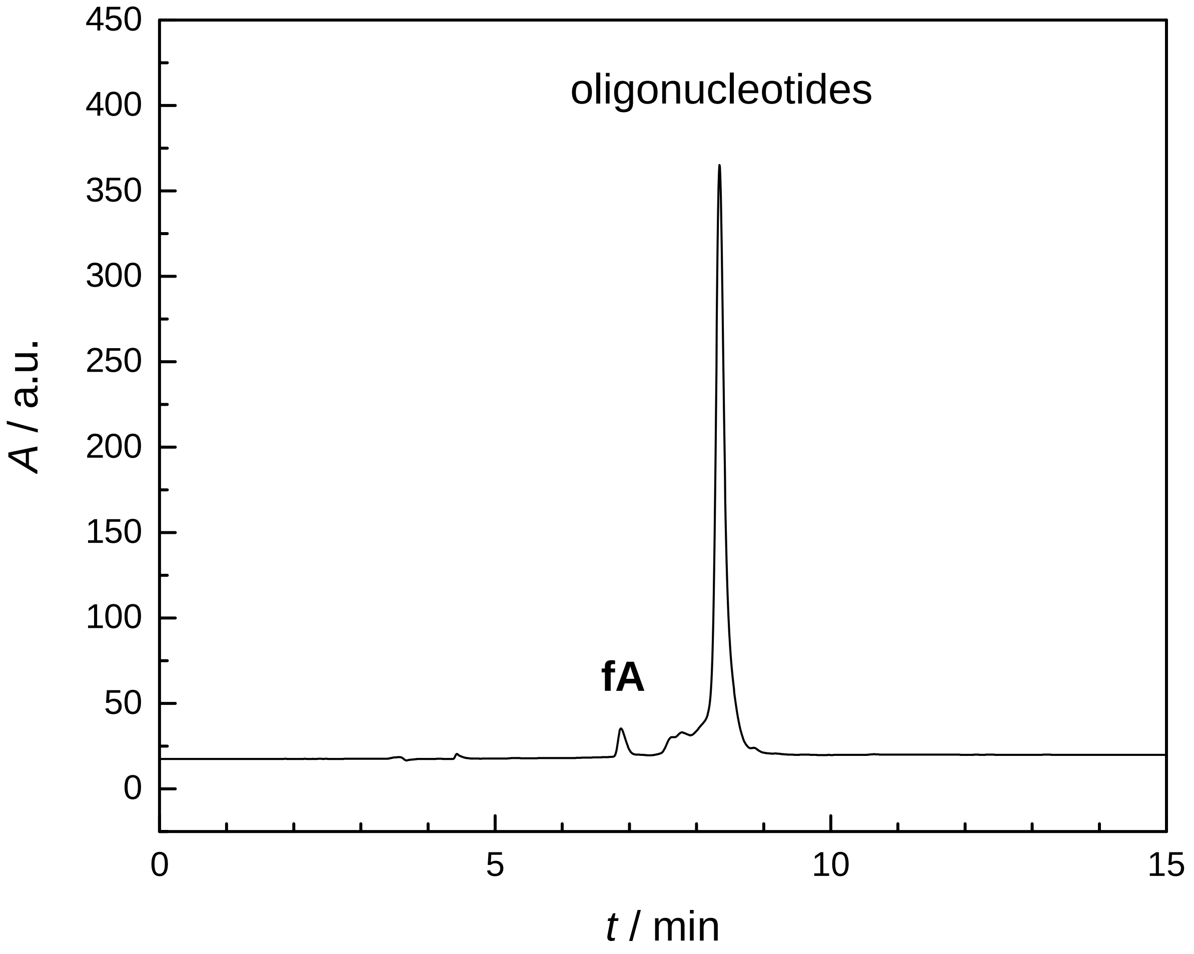

Figure S26. HPLC trace of a mixture of fA and ON1t; Thermo Scientific ODS Hypersil column (250 × 4.6 mm, 5 μm); flow rate = 1.0 mL min^{-1}; linear gradient (5—29% over 15 min) of MeCN in 50 mM aqueous triethylammonium acetate buffer (pH = 7.0); λ = 260 nm.

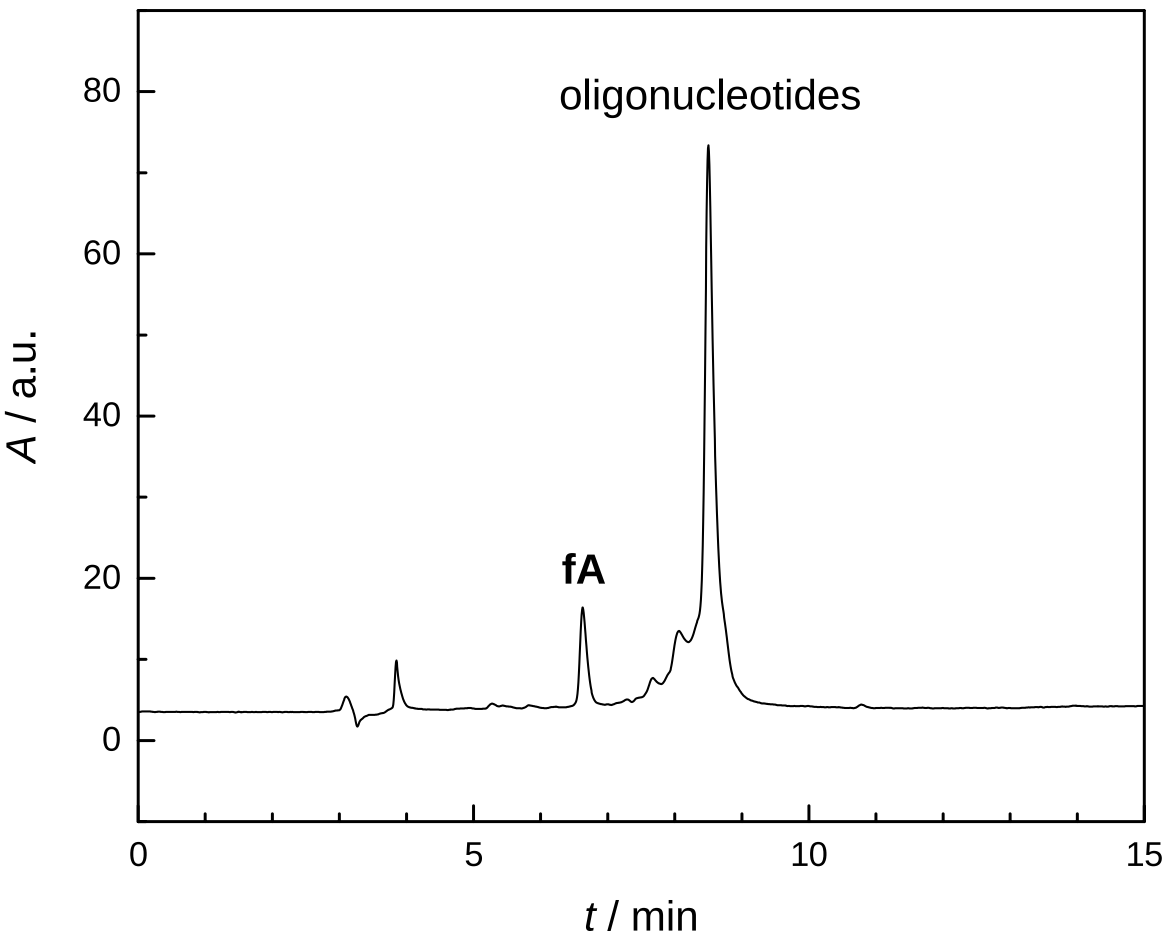

Figure S27. HPLC trace of a mixture of fA and ON1s; Thermo Scientific ODS Hypersil column (250 × 4.6 mm, 5 µm); flow rate = 1.0 mL min^{-1}; linear gradient (5—29% over 15 min) of MeCN in 50 mM aqueous triethylammonium acetate buffer (pH = 7.0); λ = 260 nm.

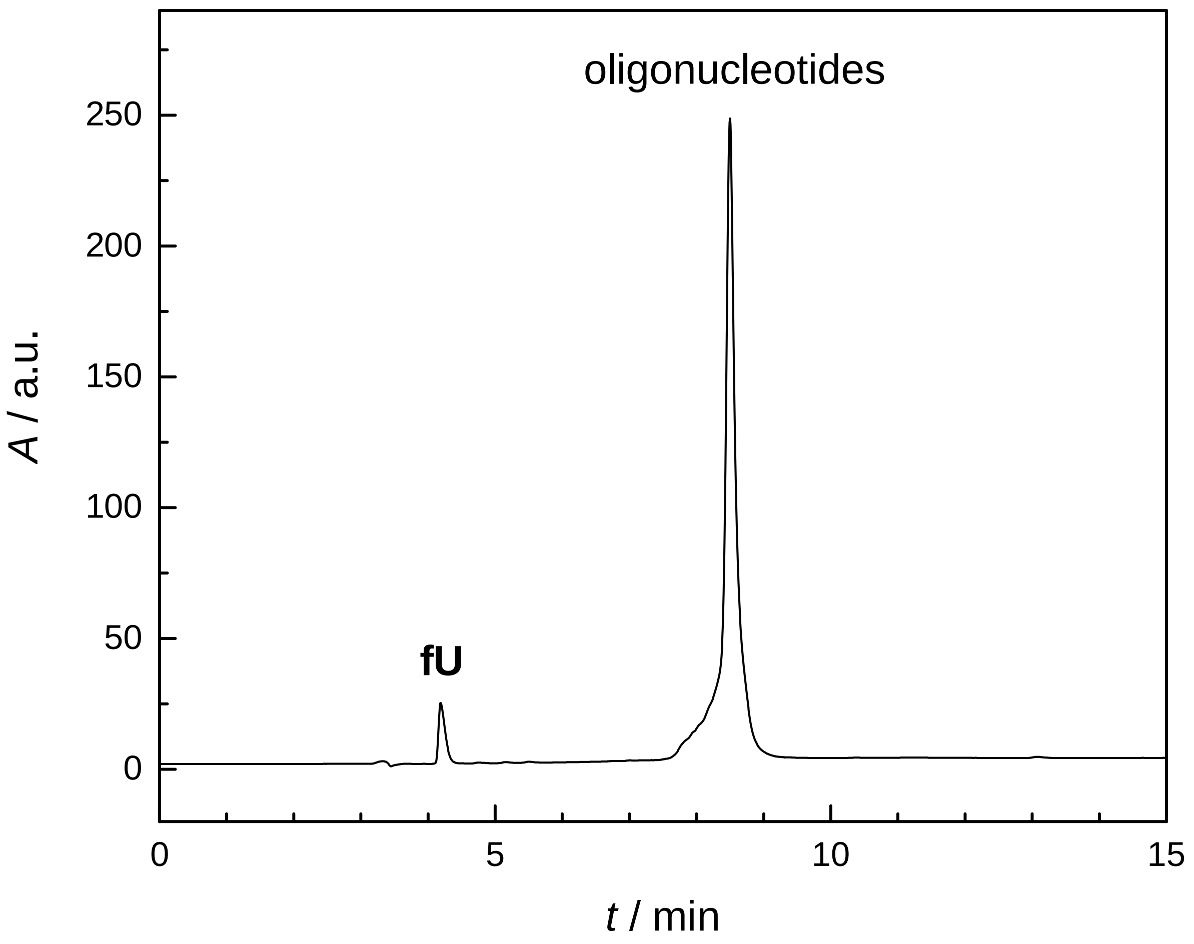

Figure S28. HPLC trace of a mixture of fU and ON1a; Thermo Scientific ODS Hypersil column (250 × 4.6 mm, 5 µm); flow rate = 1.0 mL min^{-1}; linear gradient (5—29% over 15 min) of MeCN in 50 mM aqueous triethylammonium acetate buffer (pH = 7.0); λ = 260 nm.

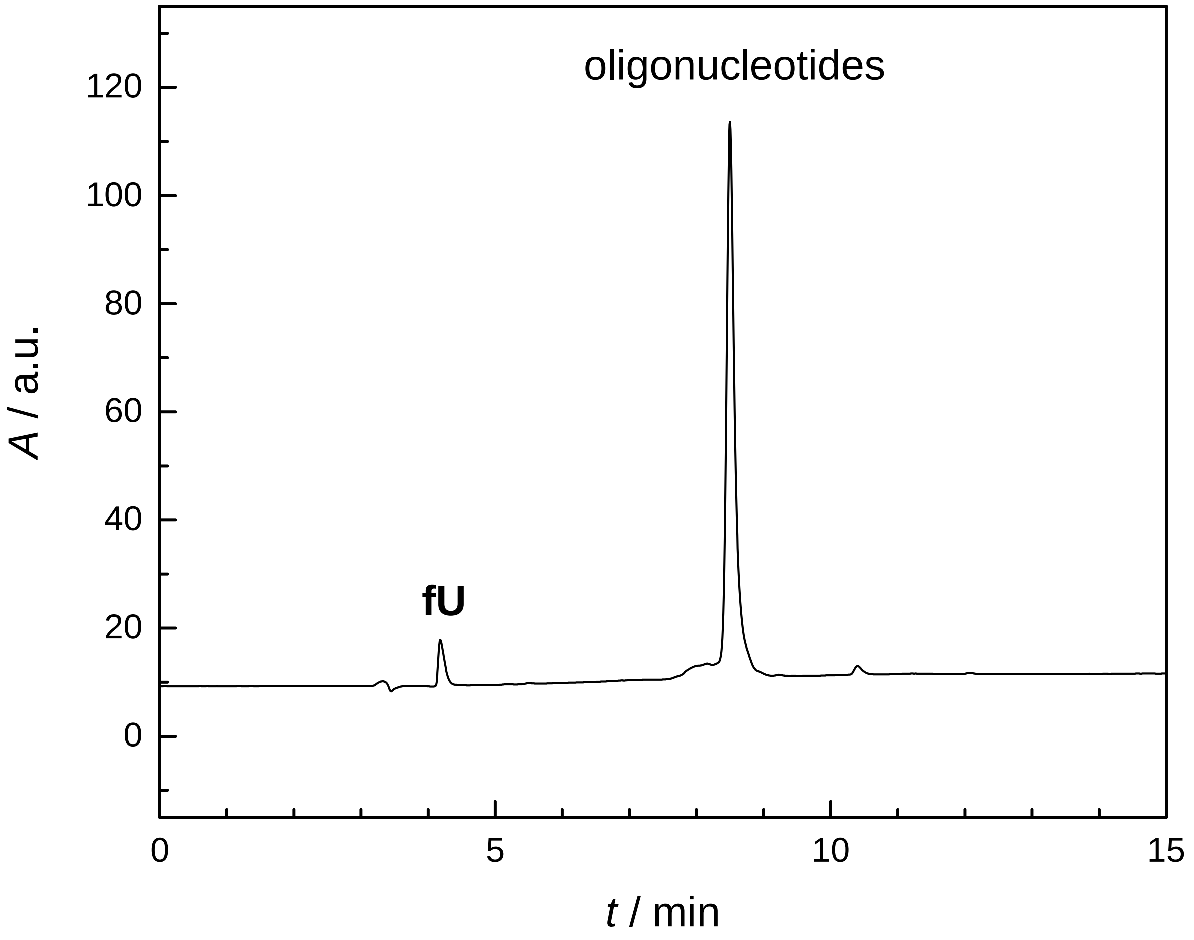

Figure S29. HPLC trace of a mixture of fU and ON1c; Thermo Scientific ODS Hypersil column (250 × 4.6 mm, 5 μm); flow rate = 1.0 mL min^{-1}; linear gradient (5—29% over 15 min) of MeCN in 50 mM aqueous triethylammonium acetate buffer (pH = 7.0); λ = 260 nm.

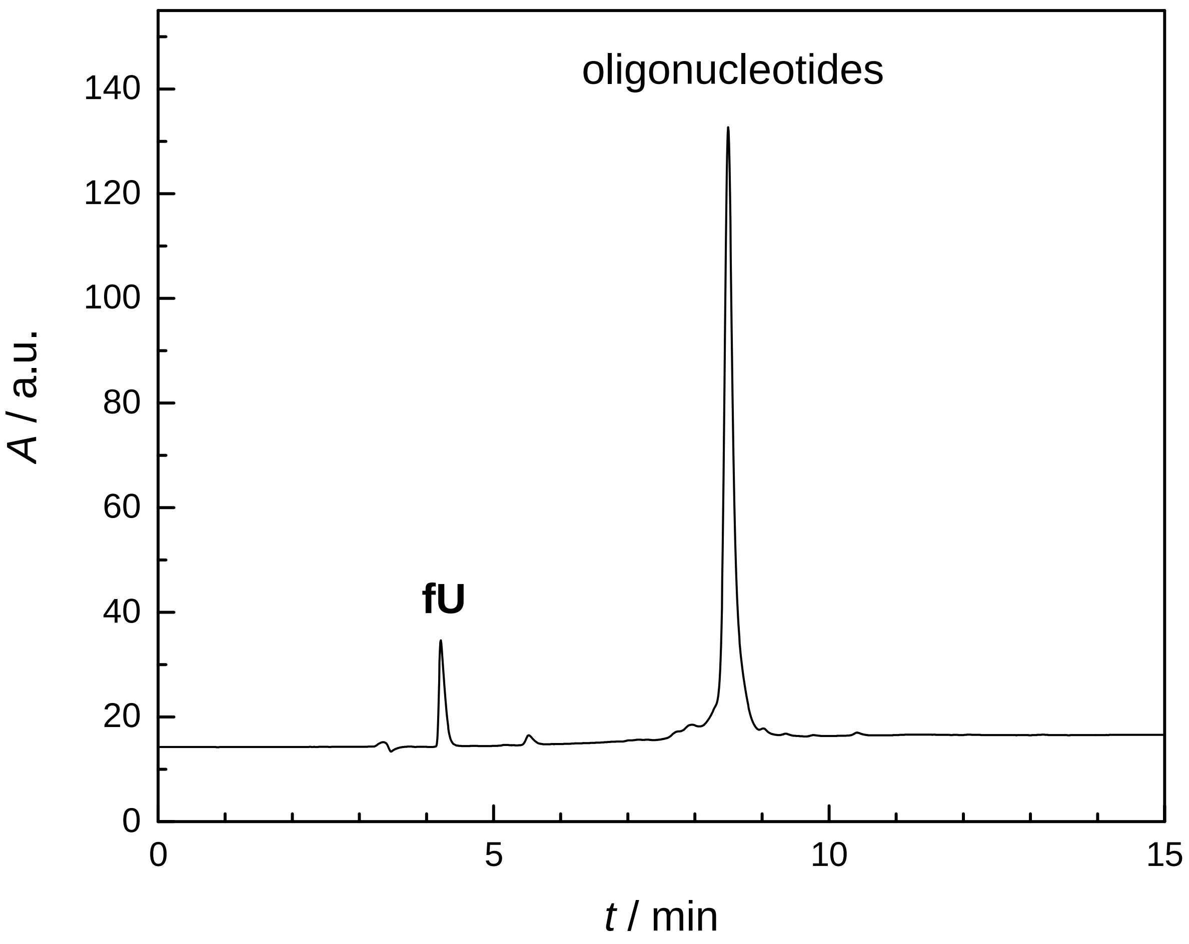

Figure S30. HPLC trace of a mixture of fU and ON1g; Thermo Scientific ODS Hypersil column (250 × 4.6 mm, 5 µm); flow rate = 1.0 mL min^{-1}; linear gradient (5—29% over 15 min) of MeCN in 50 mM aqueous triethylammonium acetate buffer (pH = 7.0); λ = 260 nm.

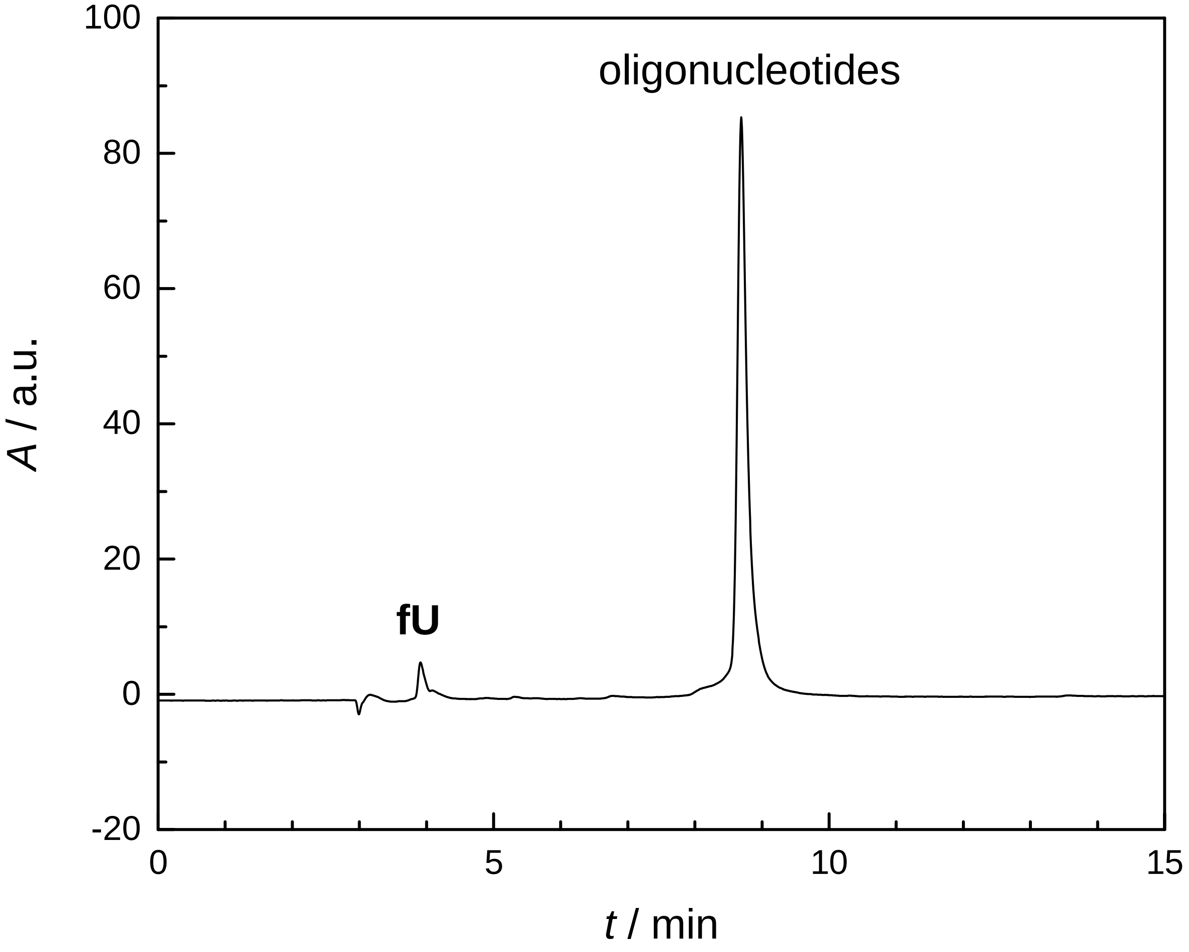

Figure S31. HPLC trace of a mixture of fU and ON1t; Thermo Scientific ODS Hypersil column (250 × 4.6 mm, 5 µm); flow rate = 1.0 mL min^{-1}; linear gradient (5—29% over 15 min) of MeCN in 50 mM aqueous triethylammonium acetate buffer (pH = 7.0); λ = 260 nm.

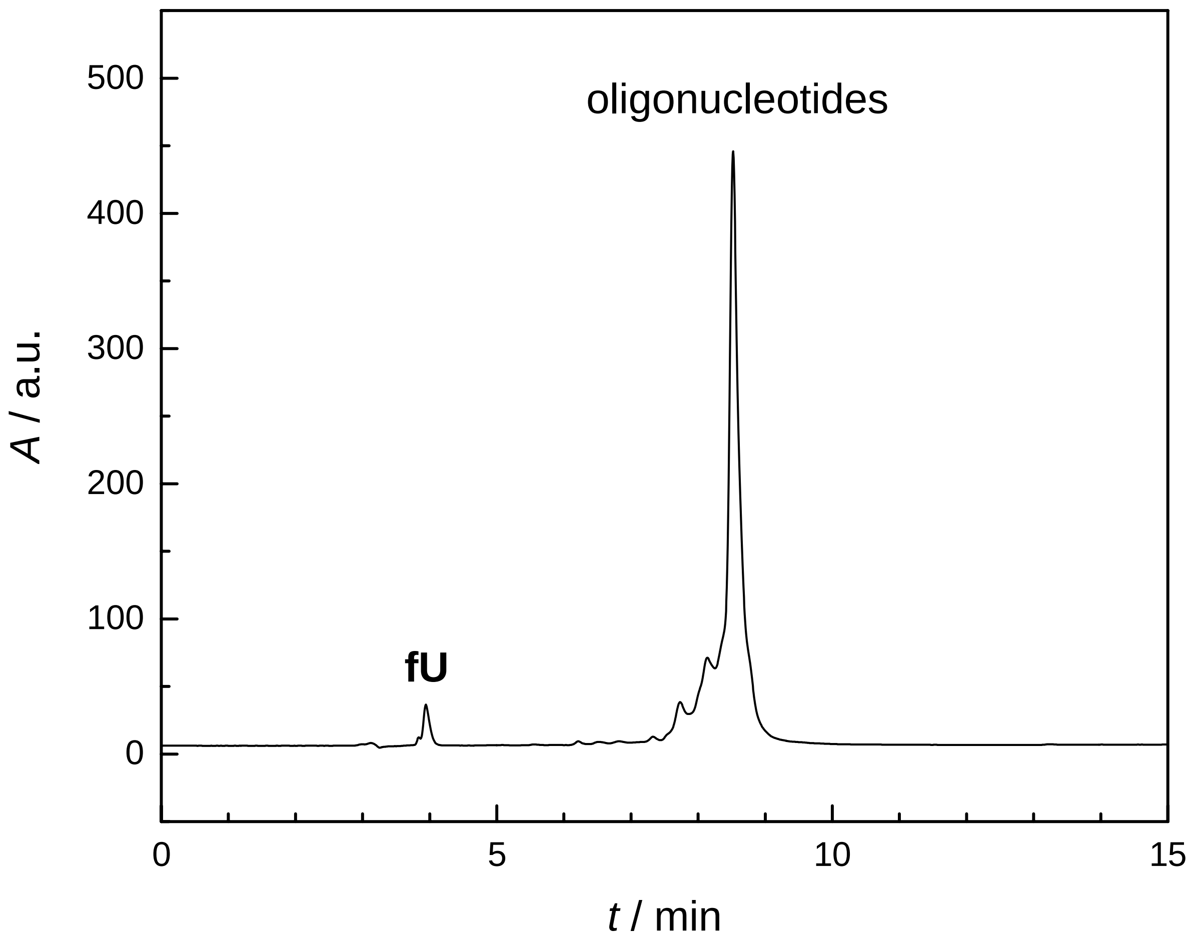

Figure S32. HPLC trace of a mixture of fU and ON1s; Thermo Scientific ODS Hypersil column (250 × 4.6 mm, 5 µm); flow rate = 1.0 mL min^{-1}; linear gradient (5—29% over 15 min) of MeCN in 50 mM aqueous triethylammonium acetate buffer (pH = 7.0); λ = 260 nm.

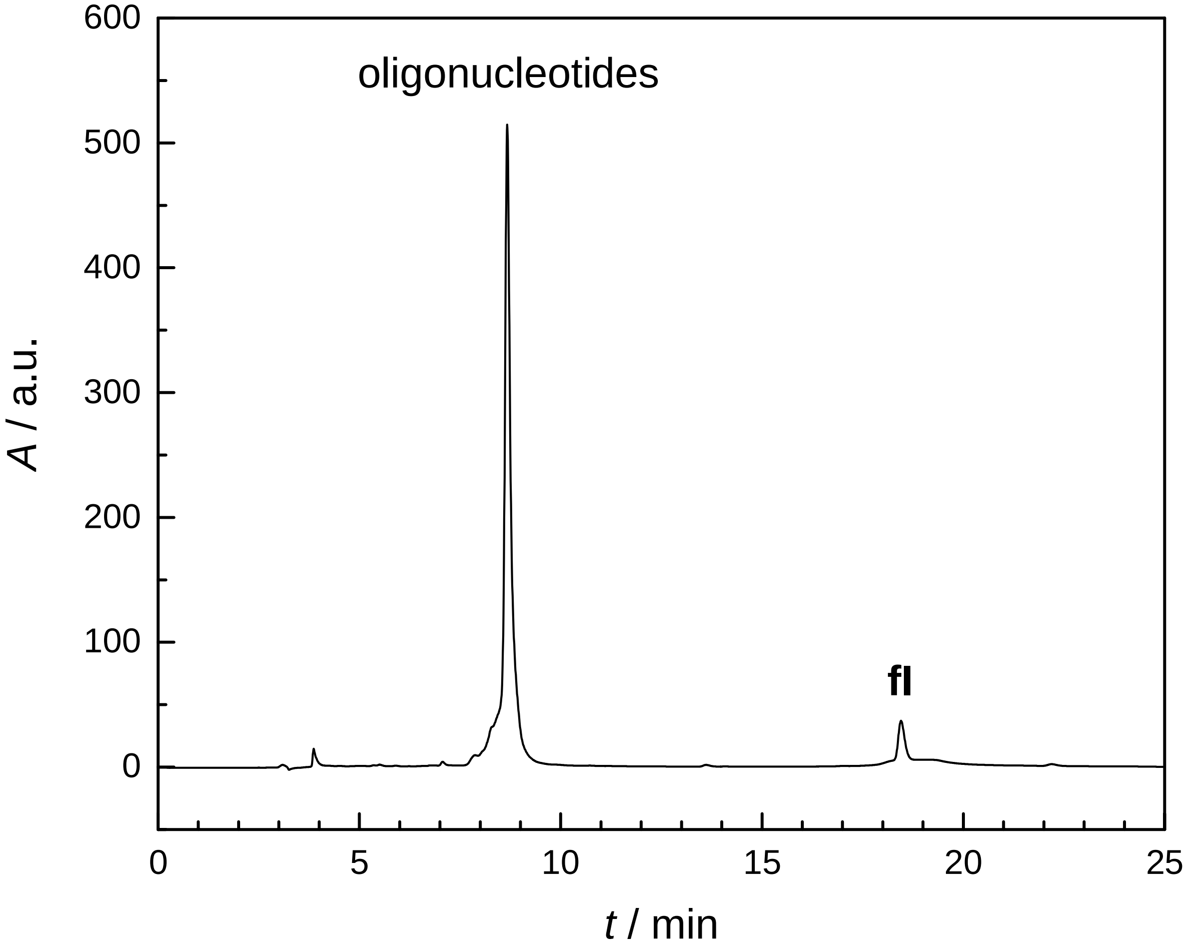

Figure S33. HPLC trace of a mixture of fI and ON1a; Thermo Scientific ODS Hypersil column (250 × 4.6 mm, 5 µm); flow rate = 1.0 mL min^{-1}; linear gradient (5—29% over 15 min, followed by 29—40% over 5 min) of MeCN in 50 mM aqueous triethylammonium acetate buffer (pH = 7.0); λ = 300 nm.

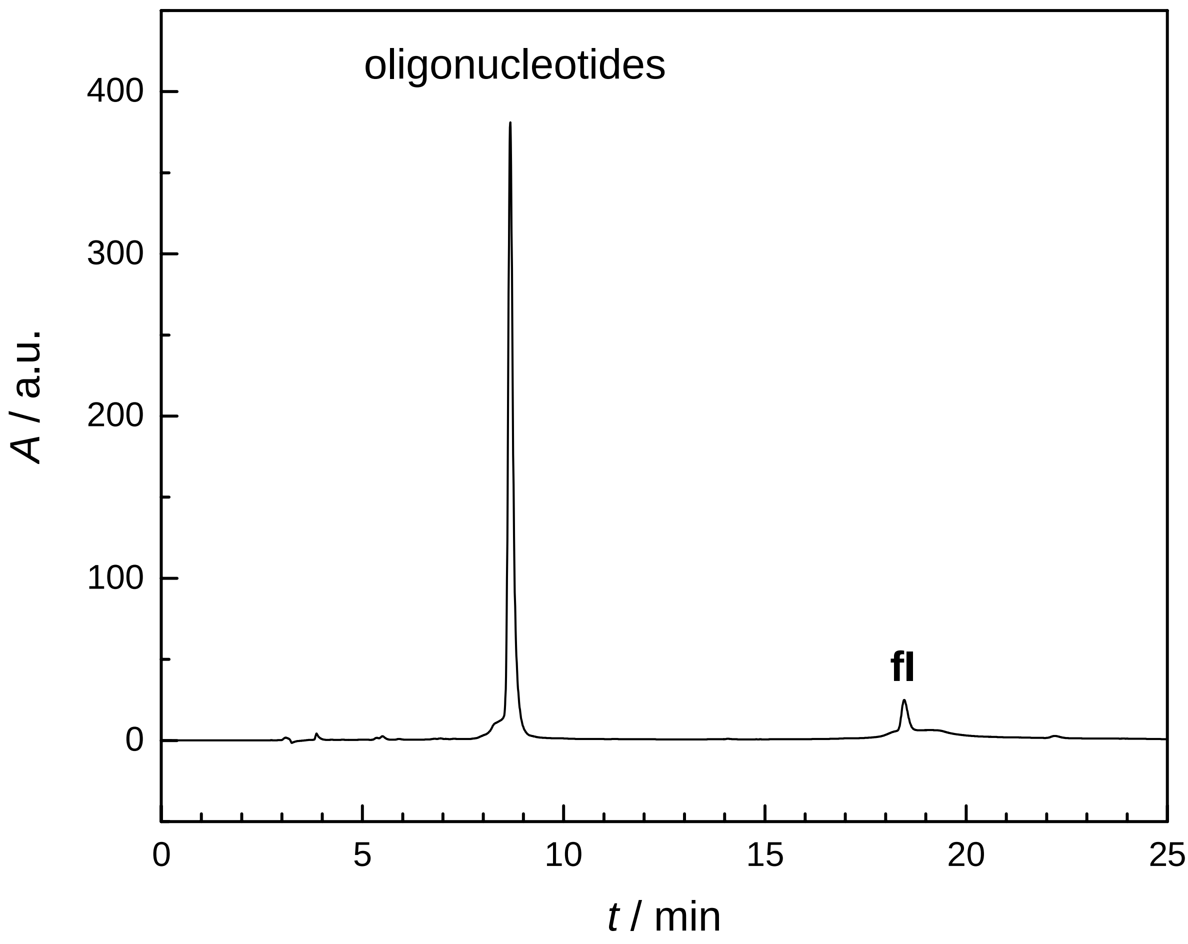

Figure S34. HPLC trace of a mixture of fI and ON1c; Thermo Scientific ODS Hypersil column (250 × 4.6 mm, 5 µm); flow rate = 1.0 mL min^{-1}; linear gradient (5—29% over 15 min, followed by 29—40% over 5 min) of MeCN in 50 mM aqueous triethylammonium acetate buffer (pH = 7.0); λ = 300 nm.

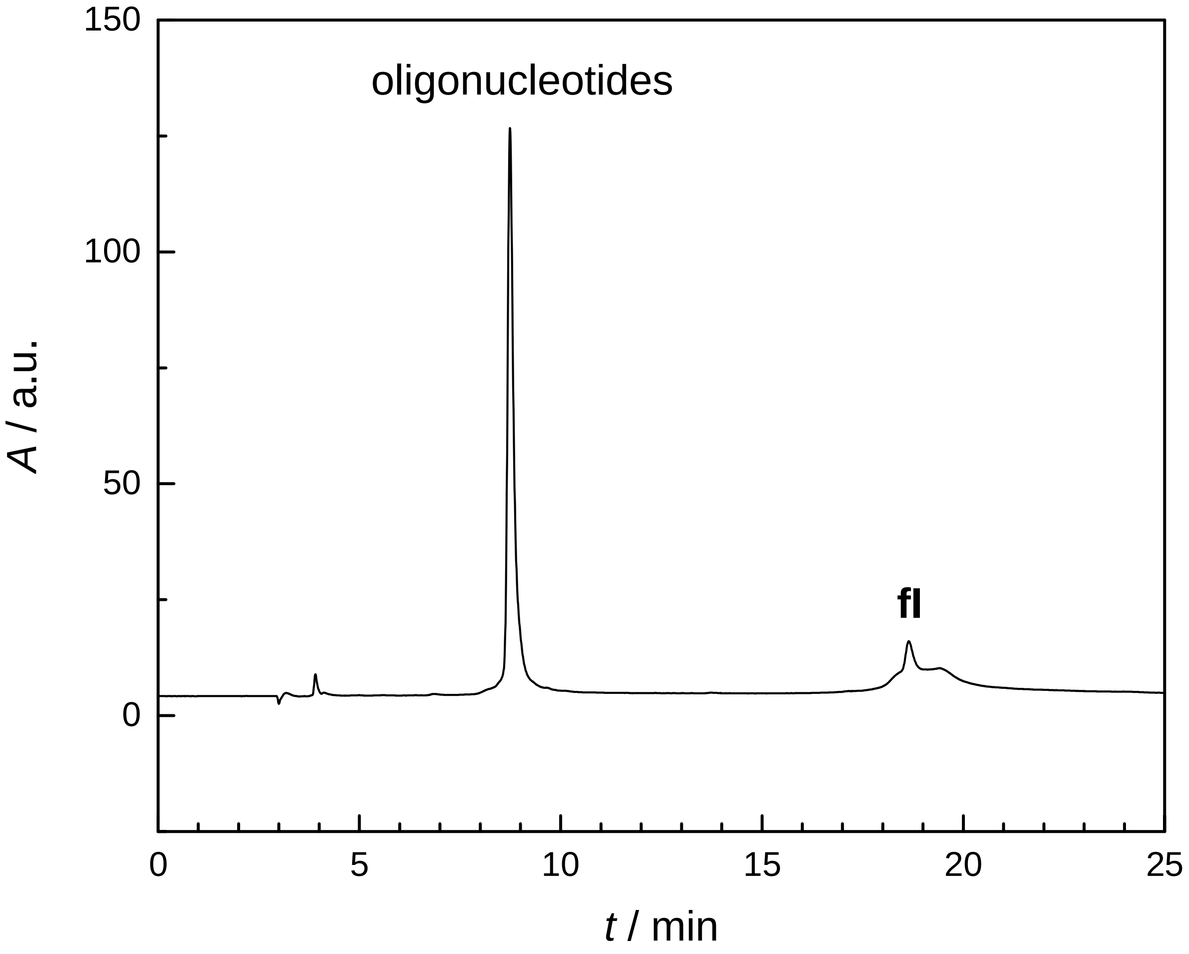

Figure S35. HPLC trace of a mixture of fI and ON1g; Thermo Scientific ODS Hypersil column (250 × 4.6 mm, 5 μm); flow rate = 1.0 mL min^{-1}; linear gradient (5—29% over 15 min, followed by 29—40% over 5 min) of MeCN in 50 mM aqueous triethylammonium acetate buffer (pH = 7.0); λ = 300 nm.

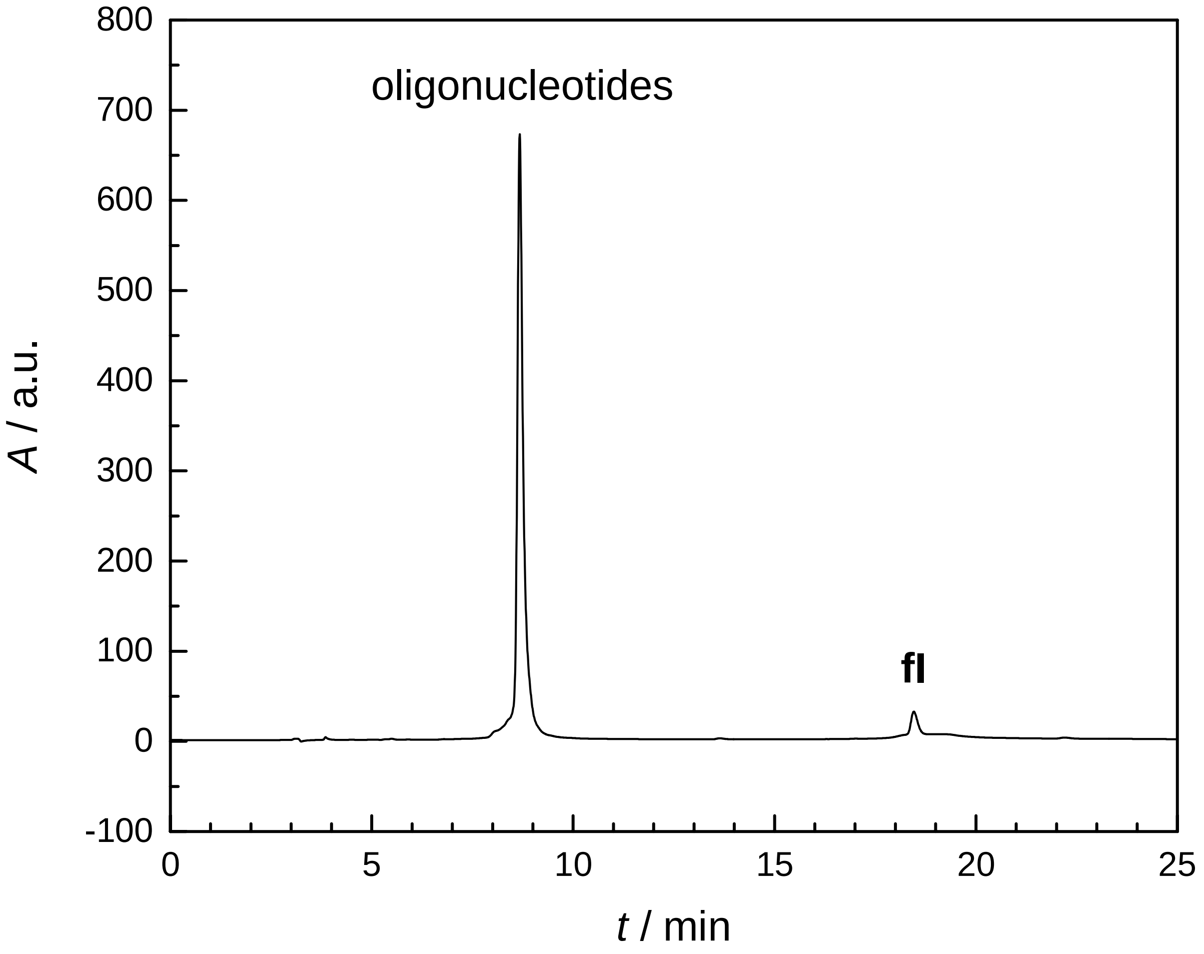

Figure S36. HPLC trace of a mixture of fI and ON1t; Thermo Scientific ODS Hypersil column (250 × 4.6 mm, 5 μm); flow rate = 1.0 mL min^{-1}; linear gradient (5—29% over 15 min, followed by 29—40% over 5 min) of MeCN in 50 mM aqueous triethylammonium acetate buffer (pH = 7.0); λ = 300 nm.

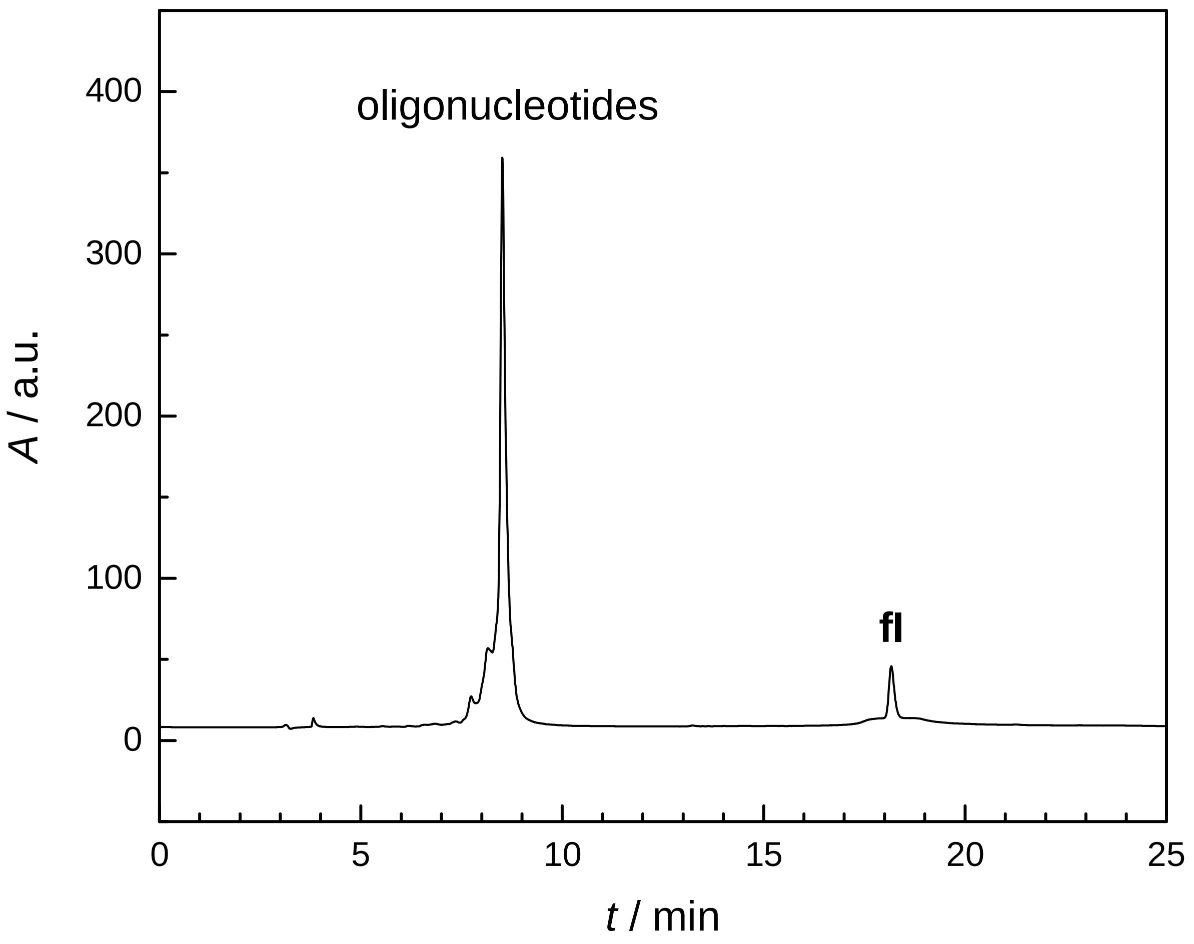

Figure S37. HPLC trace of a mixture of **fl** and ON1s; Thermo Scientific ODS Hypersil column (250 × 4.6 mm, 5 μm); flow rate = 1.0 mL min^{-1}; linear gradient (5—29% over 15 min, followed by 29—40% over 5 min) of MeCN in 50 mM aqueous triethylammonium acetate buffer (pH = 7.0); λ = 300 nm.

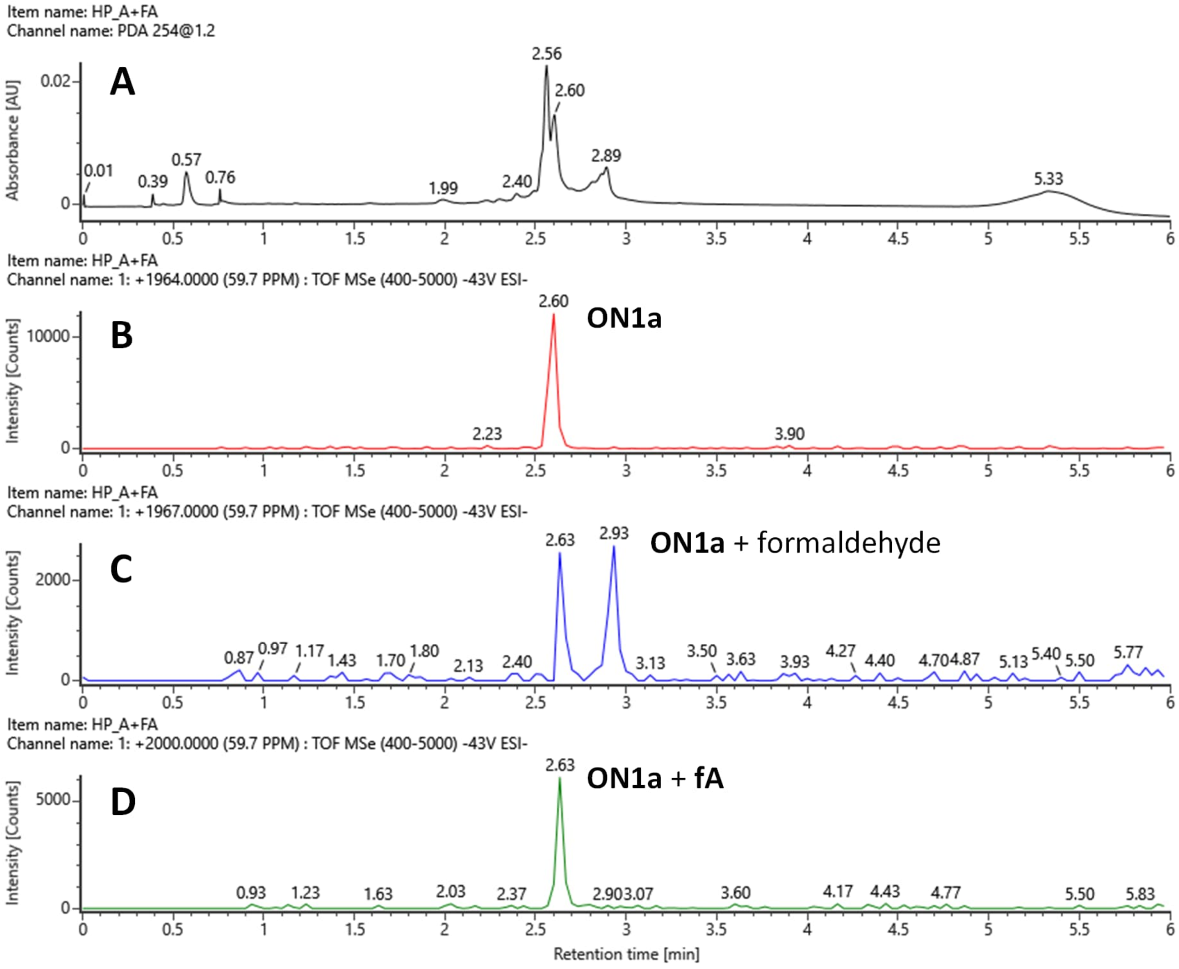

Figure S38. UV (A) and extracted ion (B—D) UPLC traces of a 50 µM mixture of hairpin oligonucleotide ON1a and fA; ACQUITY Premier OST column (50 × 2.1 mm, 1.7 µm); flow rate = 0.4 mL min-1; linear gradient (5—25% over 4 min) of MeOH in an aqueous solution of hexafluoroisopropanol (40 mM) and triethylamine (7 mM); λ = 254 nm; T = 60 °C.

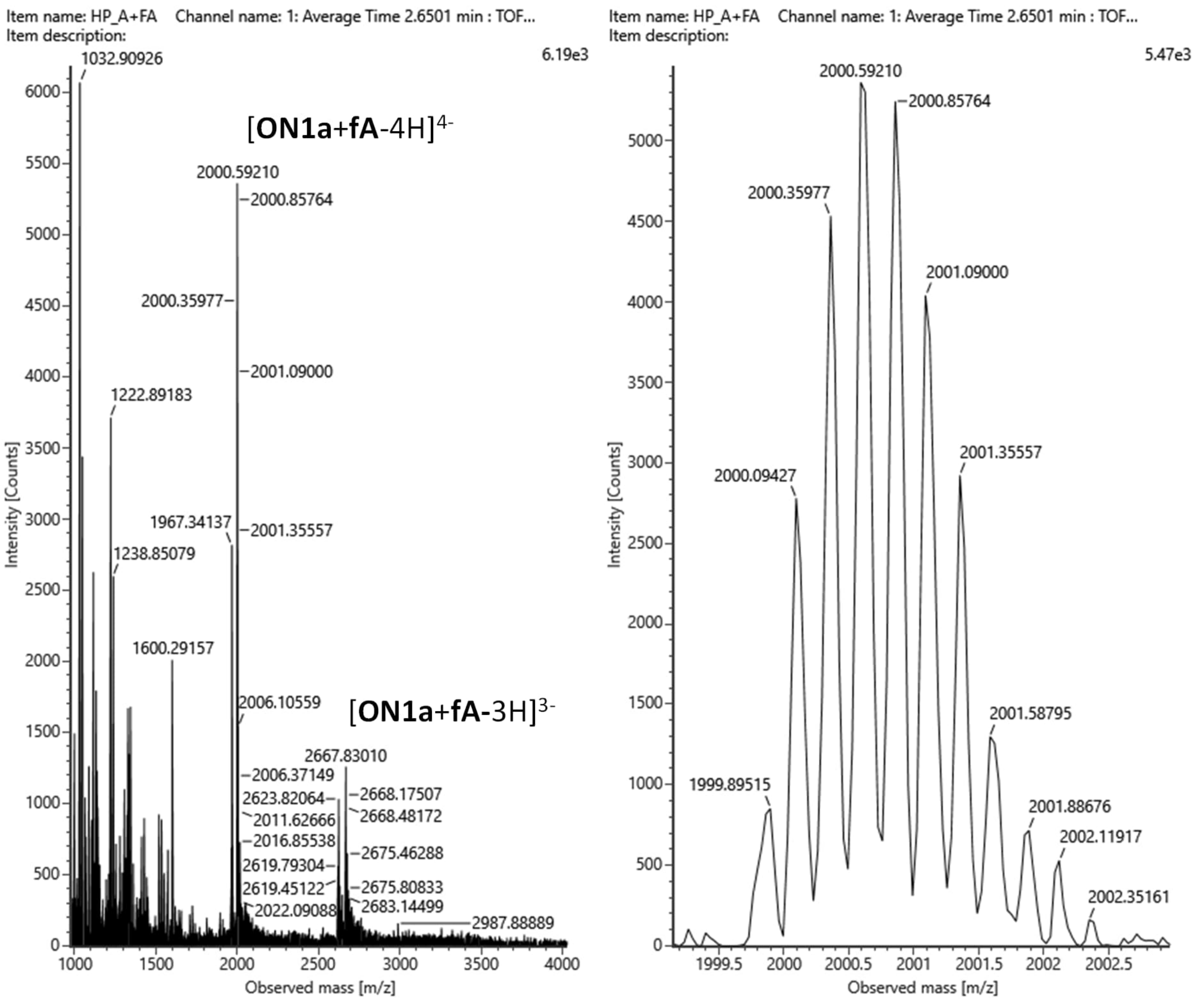

Figure S39. Mass spectrum of the covalent conjugate of hairpin oligonucleotide ON1a and fA.

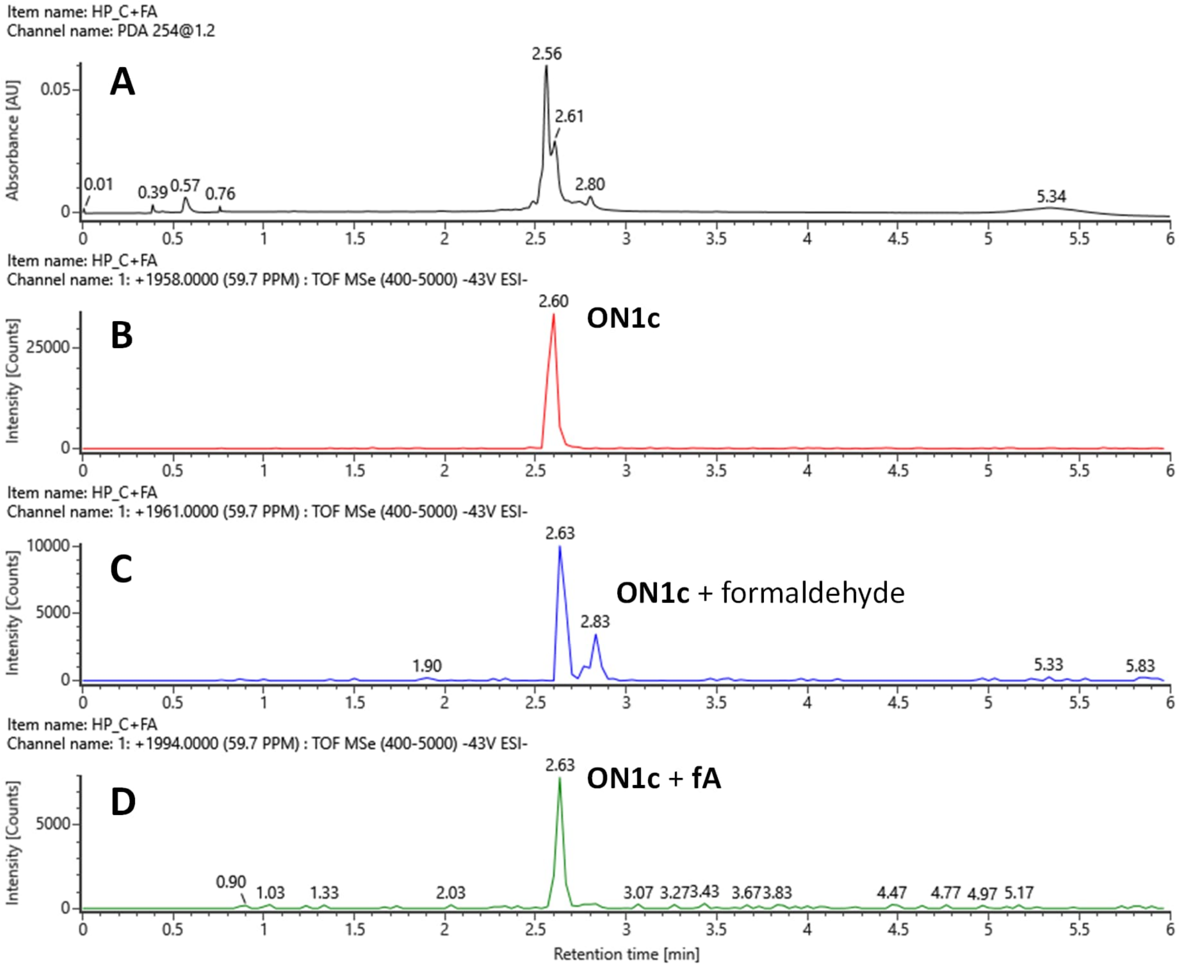

Figure S40. UV (A) and extracted ion (B—D) UPLC traces of a 50 µM mixture of hairpin oligonucleotide ON1c and fA; ACQUITY Premier OST column (50 × 2.1 mm, 1.7 µm); flow rate = 0.4 mL min-1; linear gradient (5—25% over 4 min) of MeOH in an aqueous solution of hexafluoroisopropanol (40 mM) and triethylamine (7 mM); λ = 254 nm; T = 60 °C.

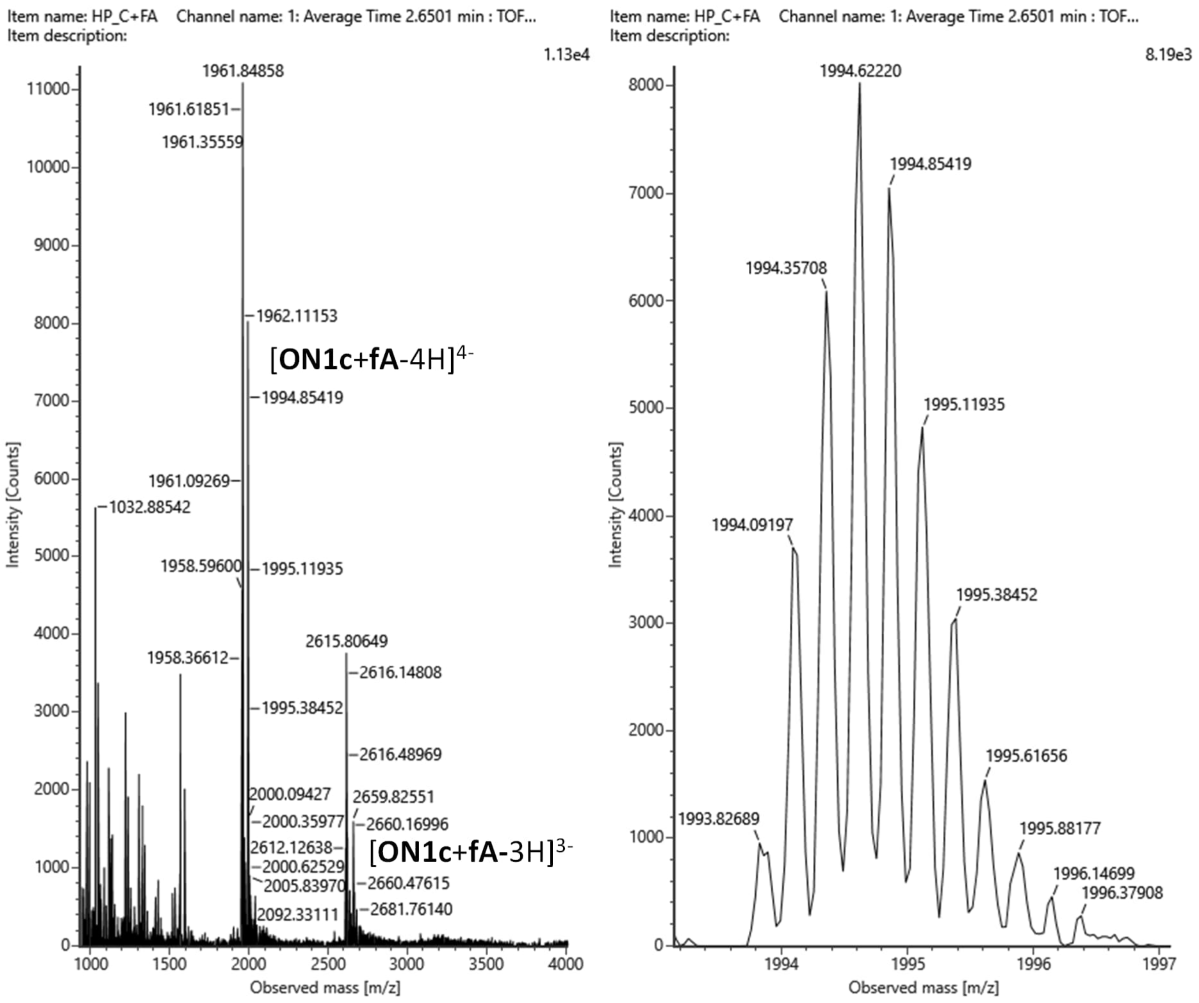

Figure S41. Mass spectrum of the covalent conjugate of hairpin oligonucleotide ON1c and fA.

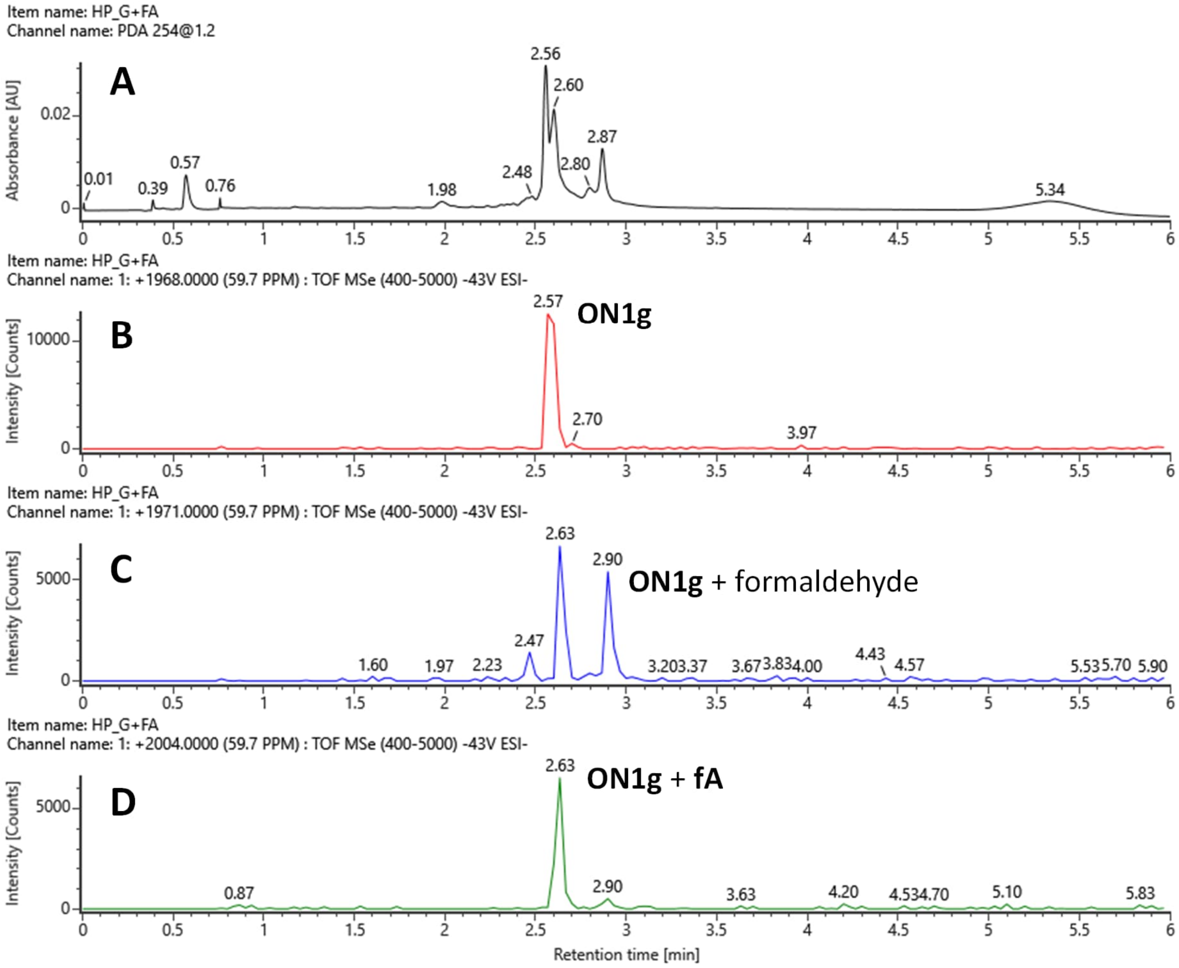

Figure S42. UV (A) and extracted ion (B—D) UPLC traces of a 50 µM mixture of hairpin oligonucleotide ON1g and fA; ACQUITY Premier OST column (50 × 2.1 mm, 1.7 µm); flow rate = 0.4 mL min-1; linear gradient (5—25% over 4 min) of MeOH in an aqueous solution of hexafluoroisopropanol (40 mM) and triethylamine (7 mM); λ = 254 nm; T = 60 °C.

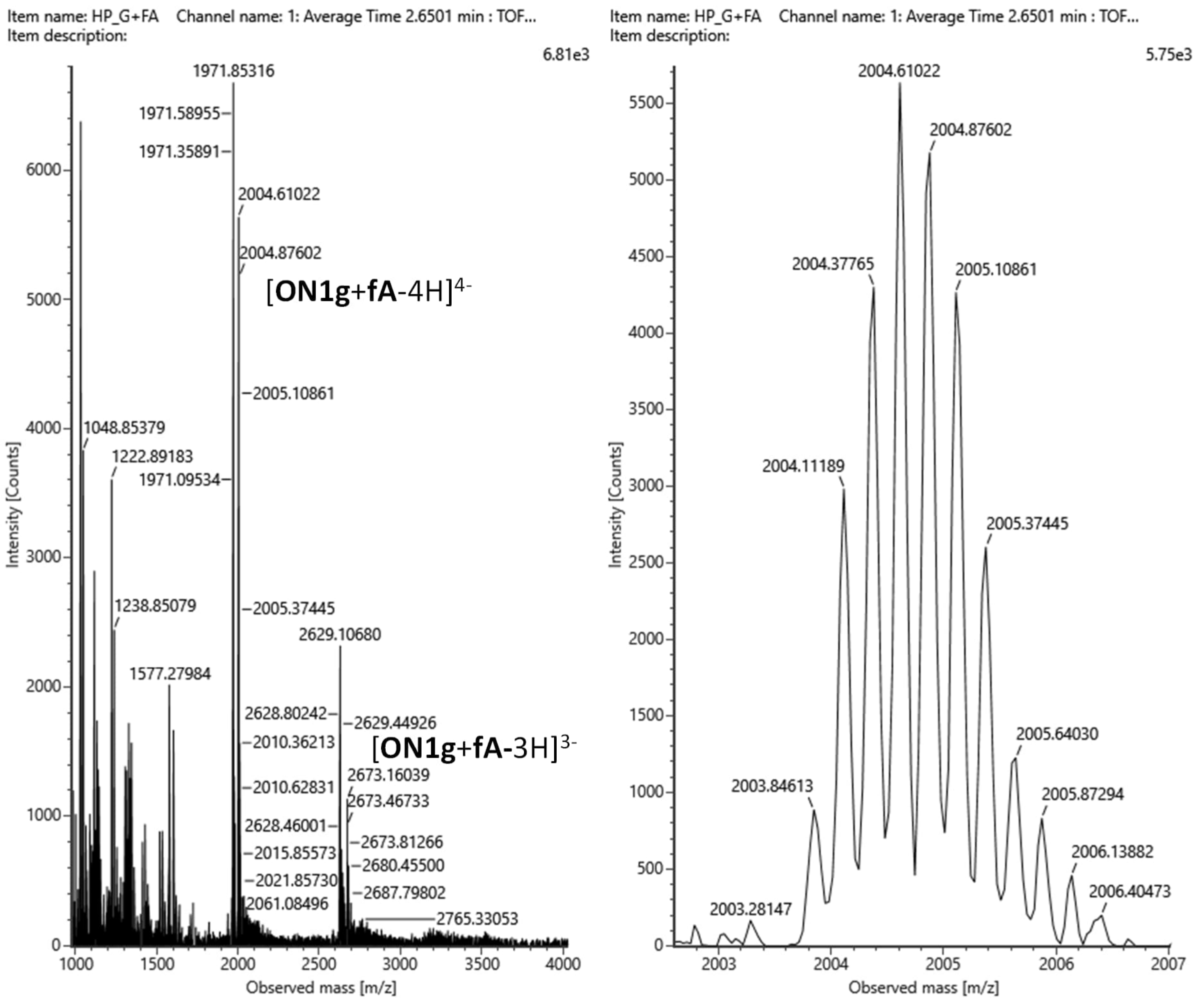

Figure S43. Mass spectrum of the covalent conjugate of hairpin oligonucleotide ON1g and fA.

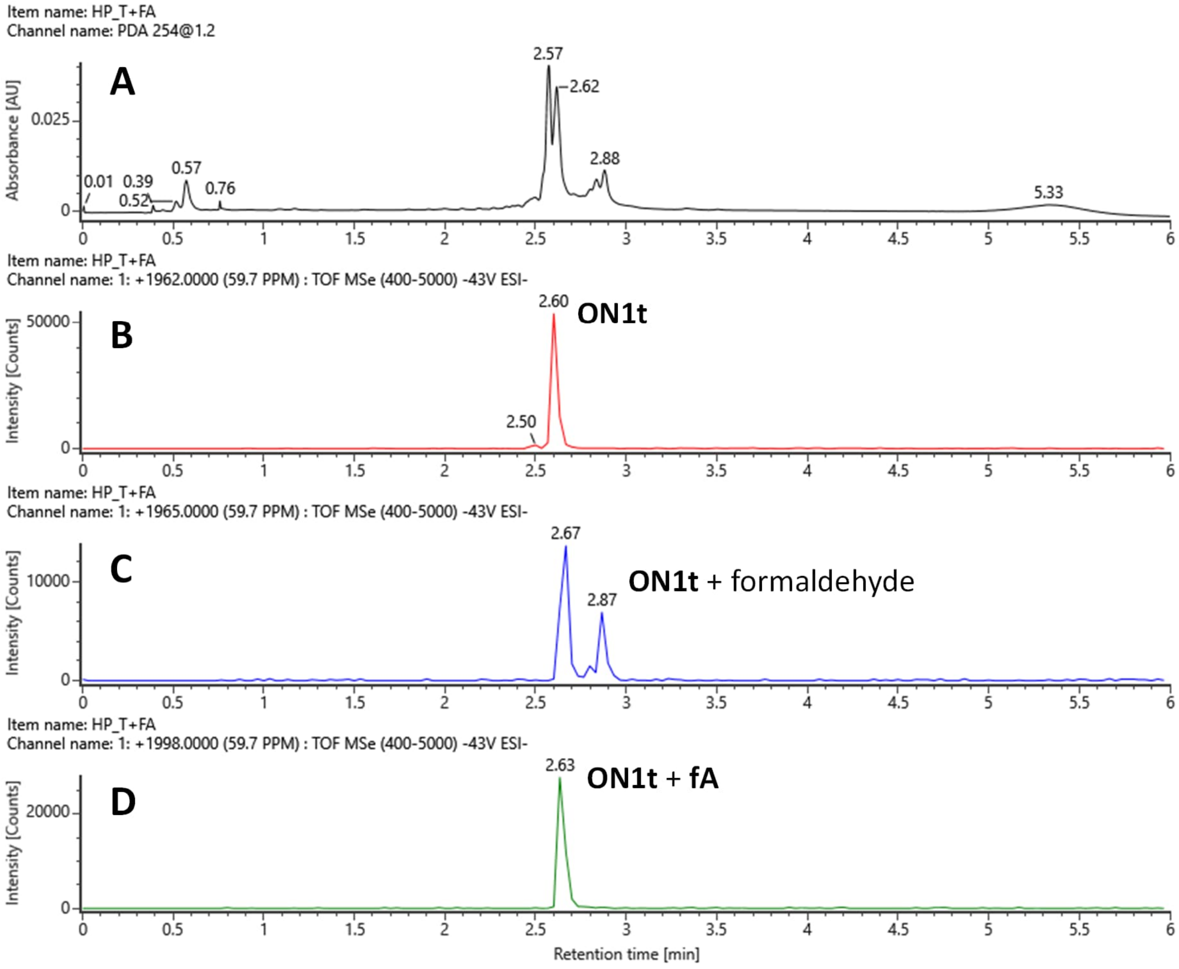

Figure S44. UV (A) and extracted ion (B—D) UPLC traces of a 50 µM mixture of hairpin oligonucleotide ON1t and fA; ACQUITY Premier OST column (50 × 2.1 mm, 1.7 µm); flow rate = 0.4 mL min-1; linear gradient (5—25% over 4 min) of MeOH in an aqueous solution of hexafluoroisopropanol (40 mM) and triethylamine (7 mM); λ = 254 nm; T = 60 °C.

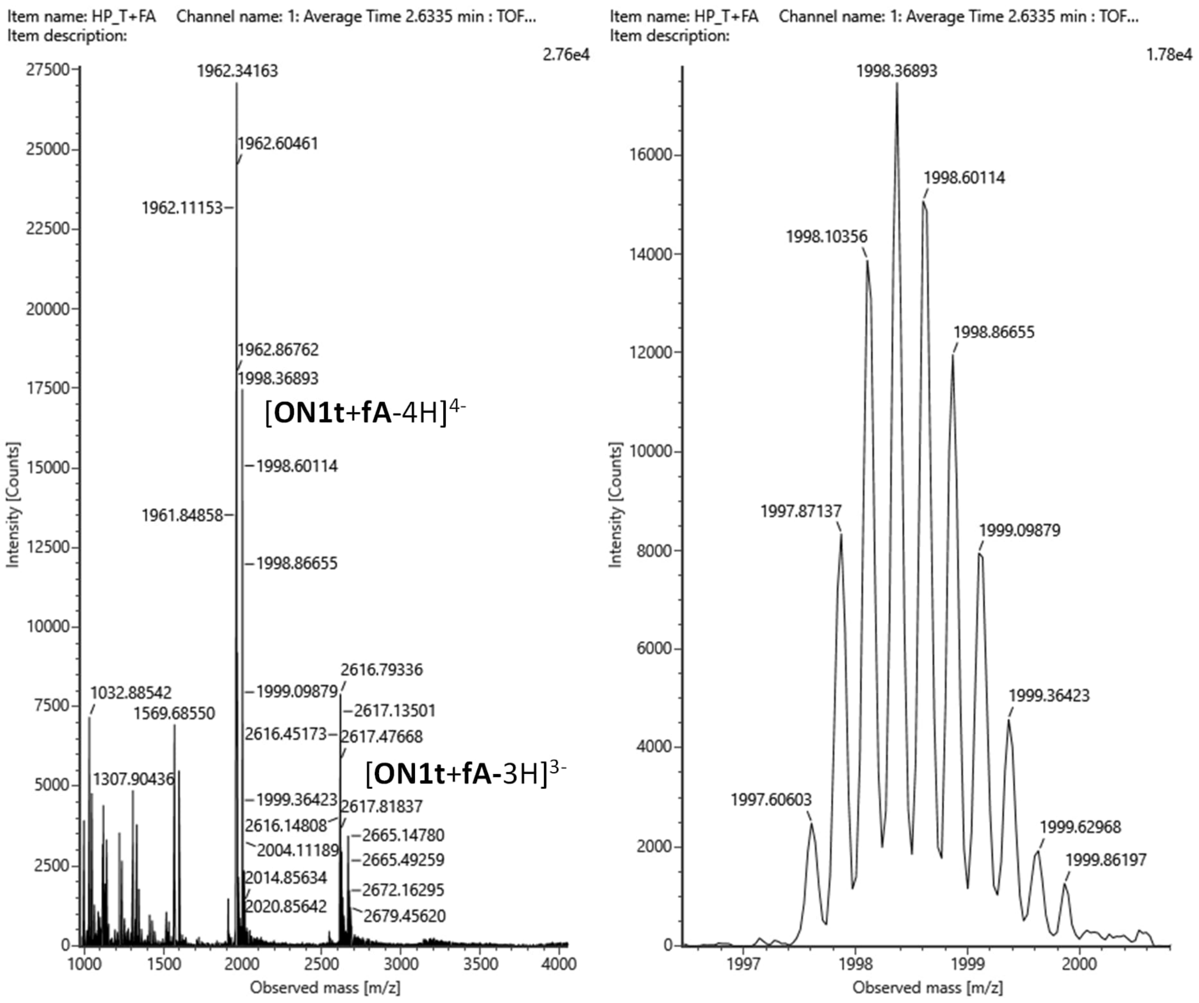

Figure S45. Mass spectrum of the covalent conjugate of hairpin oligonucleotide ON1t and fA.

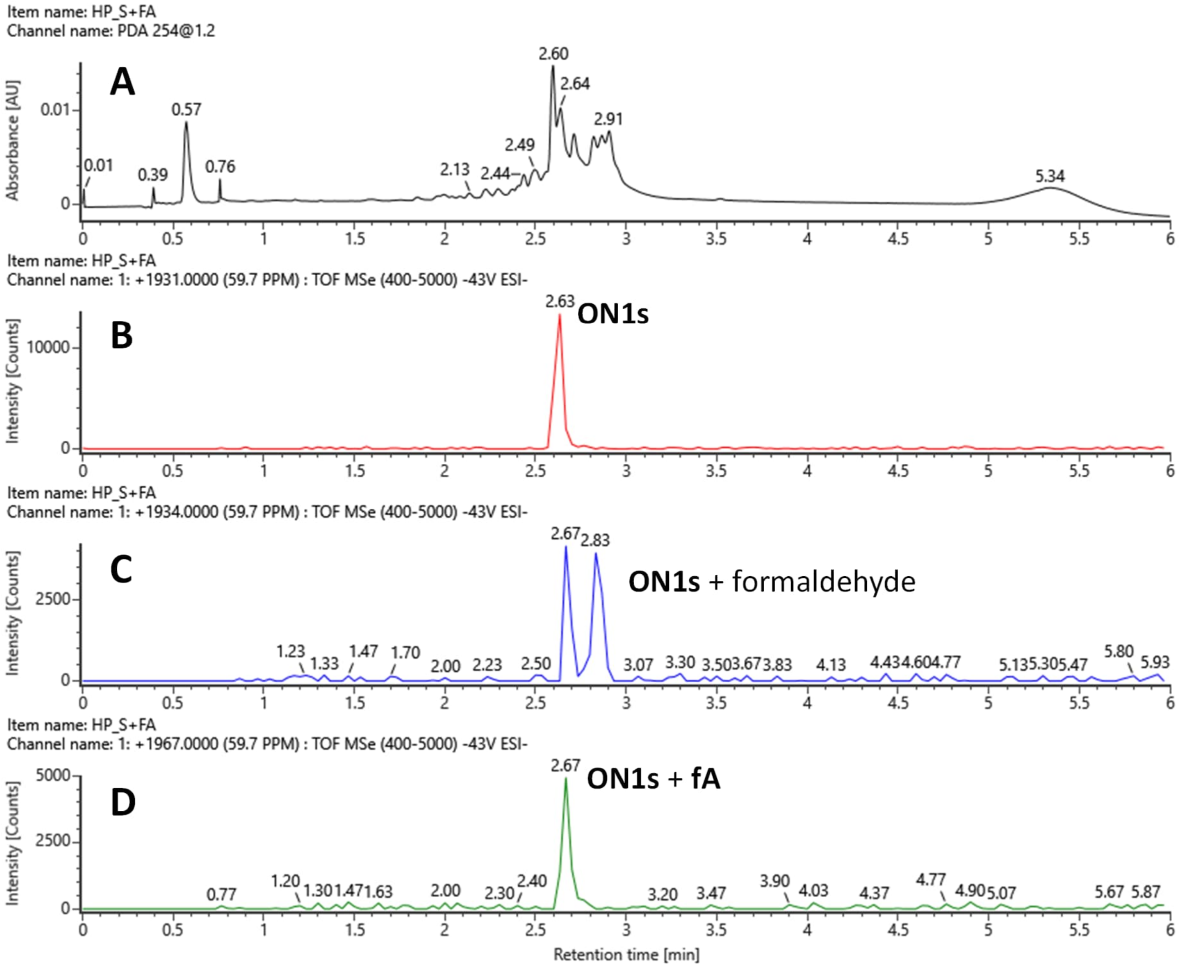

Figure S46. UV (A) and extracted ion (B—D) UPLC traces of a 50 µM mixture of hairpin oligonucleotide ON1s and fA; ACQUITY Premier OST column (50 × 2.1 mm, 1.7 µm); flow rate = 0.4 mL min-1; linear gradient (5—25% over 4 min) of MeOH in an aqueous solution of hexafluoroisopropanol (40 mM) and triethylamine (7 mM); λ = 254 nm; T = 60 °C.

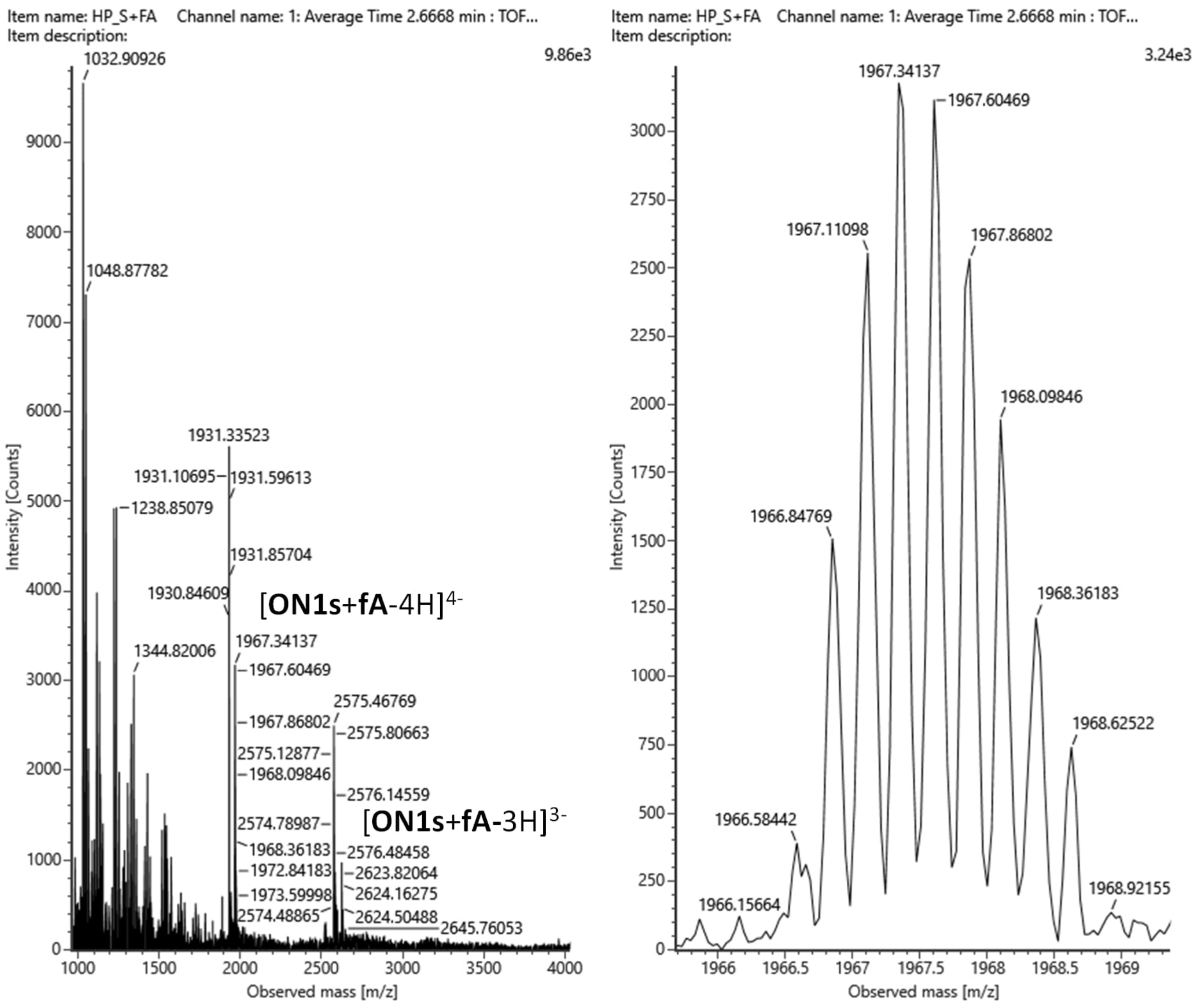

Figure S47. Mass spectrum of the covalent conjugate of hairpin oligonucleotide ON1s and fA.

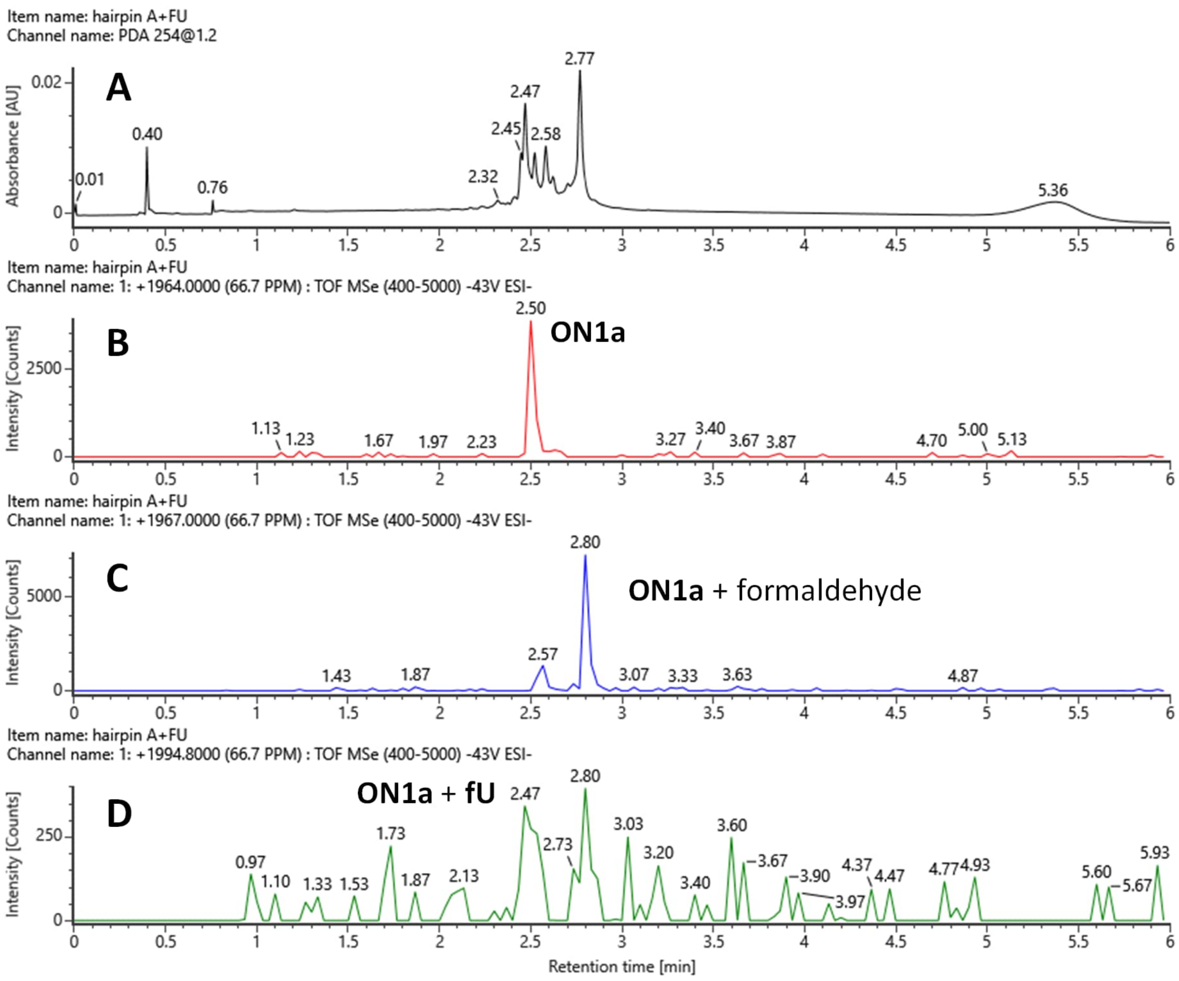

Figure S48. UV (A) and extracted ion (B—D) UPLC traces of a 50 µM mixture of hairpin oligonucleotide ON1a and fU; ACQUITY Premier OST column (50 × 2.1 mm, 1.7 µm); flow rate = 0.4 mL min-1; linear gradient (5—25% over 4 min) of MeOH in an aqueous solution of hexafluoroisopropanol (40 mM) and triethylamine (7 mM); λ = 254 nm; T = 60 °C.

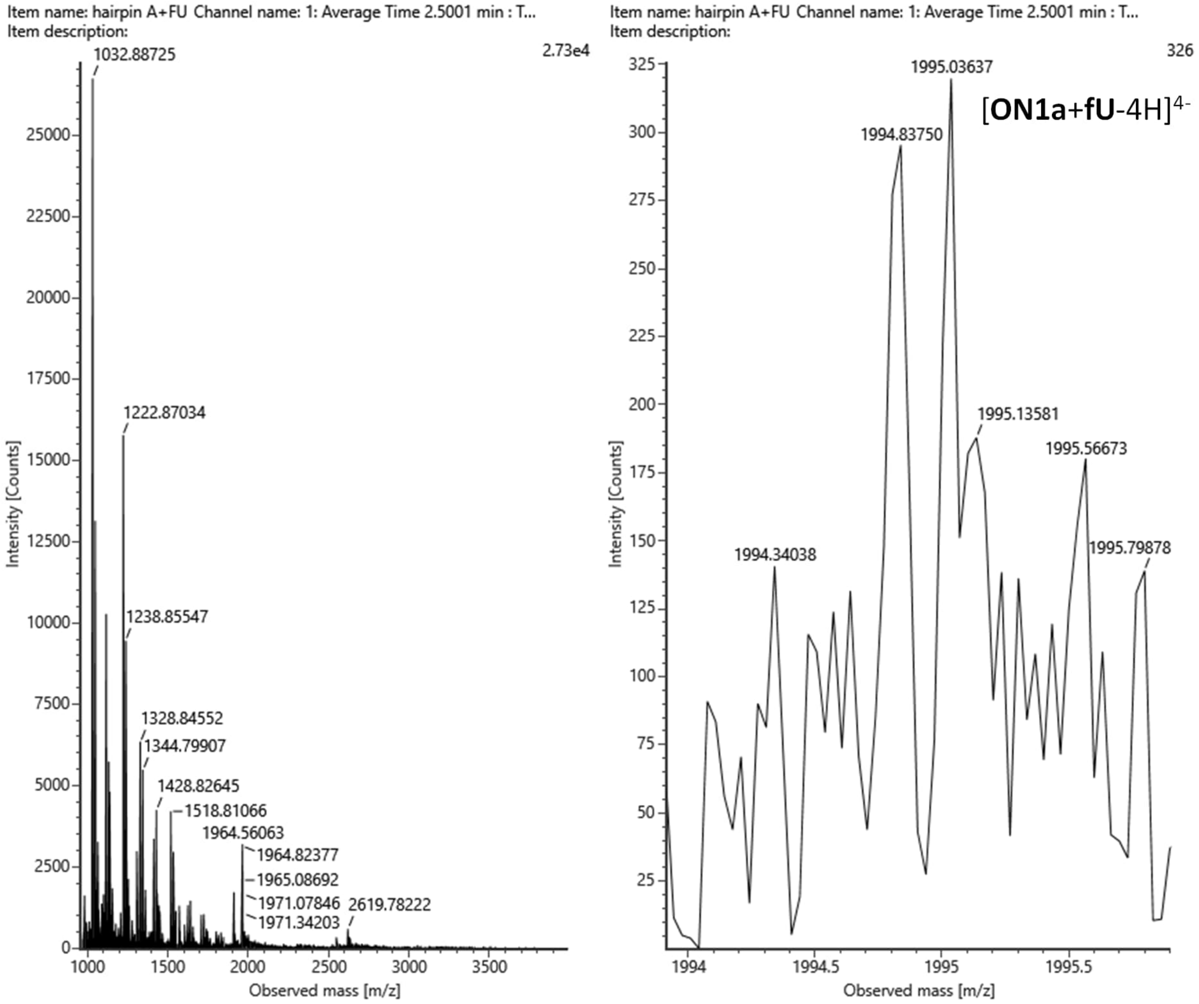

Figure S49. Mass spectrum of the covalent conjugate of hairpin oligonucleotide ON1a and fU.

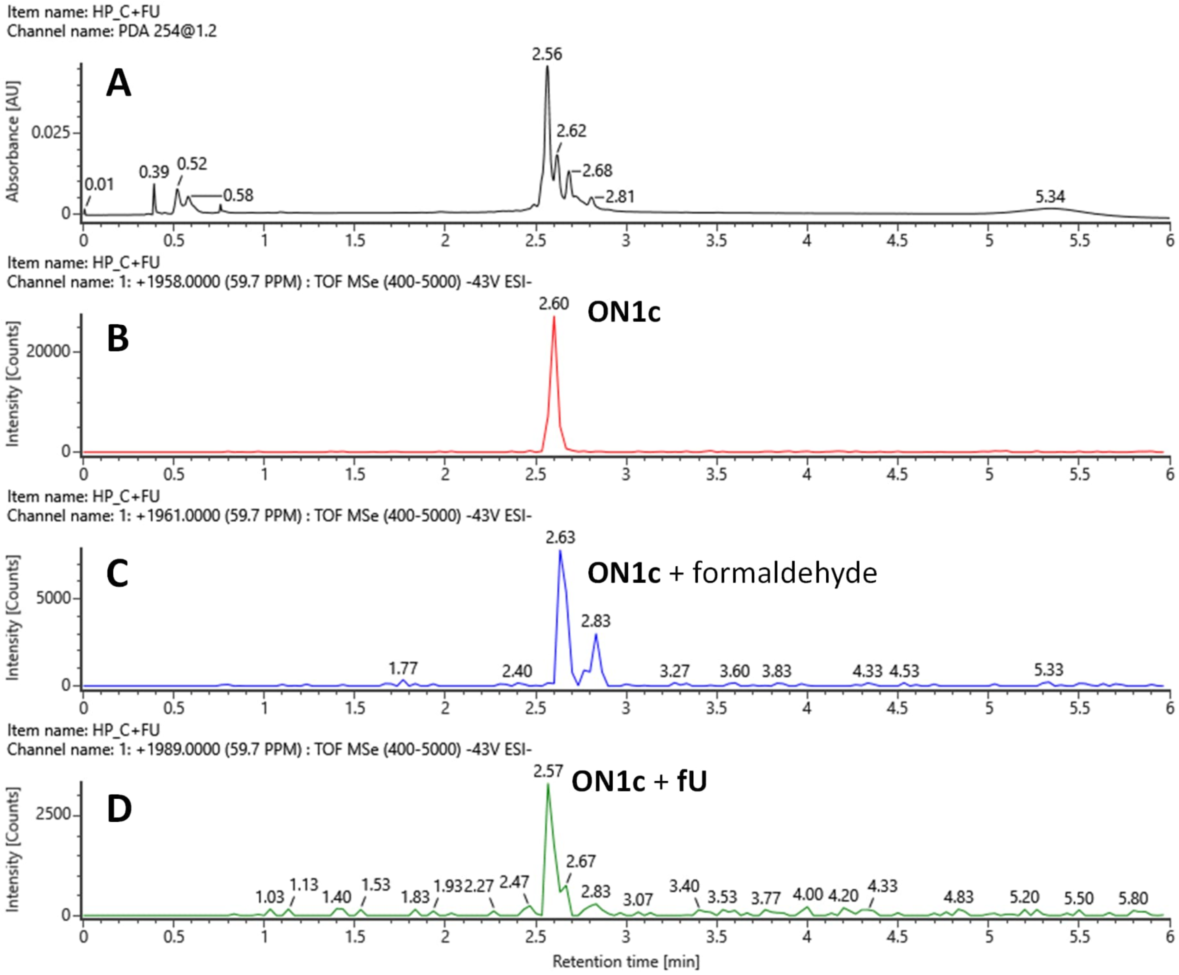

Figure S50. UV (A) and extracted ion (B—D) UPLC traces of a 50 µM mixture of hairpin oligonucleotide ON1c and fU; ACQUITY Premier OST column (50 × 2.1 mm, 1.7 µm); flow rate = 0.4 mL min-1; linear gradient (5—25% over 4 min) of MeOH in an aqueous solution of hexafluoroisopropanol (40 mM) and triethylamine (7 mM); λ = 254 nm; T = 60 °C.

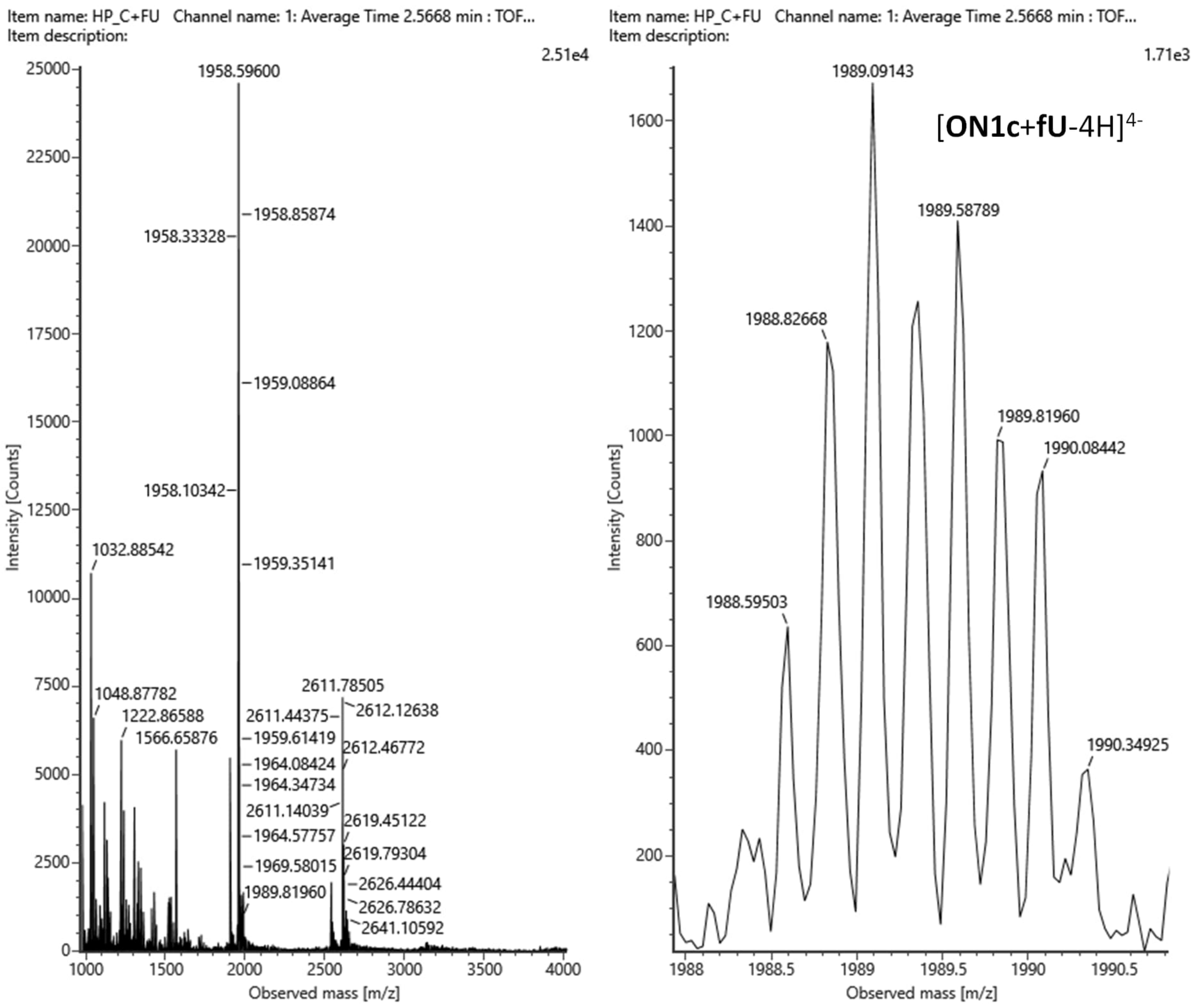

Figure S51. Mass spectrum of the covalent conjugate of hairpin oligonucleotide ON1c and fU.

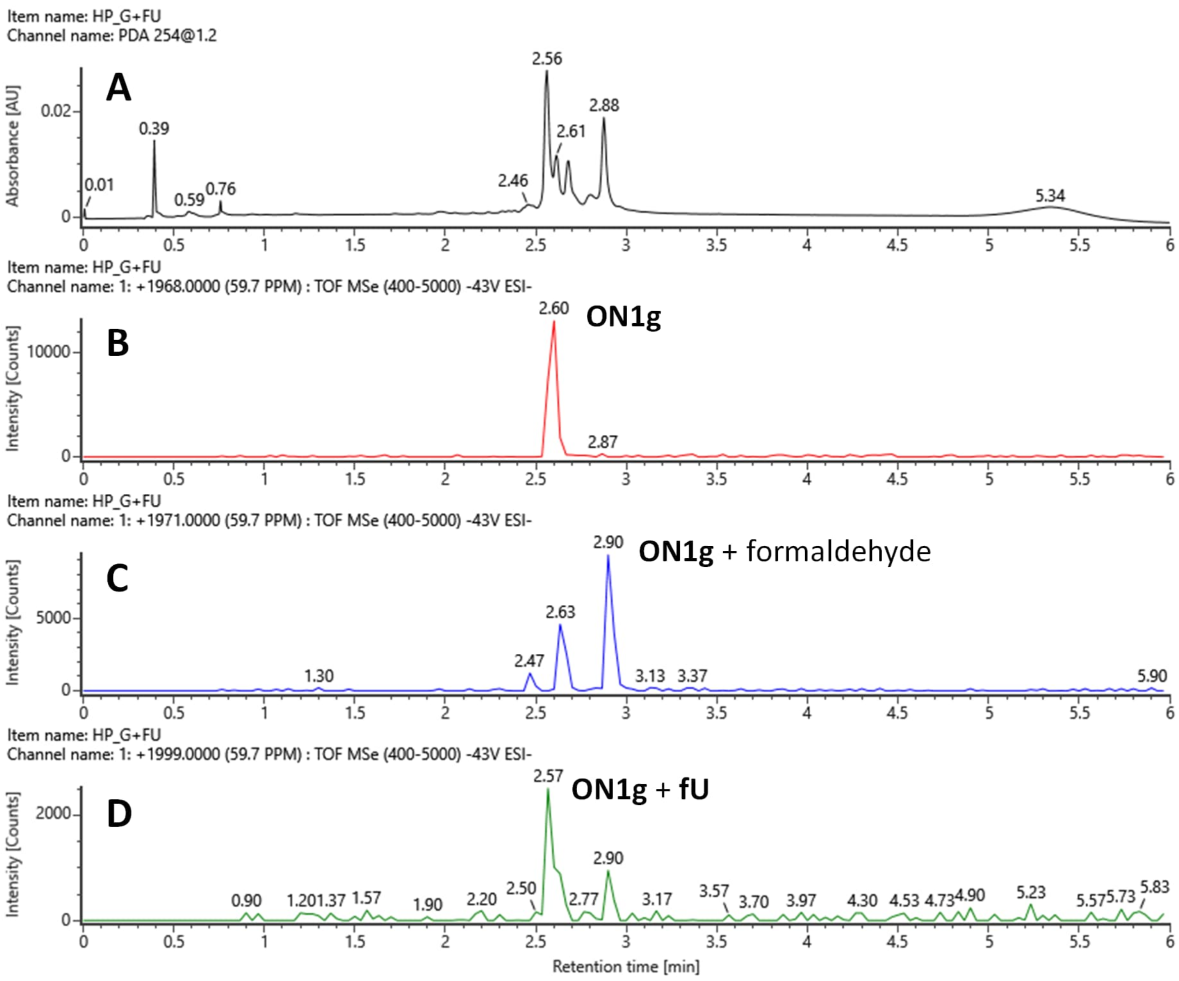

Figure S52. UV (A) and extracted ion (B—D) UPLC traces of a 50 µM mixture of hairpin oligonucleotide ON1g and fU; ACQUITY Premier OST column (50 × 2.1 mm, 1.7 µm); flow rate = 0.4 mL min-1; linear gradient (5—25% over 4 min) of MeOH in an aqueous solution of hexafluoroisopropanol (40 mM) and triethylamine (7 mM); λ = 254 nm; T = 60 °C.

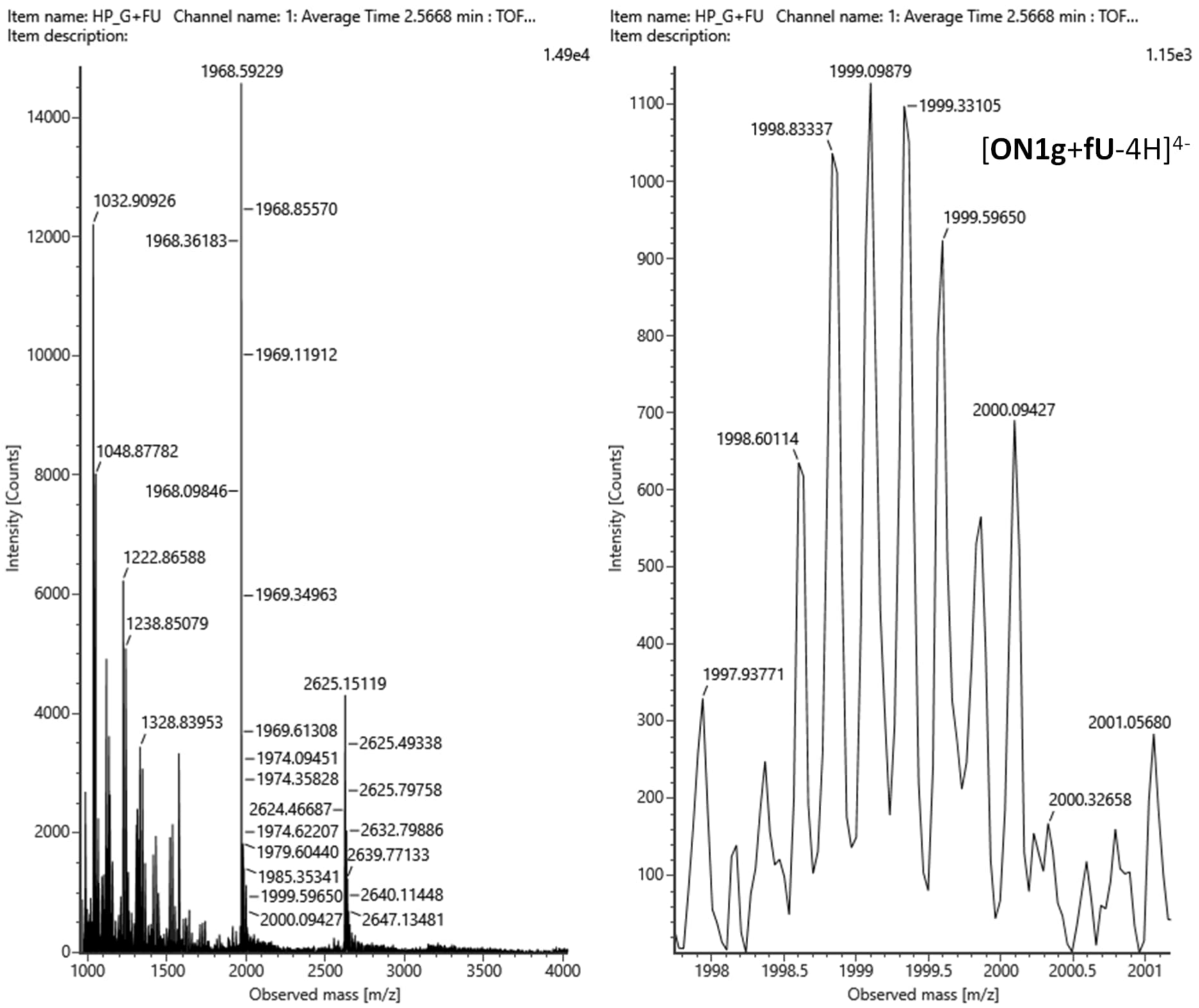

Figure S53. Mass spectrum of the covalent conjugate of hairpin oligonucleotide ON1g and fU.

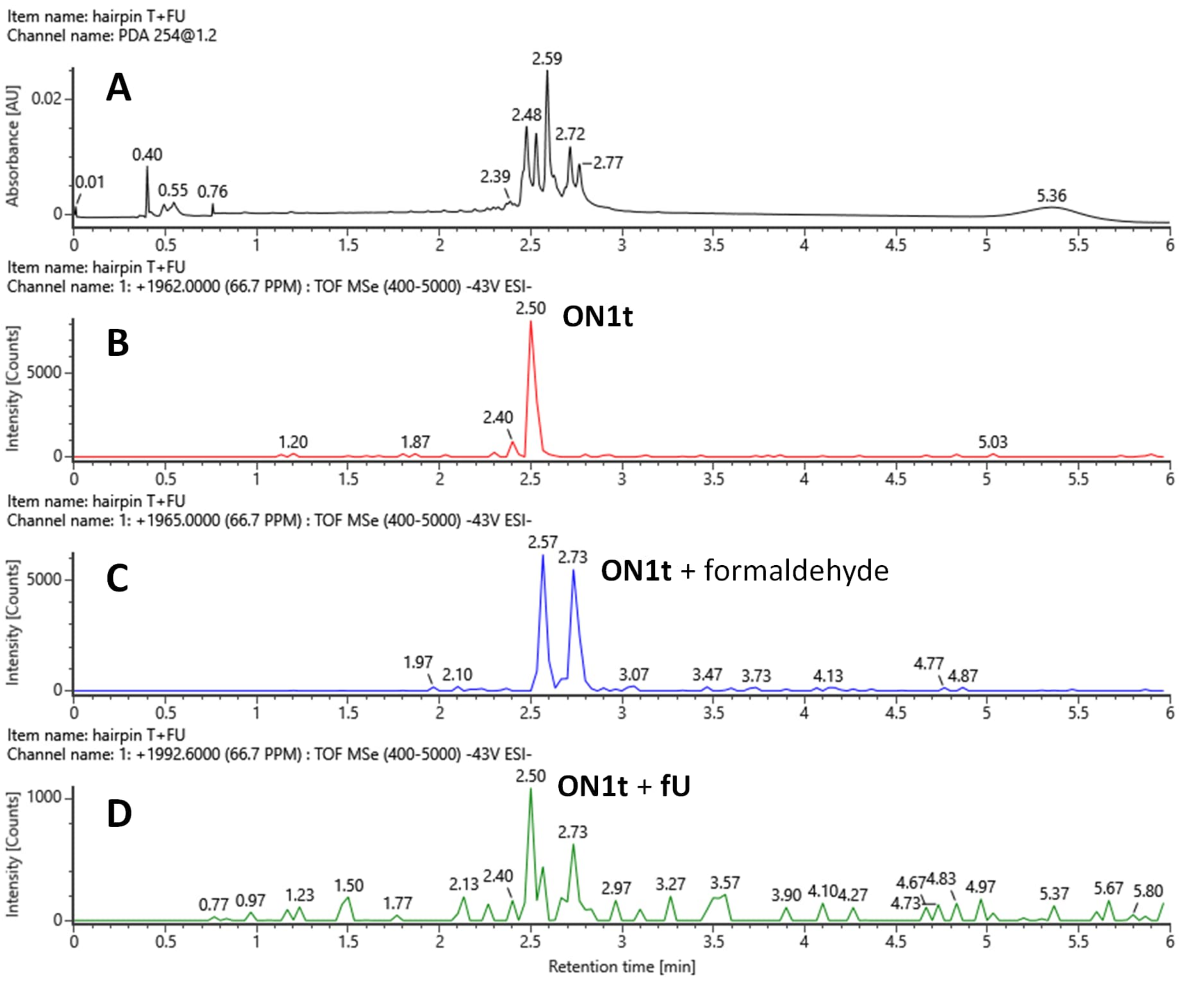

Figure S54. UV (A) and extracted ion (B—D) UPLC traces of a 50 µM mixture of hairpin oligonucleotide ON1t and fU; ACQUITY Premier OST column (50 × 2.1 mm, 1.7 µm); flow rate = 0.4 mL min-1; linear gradient (5—25% over 4 min) of MeOH in an aqueous solution of hexafluoroisopropanol (40 mM) and triethylamine (7 mM); λ = 254 nm; T = 60 °C.

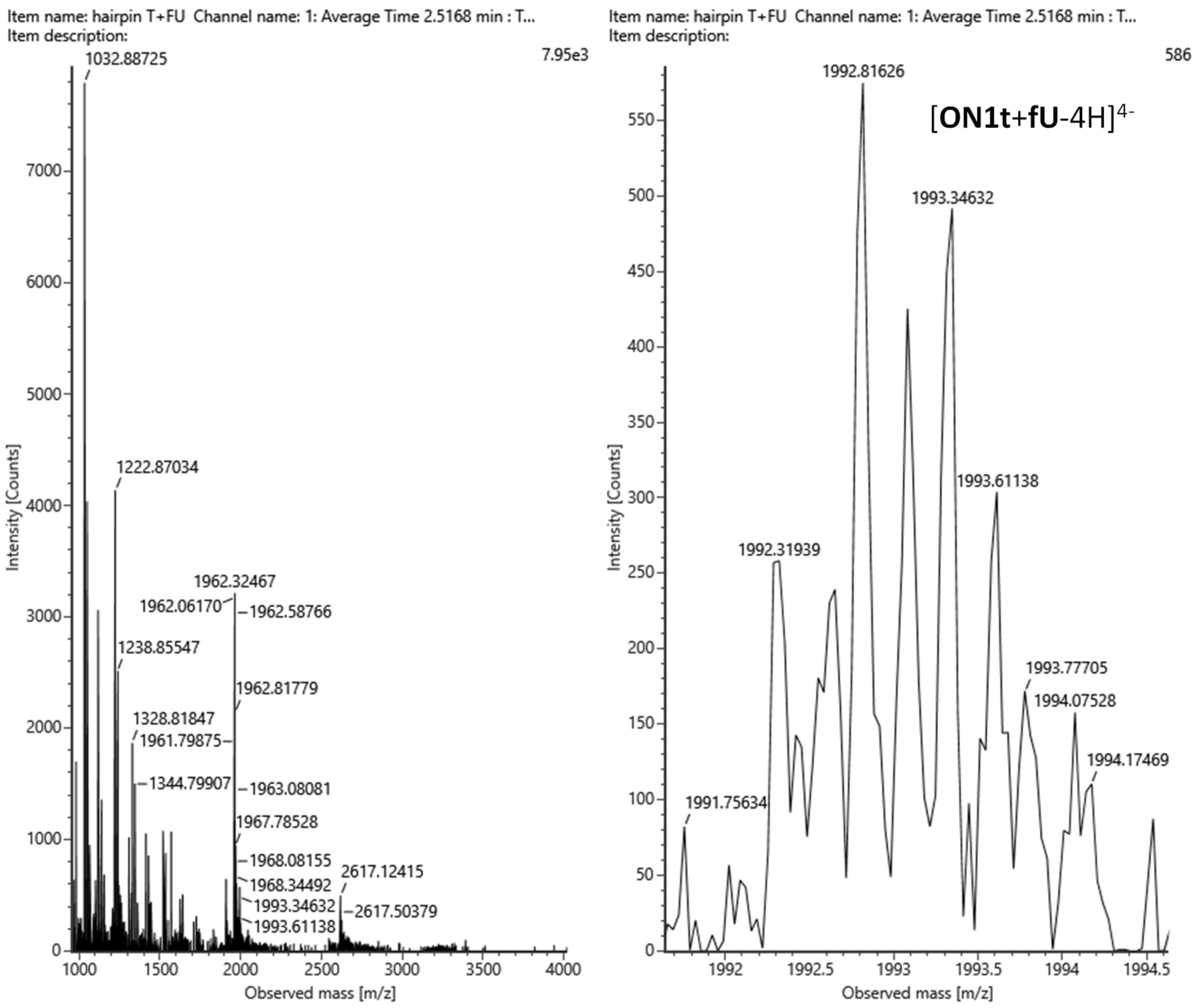

Figure S55. Mass spectrum of the covalent conjugate of hairpin oligonucleotide ON1t and fU.

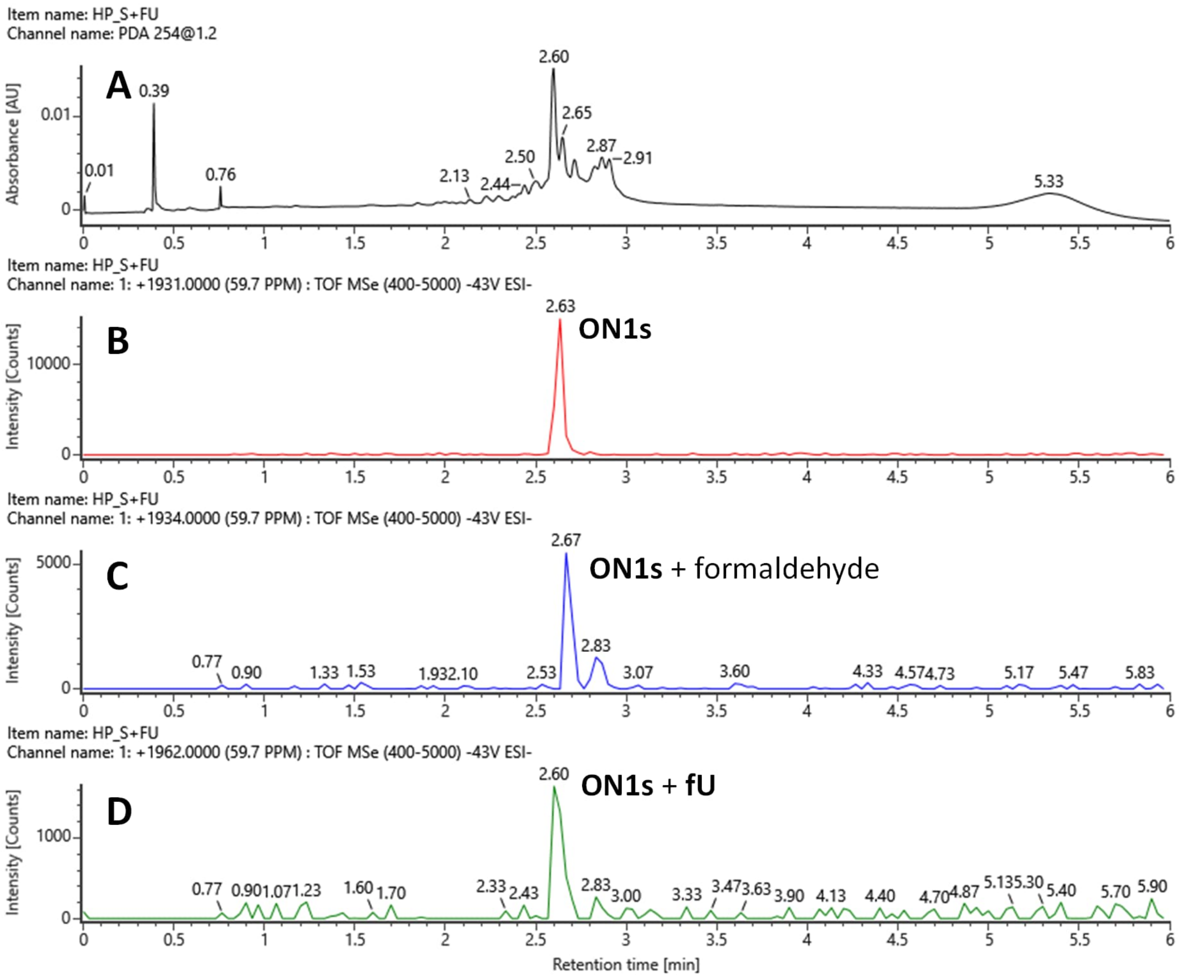

Figure S56. UV (A) and extracted ion (B—D) UPLC traces of a 50 µM mixture of hairpin oligonucleotide ON1s and fU; ACQUITY Premier OST column (50 × 2.1 mm, 1.7 µm); flow rate = 0.4 mL min-1; linear gradient (5—25% over 4 min) of MeOH in an aqueous solution of hexafluoroisopropanol (40 mM) and triethylamine (7 mM); λ = 254 nm; T = 60 °C.

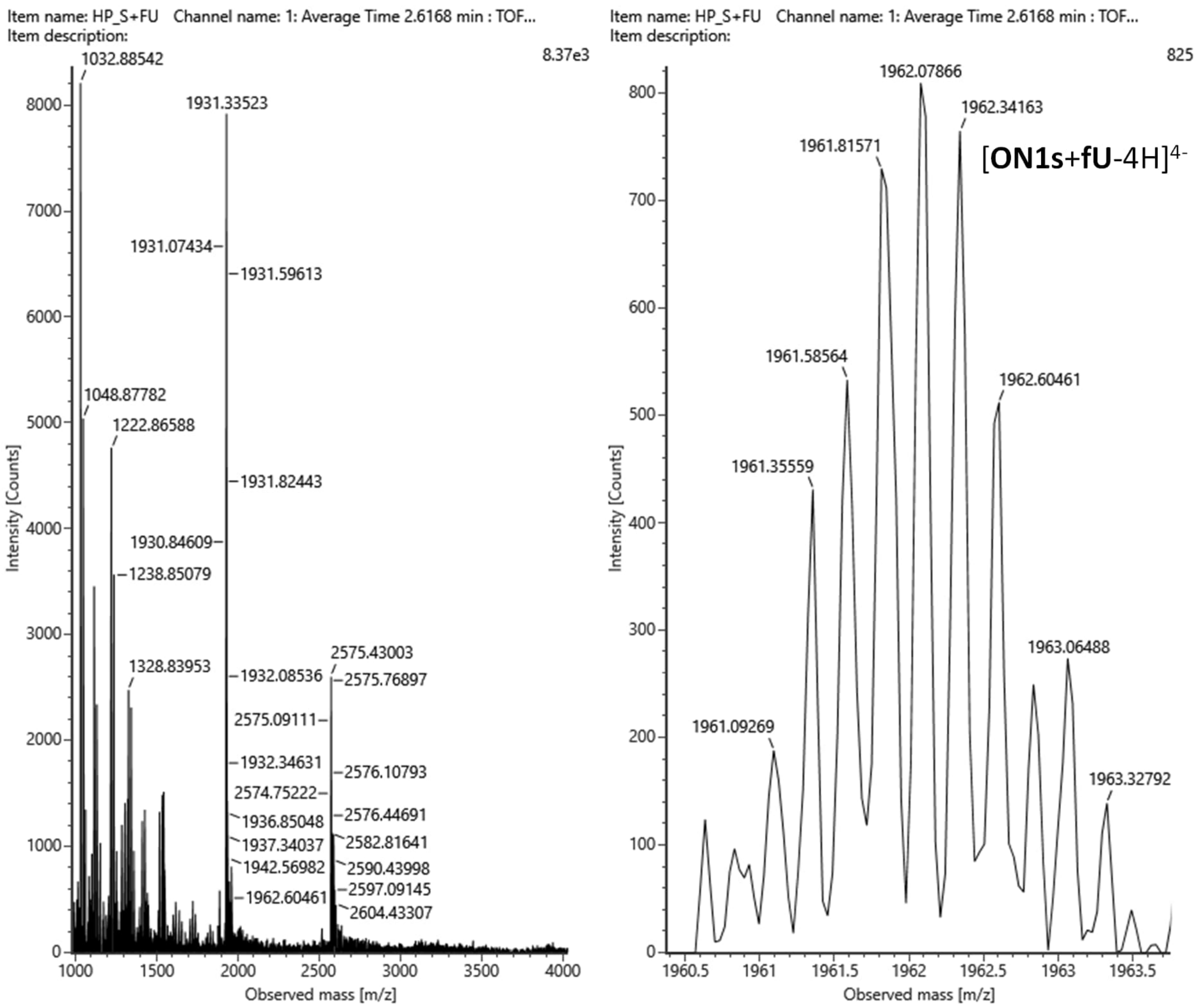

Figure S57. Mass spectrum of the covalent conjugate of hairpin oligonucleotide ON1t and fU.

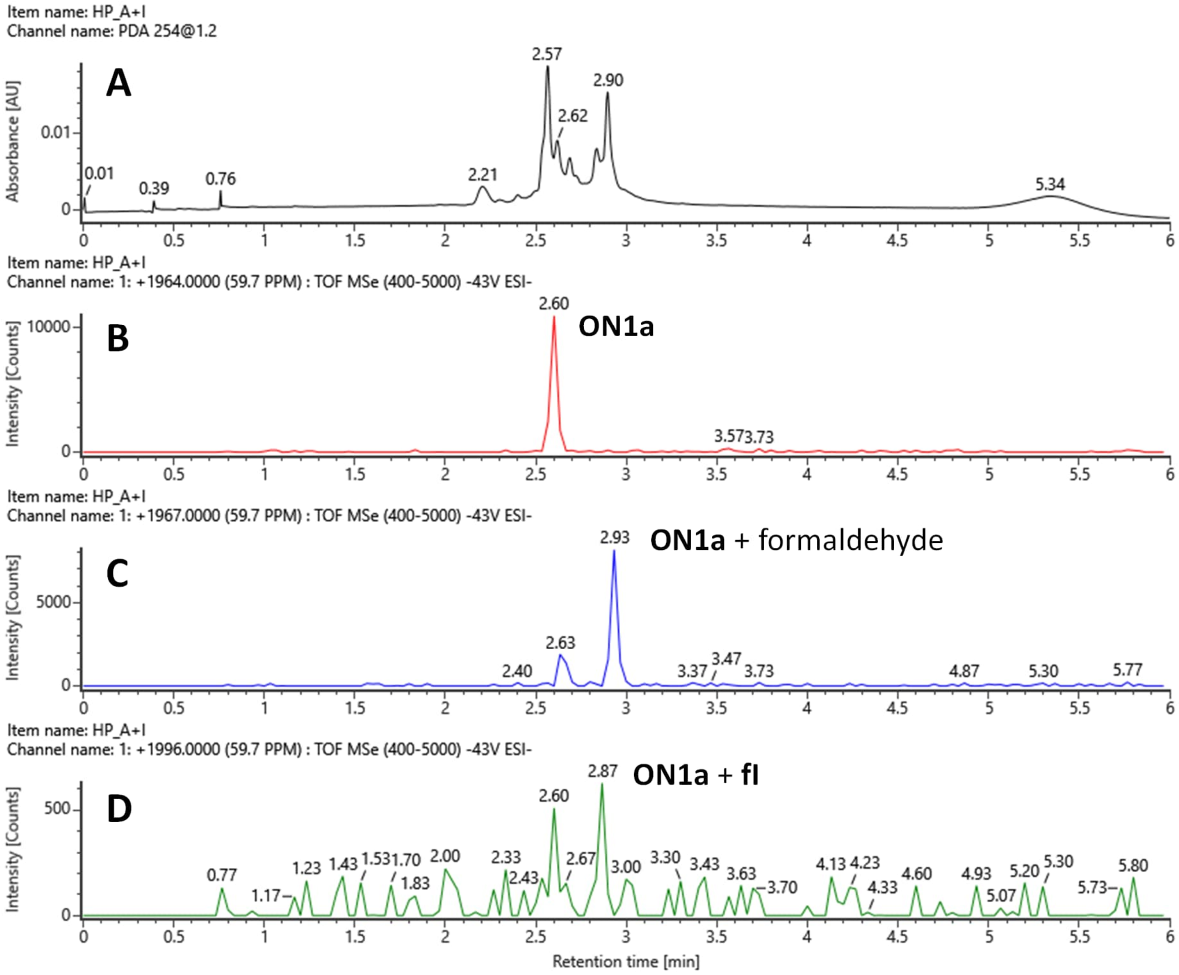

Figure S58. UV (A) and extracted ion (B—D) UPLC traces of a 50 µM mixture of hairpin oligonucleotide ON1a and fI; ACQUITY Premier OST column (50 × 2.1 mm, 1.7 µm); flow rate = 0.4 mL min-1; linear gradient (5—25% over 4 min) of MeOH in an aqueous solution of hexafluoroisopropanol (40 mM) and triethylamine (7 mM); λ = 254 nm; T = 60 °C.

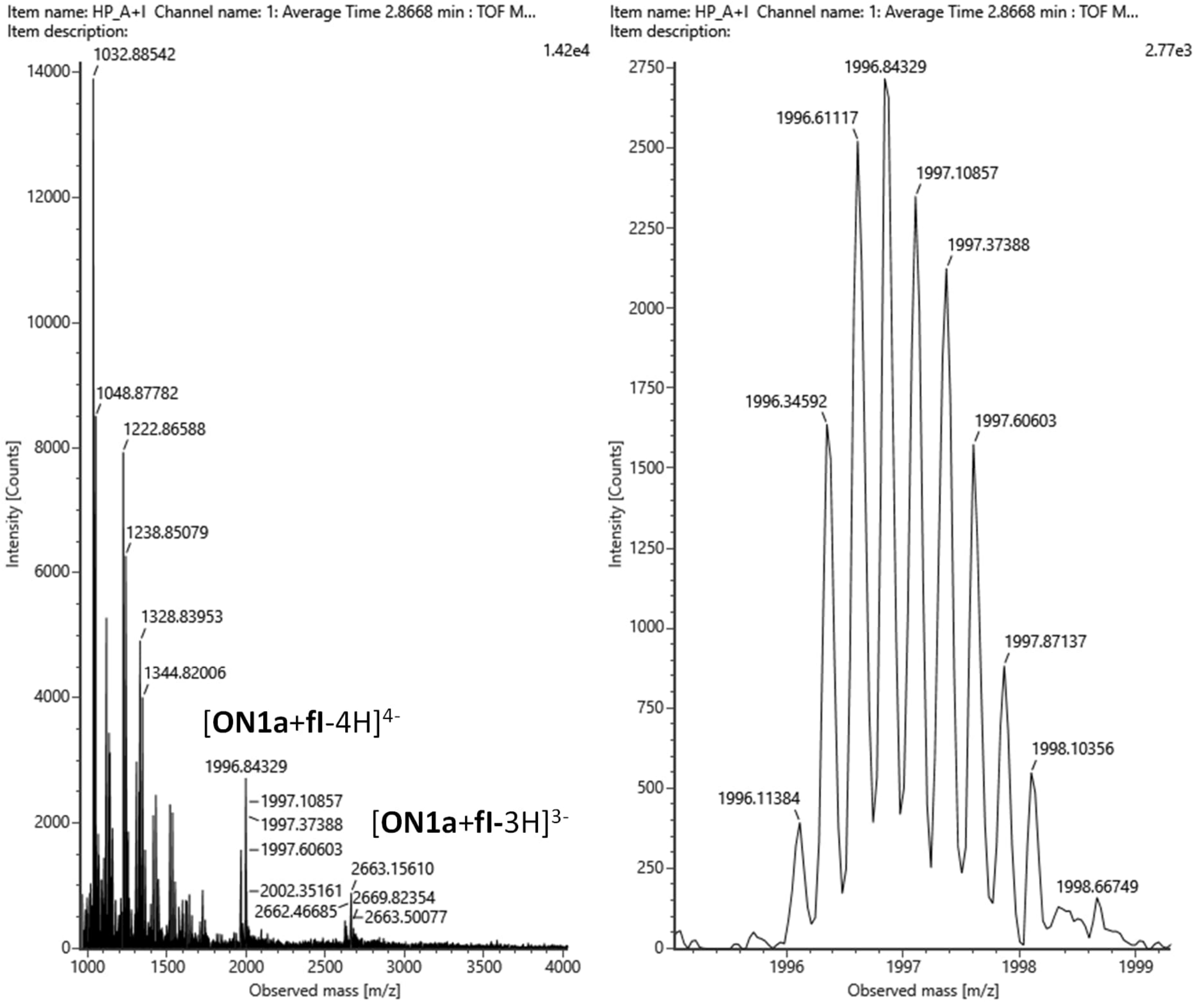

Figure S59. Mass spectrum of the covalent conjugate of hairpin oligonucleotide ON1a and fI.

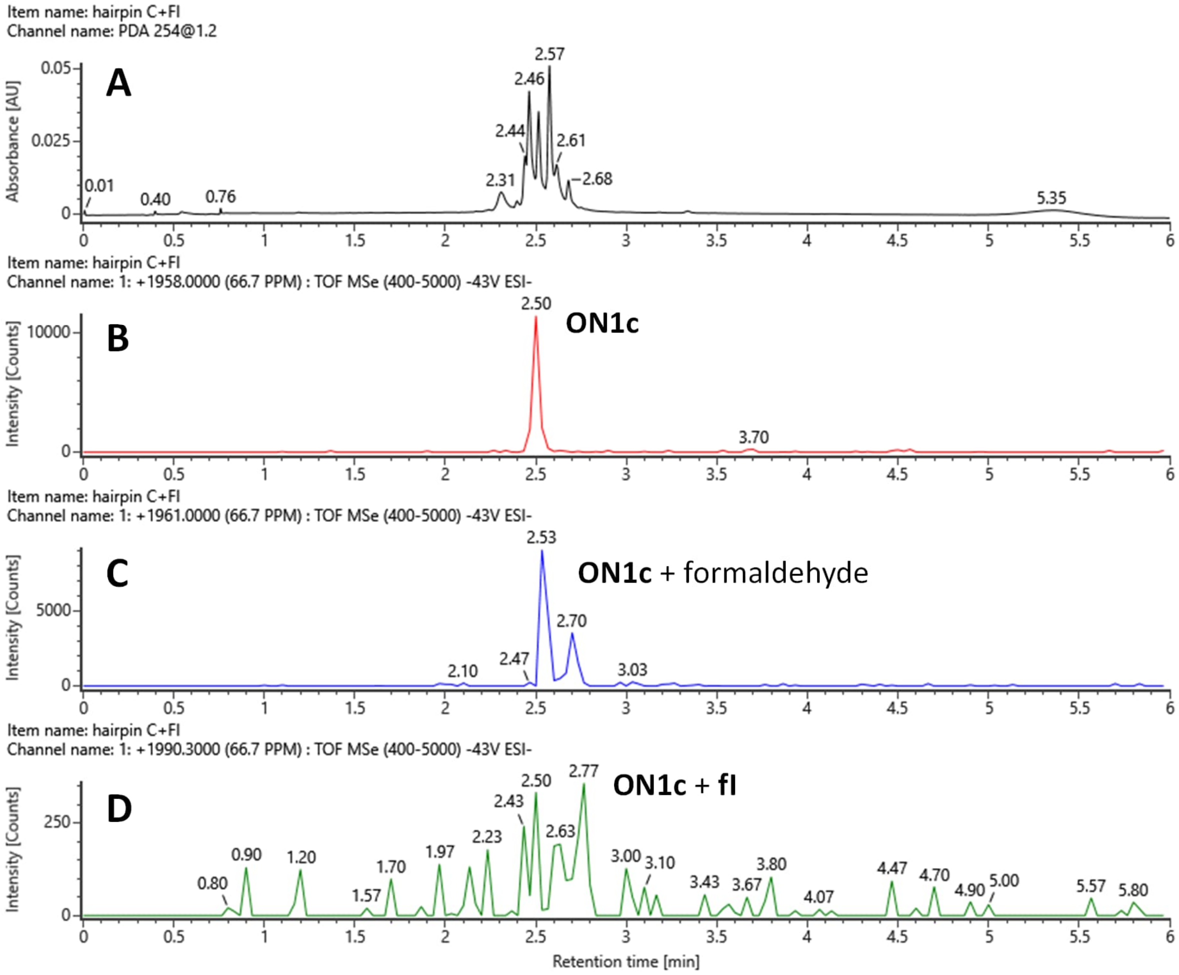

Figure S60. UV (A) and extracted ion (B—D) UPLC traces of a 50 µM mixture of hairpin oligonucleotide ON1c and fI; ACQUITY Premier OST column (50 × 2.1 mm, 1.7 µm); flow rate = 0.4 mL min-1; linear gradient (5—25% over 4 min) of MeOH in an aqueous solution of hexafluoroisopropanol (40 mM) and triethylamine (7 mM); λ = 254 nm; T = 60 °C.

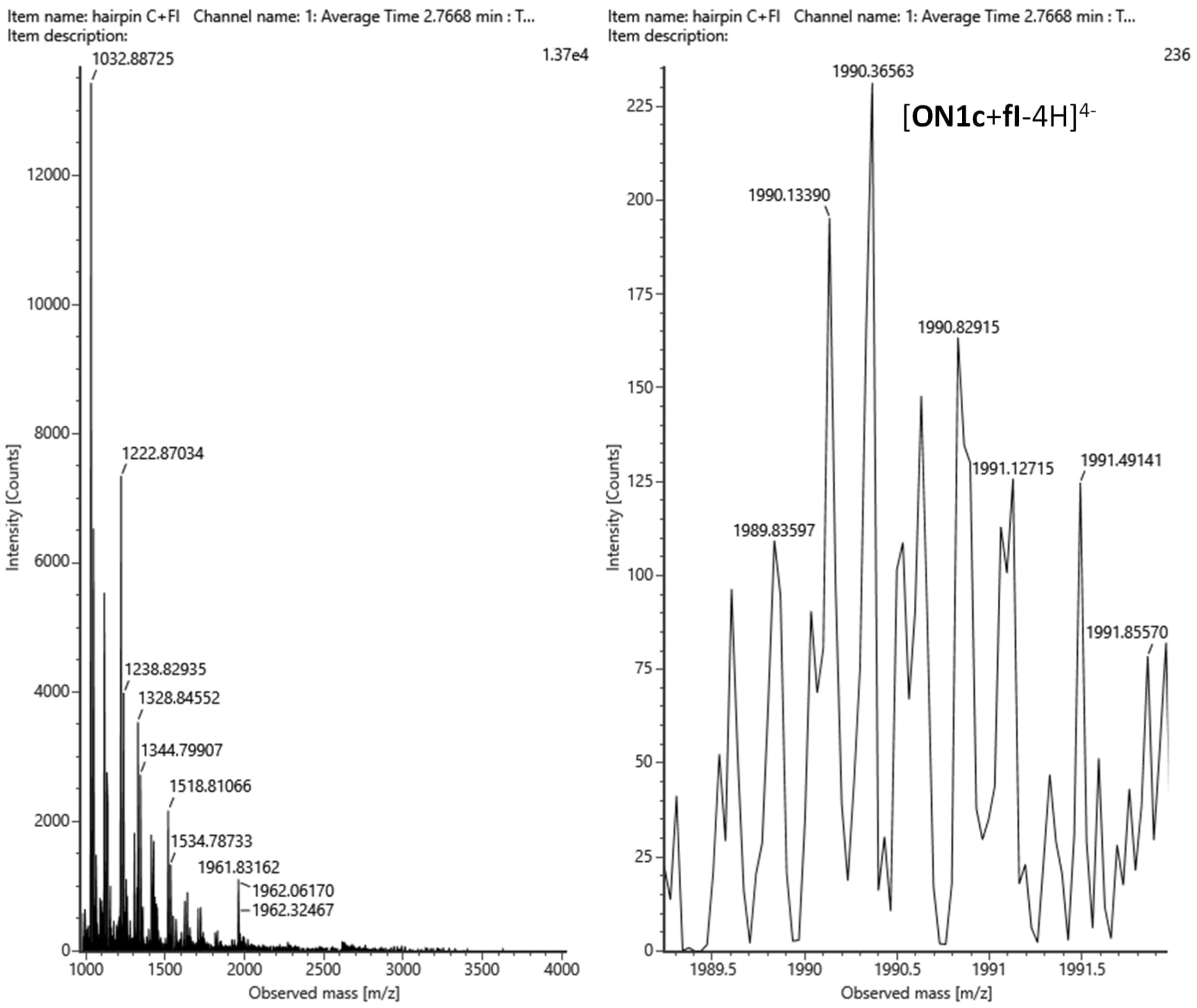

Figure S61. Mass spectrum of the covalent conjugate of hairpin oligonucleotide ON1c and fI.

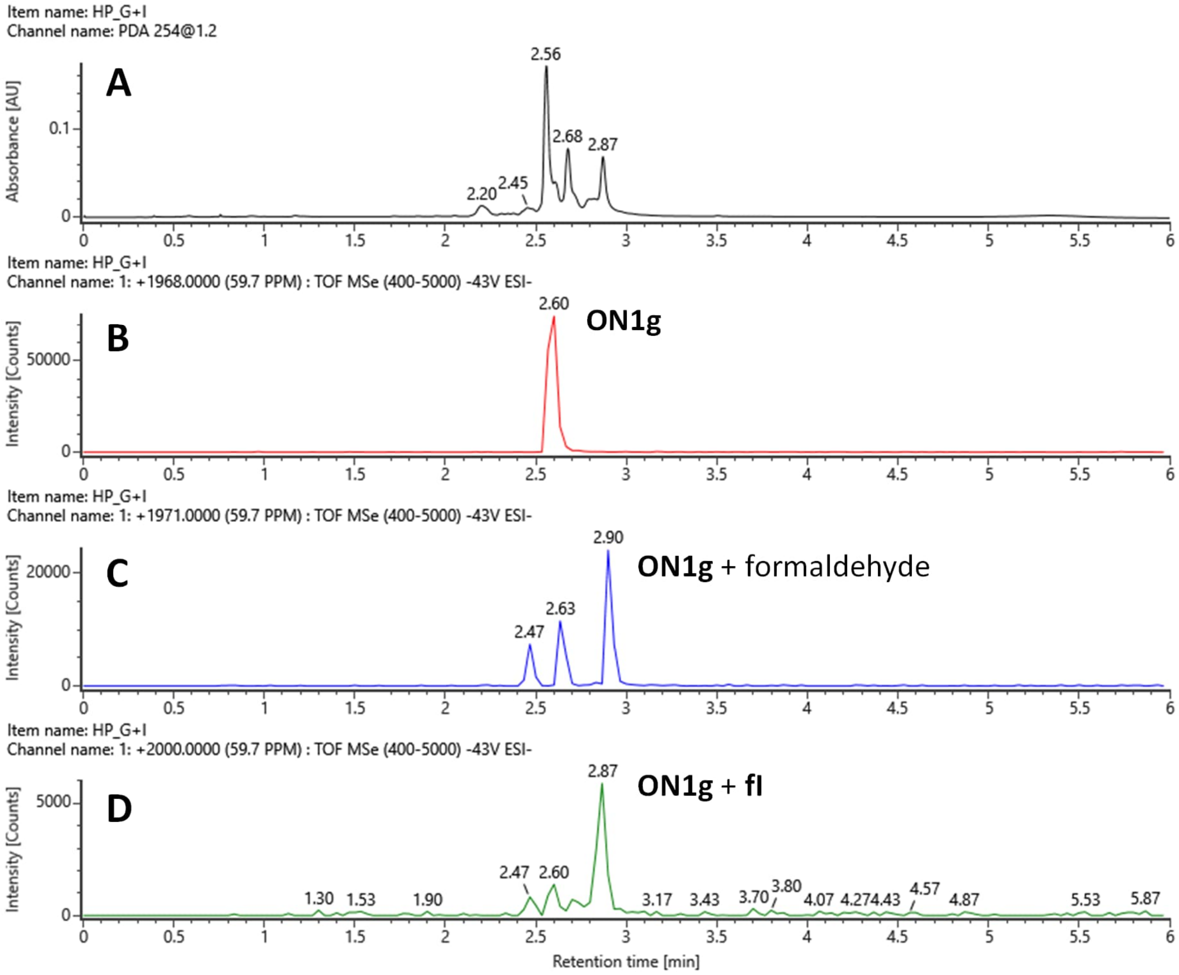

Figure S62. UV (A) and extracted ion (B—D) UPLC traces of a 50 µM mixture of hairpin oligonucleotide ON1g and fI; ACQUITY Premier OST column (50 × 2.1 mm, 1.7 µm); flow rate = 0.4 mL min-1; linear gradient (5—25% over 4 min) of MeOH in an aqueous solution of hexafluoroisopropanol (40 mM) and triethylamine (7 mM); λ = 254 nm; T = 60 °C.

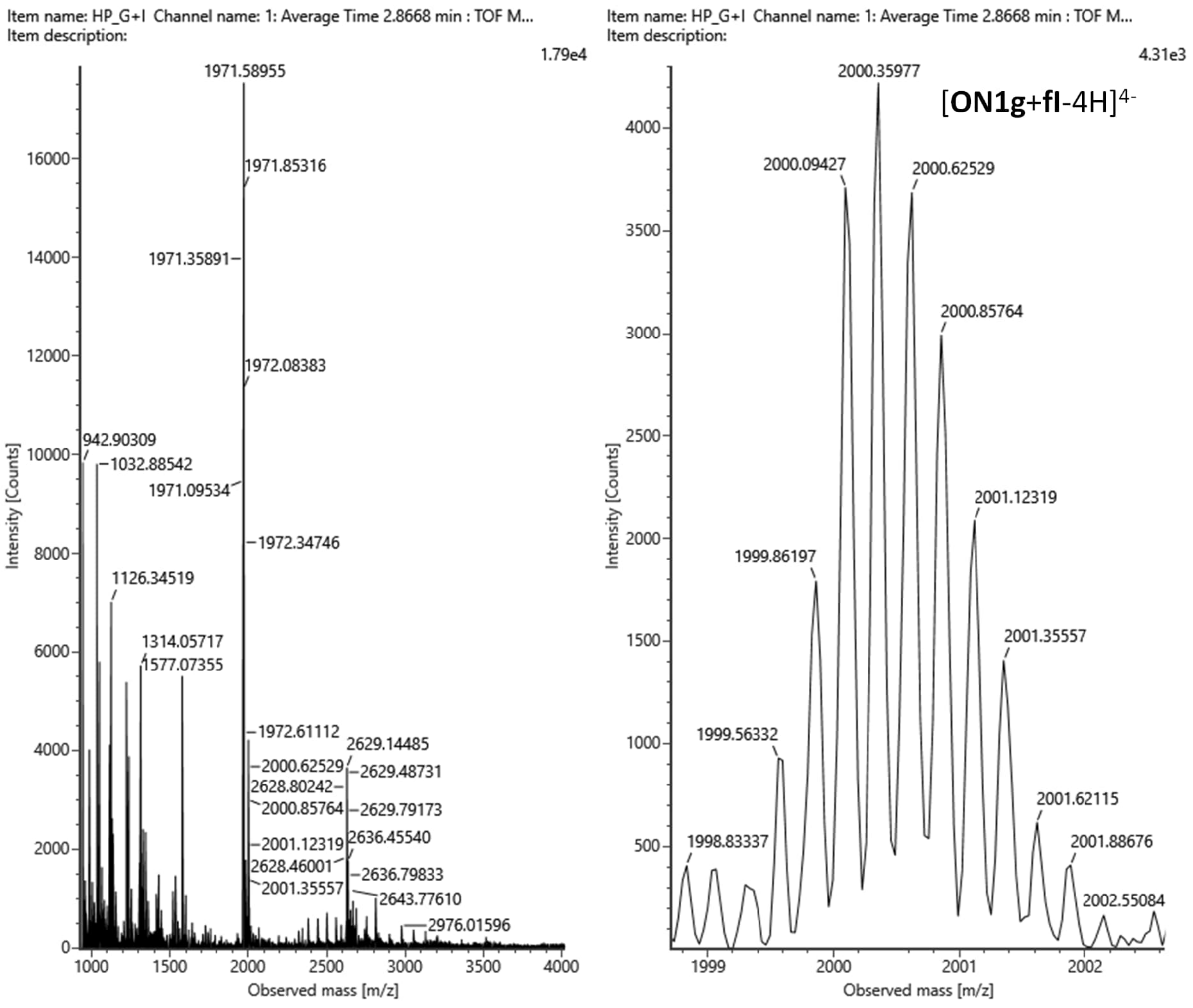

Figure S63. Mass spectrum of the covalent conjugate of hairpin oligonucleotide ON1g and **fI**.

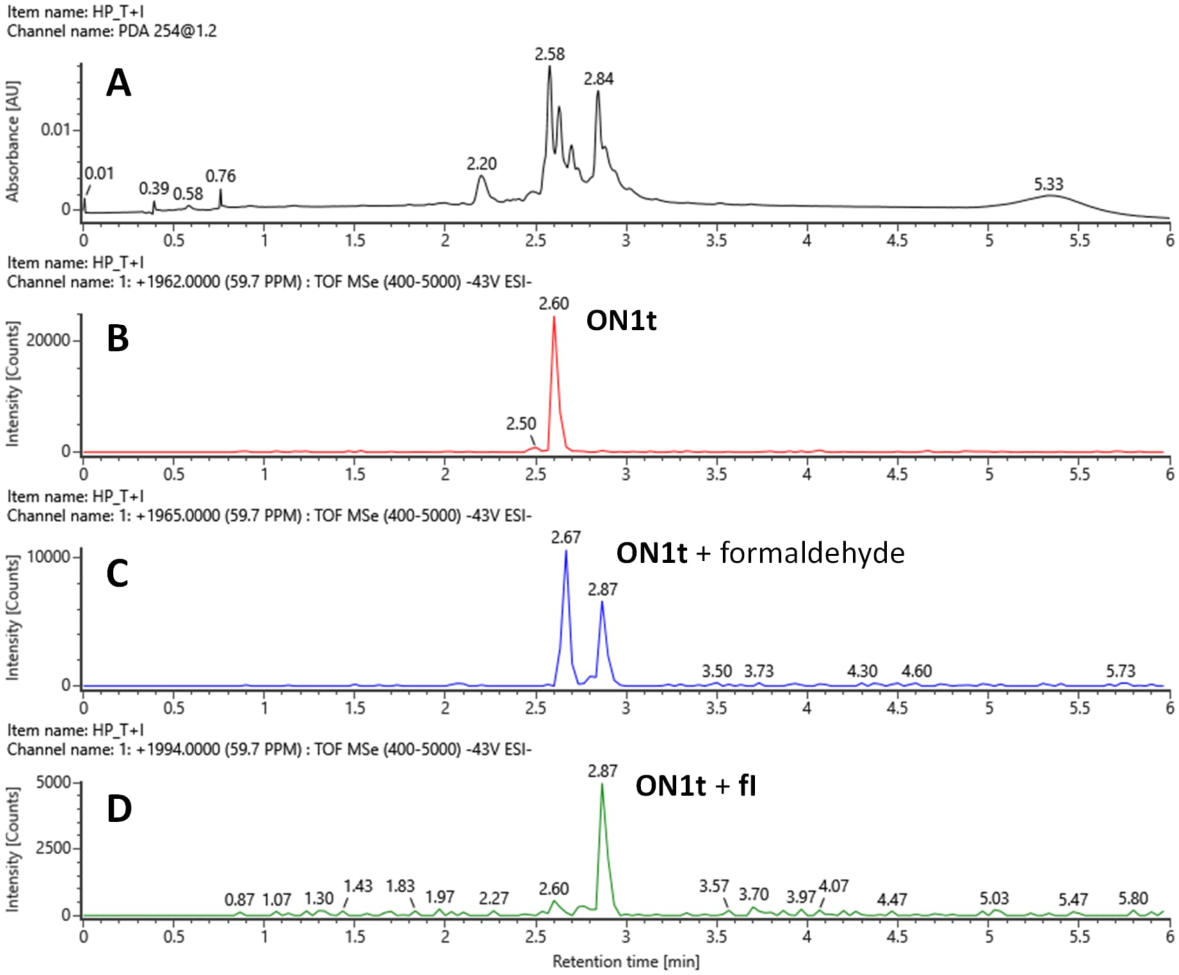

Figure S64. UV (A) and extracted ion (B—D) UPLC traces of a 50 µM mixture of hairpin oligonucleotide ON1t and fI; ACQUITY Premier OST column (50 × 2.1 mm, 1.7 µm); flow rate = 0.4 mL min-1; linear gradient (5—25% over 4 min) of MeOH in an aqueous solution of hexafluoroisopropanol (40 mM) and triethylamine (7 mM); λ = 254 nm; T = 60 °C.

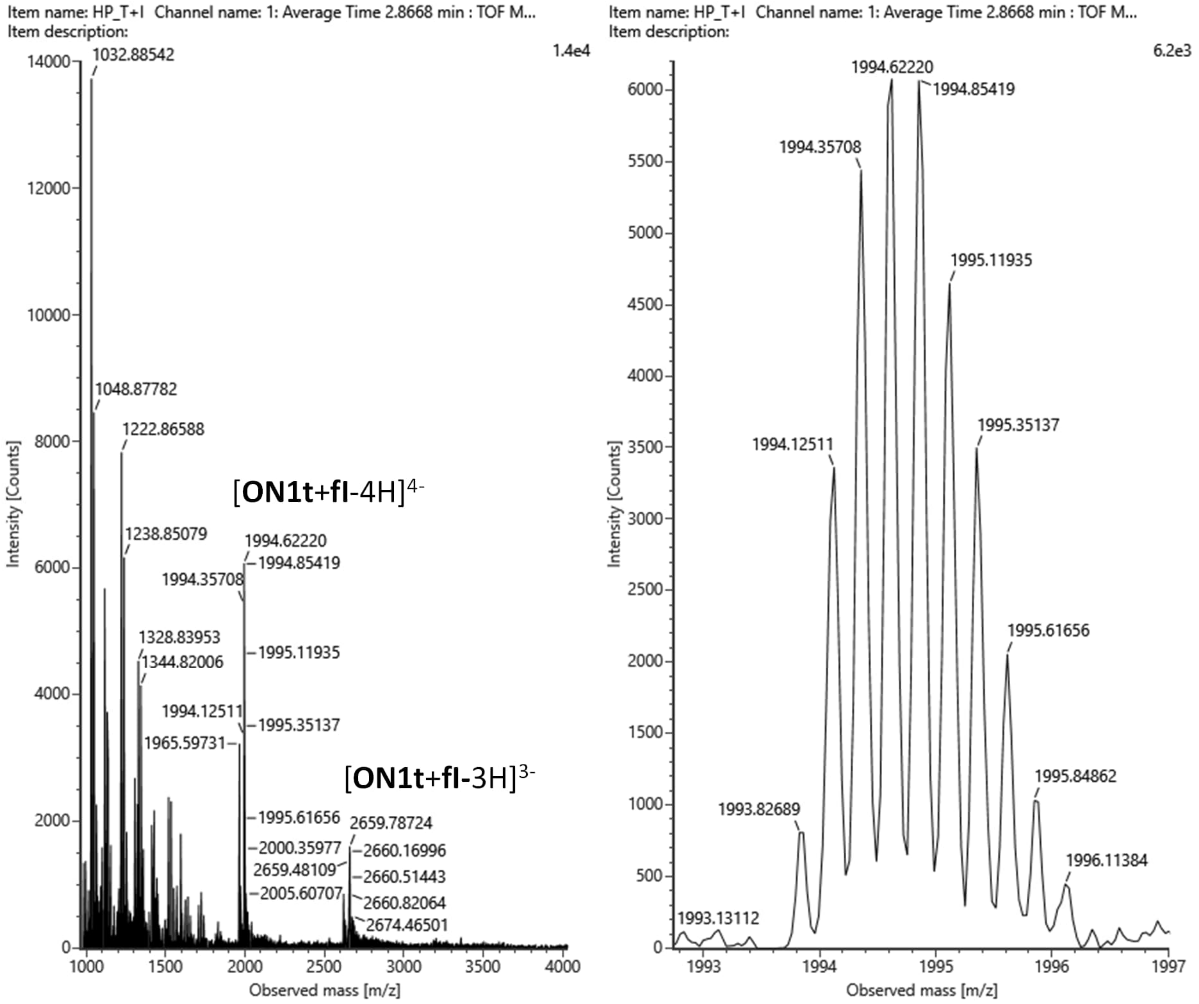

Figure S65. Mass spectrum of the covalent conjugate of hairpin oligonucleotide ON1t and **fI**.

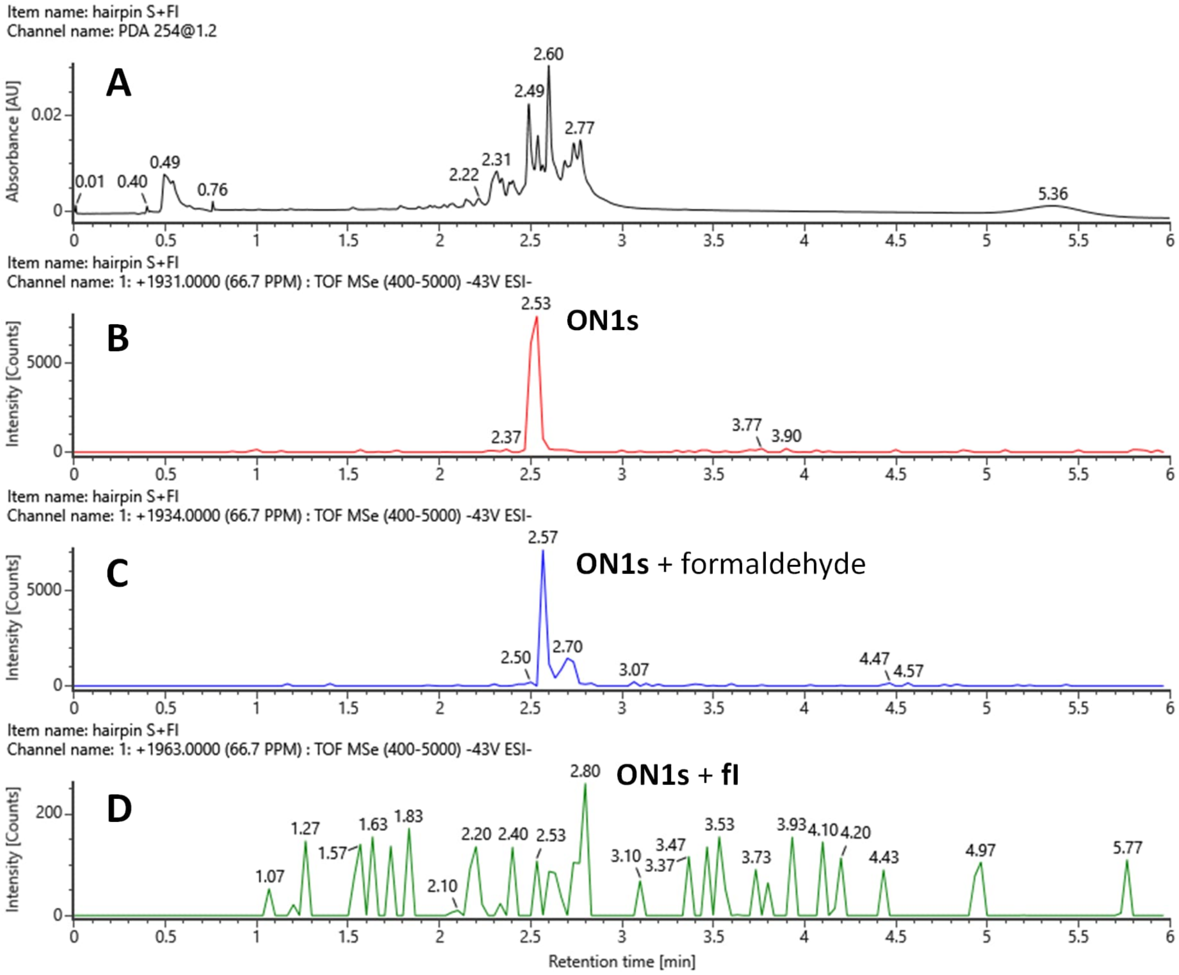

Figure S66. UV (A) and extracted ion (B—D) UPLC traces of a 50 µM mixture of hairpin oligonucleotide ON1s and fI; ACQUITY Premier OST column (50 × 2.1 mm, 1.7 µm); flow rate = 0.4 mL min-1; linear gradient (5—25% over 4 min) of MeOH in an aqueous solution of hexafluoroisopropanol (40 mM) and triethylamine (7 mM); λ = 254 nm; T = 60 °C.

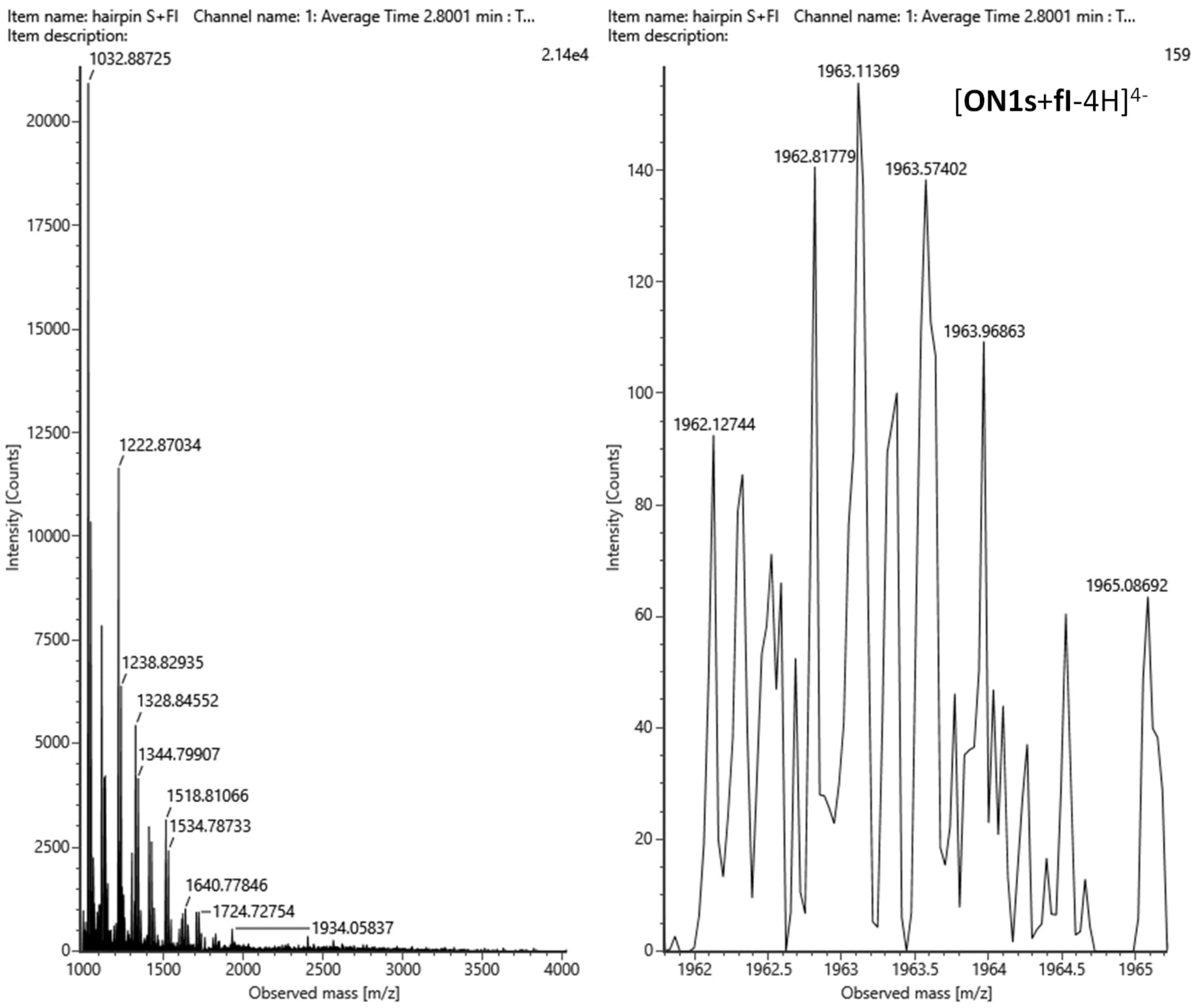

Figure S67. Mass spectrum of the covalent conjugate of hairpin oligonucleotide ON1s and fI.

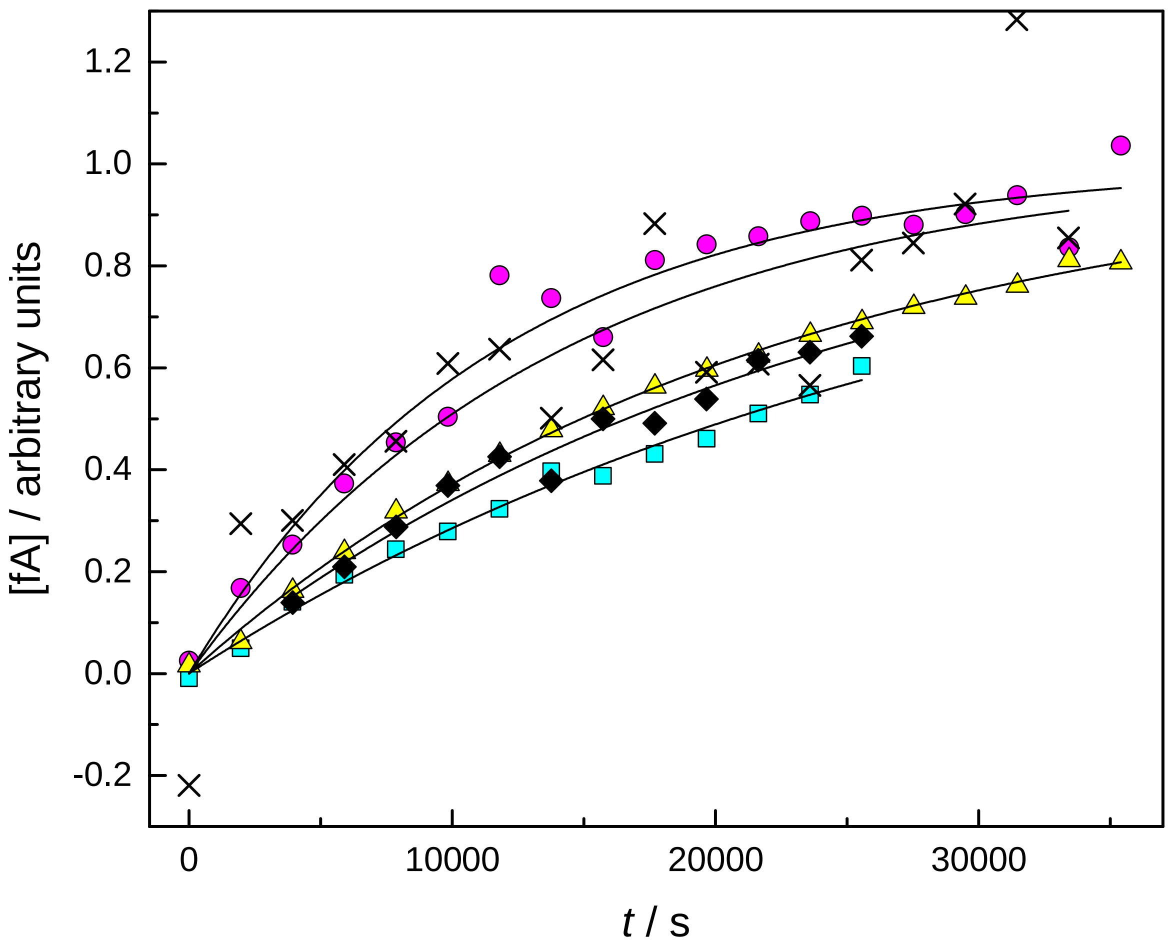

Figure S68. Relative peak area of free fA in a 1.0 µM equimolar mixture of fA and ON1a (cyan squares), ON1c (magenta circles), ON1g (yellow triangles), ON1t (black diamonds) and ON1s (crosses) as a function of time; *T* = 23 °C; pH = 5.5 (100 mM triethylammonium acetate buffer).

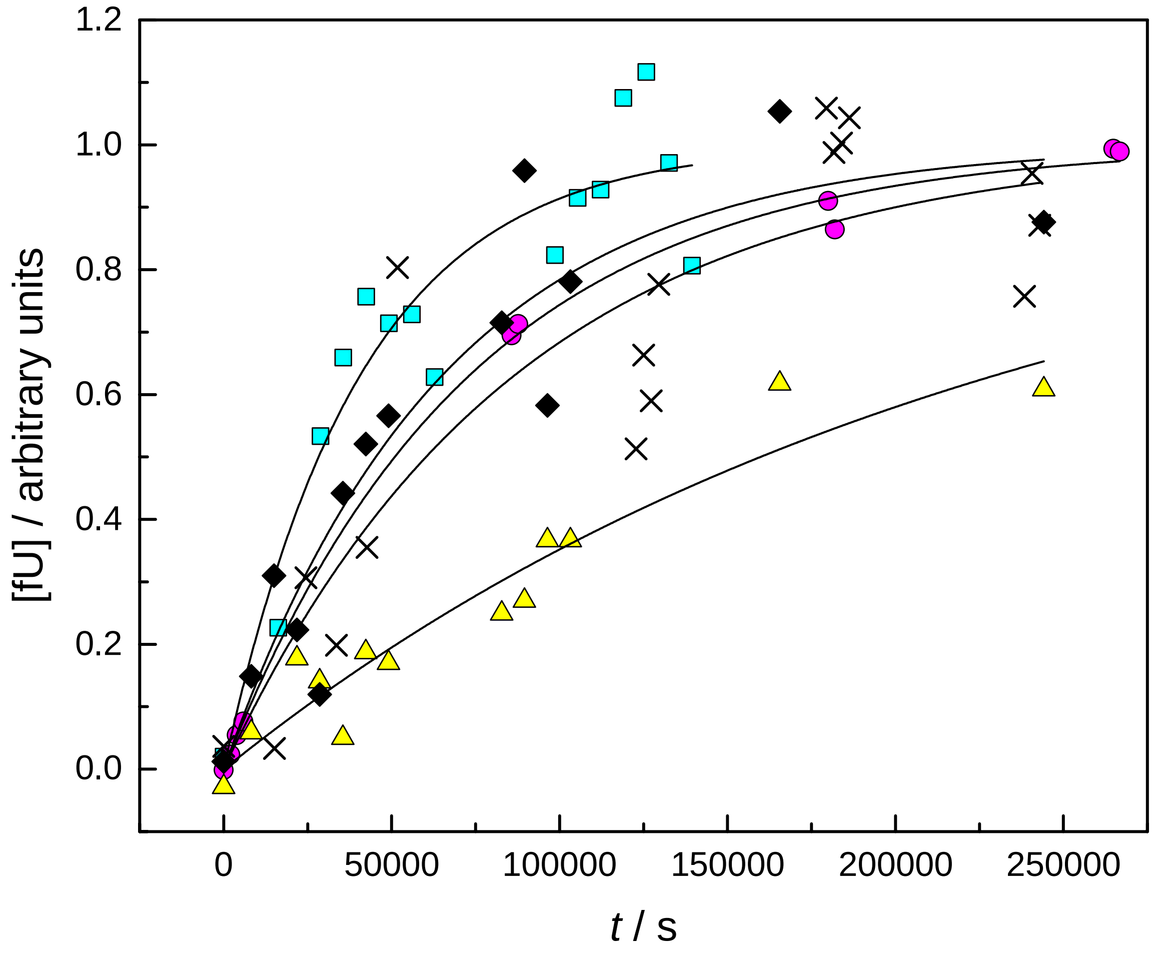

Figure S69. Relative peak area of free fU in a 1.0 µM equimolar mixture of fU and ON1a (cyan squares), ON1c (magenta circles), ON1g (yellow triangles), ON1t (black diamonds) and ON1s (crosses) as a function of time; *T* = 23 °C; pH = 5.5 (100 mM triethylammonium acetate buffer).

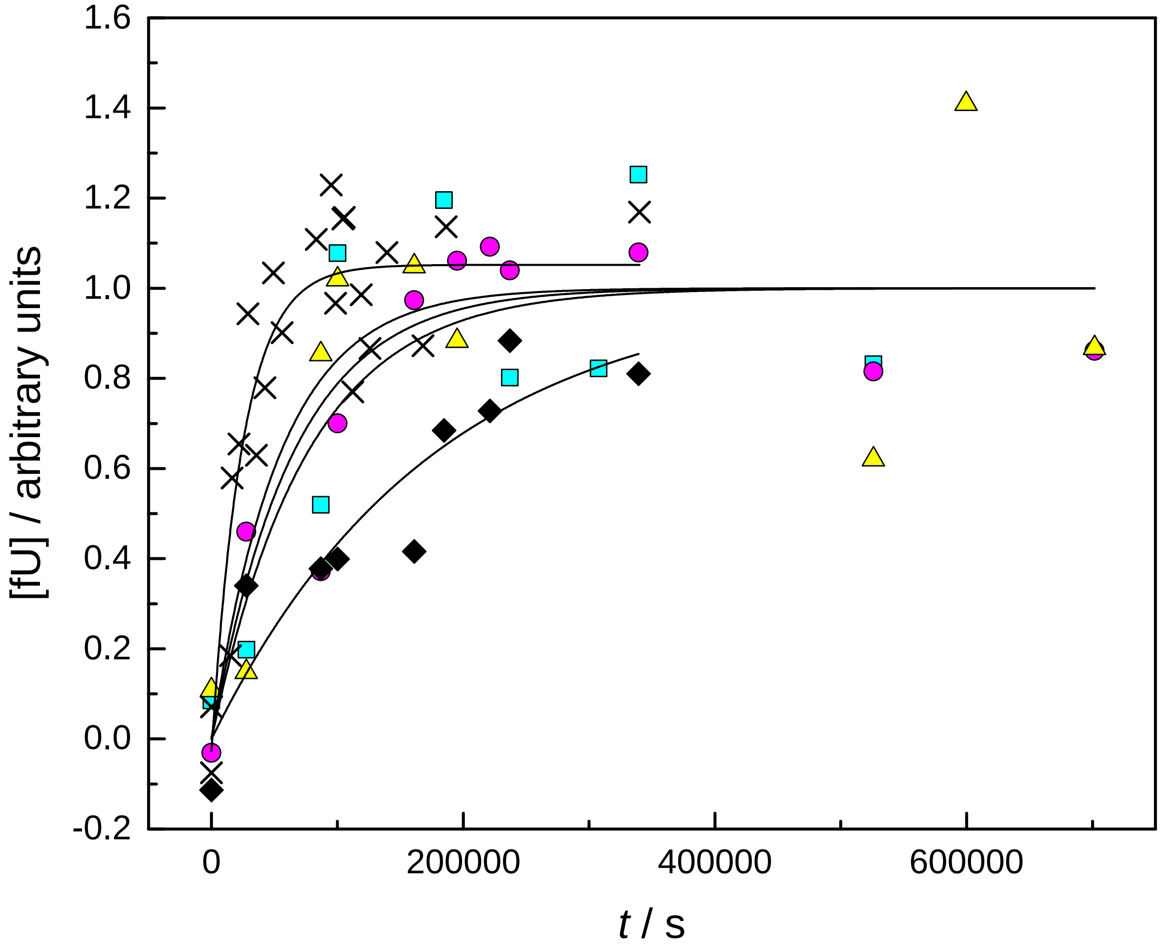

Figure S70. Relative peak area of free fI in a 1.0 µM equimolar mixture of fI and ON1a (cyan squares), ON1c (magenta circles), ON1g (yellow triangles), ON1t (black diamonds) and ON1s (crosses) as a function of time; *T* = 23 °C; pH = 5.5 (100 mM triethylammonium acetate buffer).

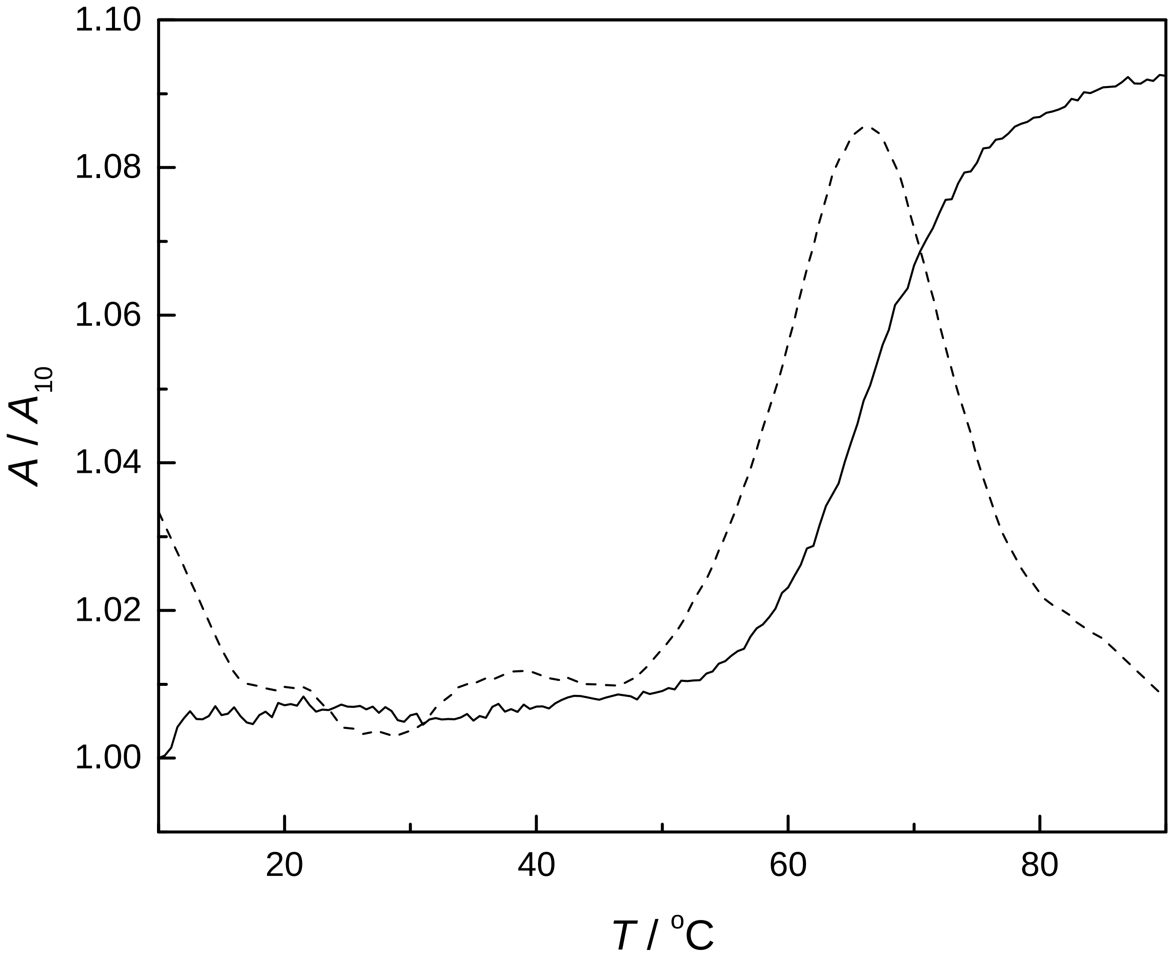

Figure S71. UV melting curve (solid line) and its first derivative (dashed line) of the covalent conjugate of hairpin oligonucleotide ON1a and fA; pH = 7.4 (20 mM cacodylate buffer); [ON1a] = [fA] = 1.0 µM; *I*($NaClO_4$) = 0.10 M.

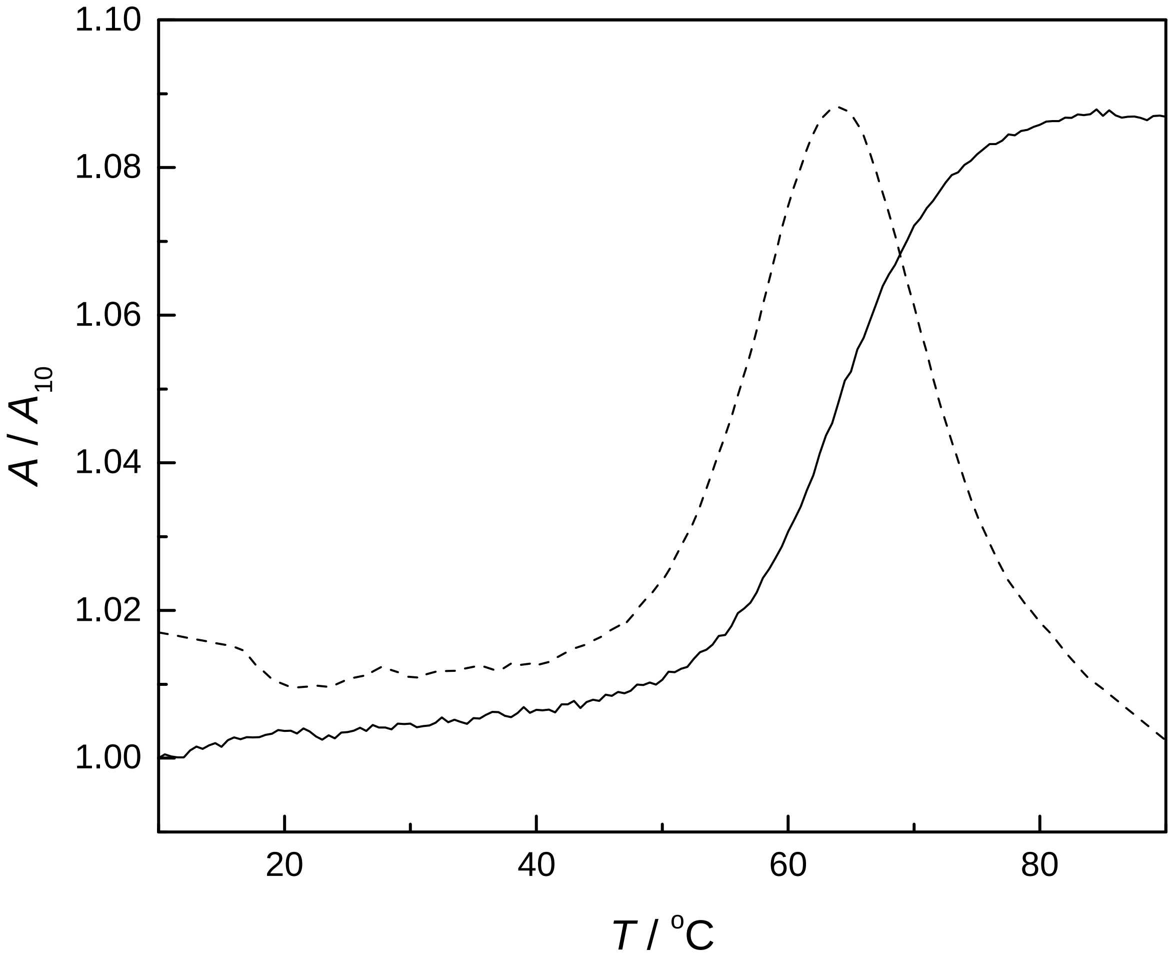

Figure S72. UV melting curve (solid line) and its first derivative (dashed line) of the covalent conjugate of hairpin oligonucleotide ON1c and fA; pH = 7.4 (20 mM cacodylate buffer); [ON1c] = [fA] = 1.0 µM; *I*($NaClO_4$) = 0.10 M.

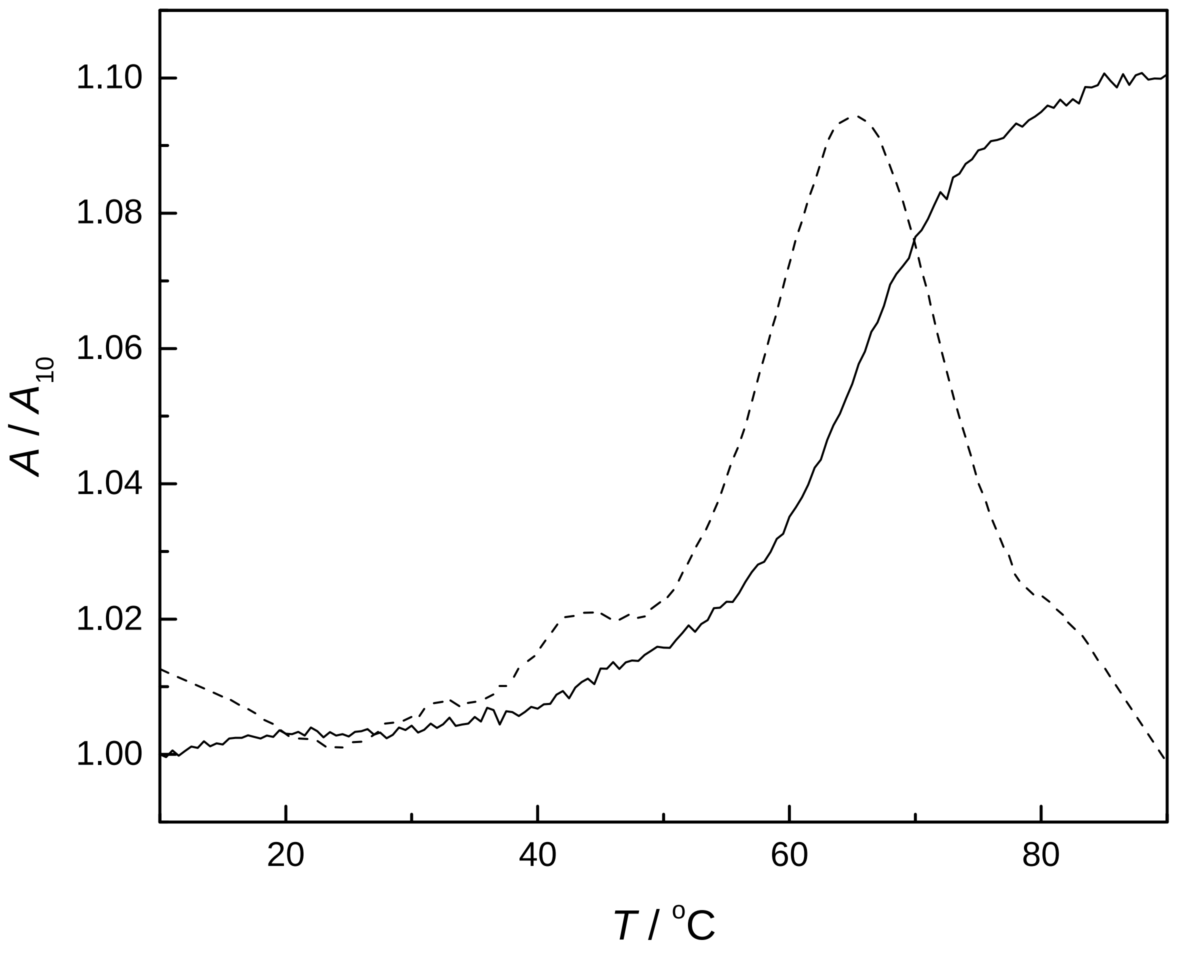

Figure S73. UV melting curve (solid line) and its first derivative (dashed line) of the covalent conjugate of hairpin oligonucleotide ON1g and fA; pH = 7.4 (20 mM cacodylate buffer); [ON1g] = [fA] = 1.0 µM; *I*($NaClO_4$) = 0.10 M.

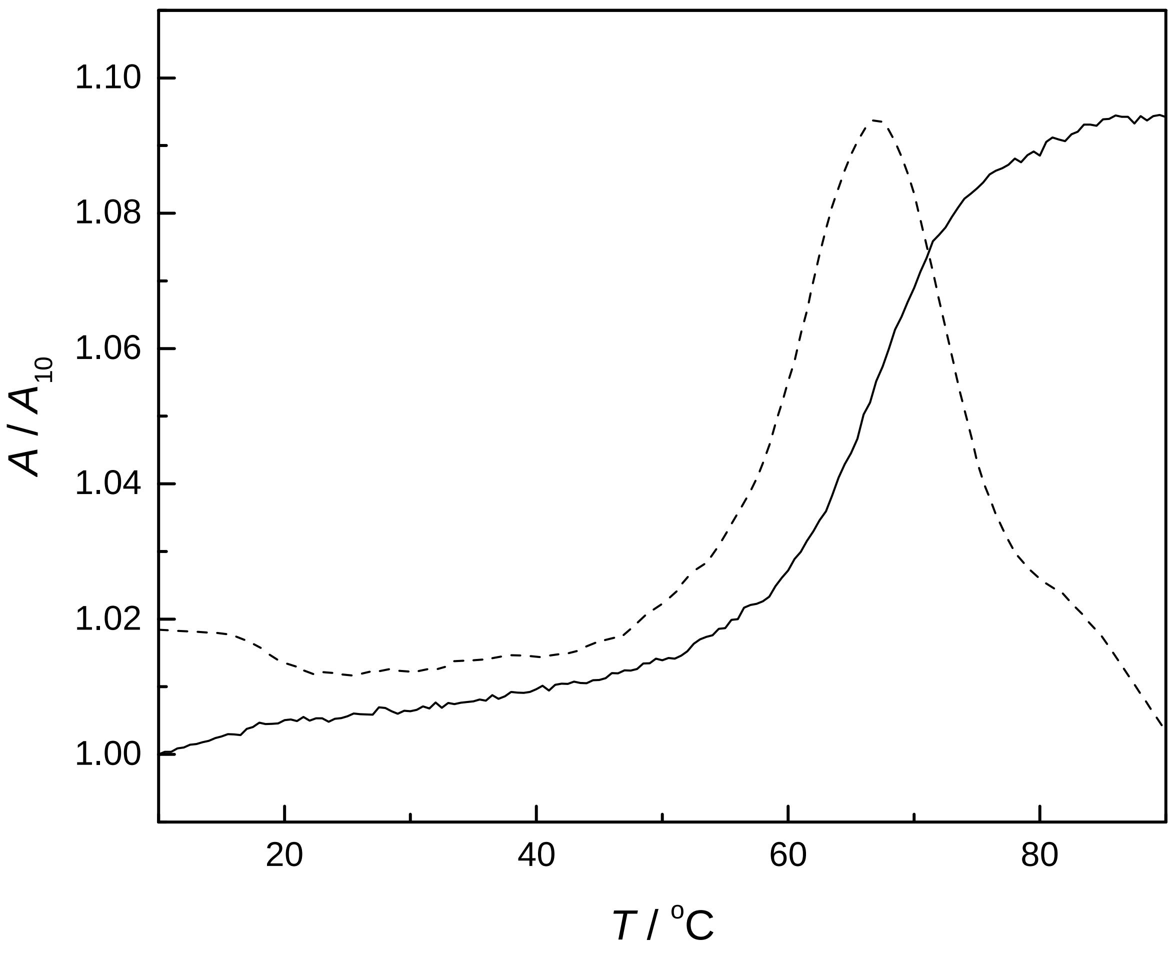

Figure S74. UV melting curve (solid line) and its first derivative (dashed line) of the covalent conjugate of hairpin oligonucleotide ON1t and fA; pH = 7.4 (20 mM cacodylate buffer); [ON1t] = [fA] = 1.0 µM; $I(NaClO_4)$ = 0.10 M.

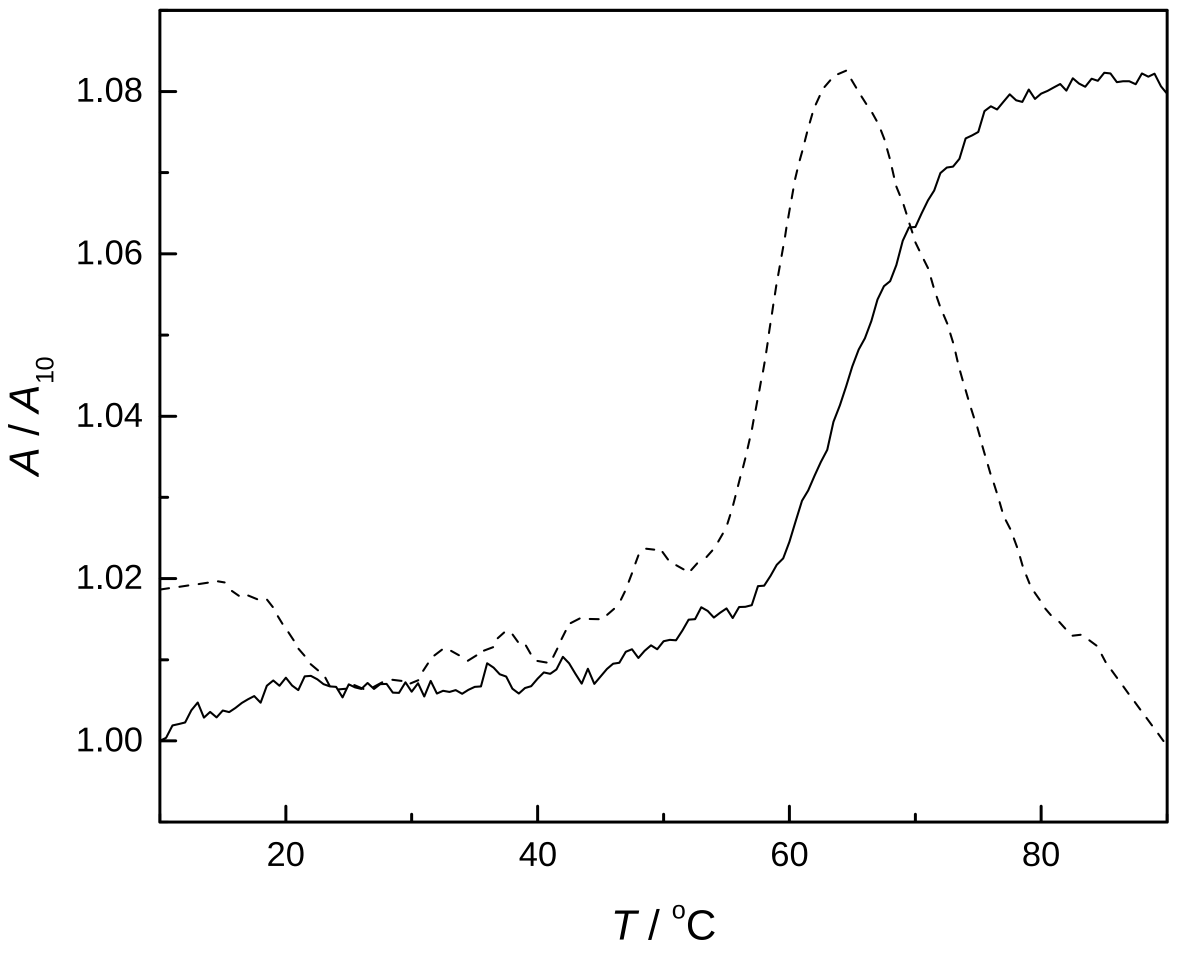

Figure S75. UV melting curve (solid line) and its first derivative (dashed line) of the covalent conjugate of hairpin oligonucleotide ON1s and fA; pH = 7.4 (20 mM cacodylate buffer); [ON1s] = [fA] = 1.0 µM; *I*($NaClO_4$) = 0.10 M.

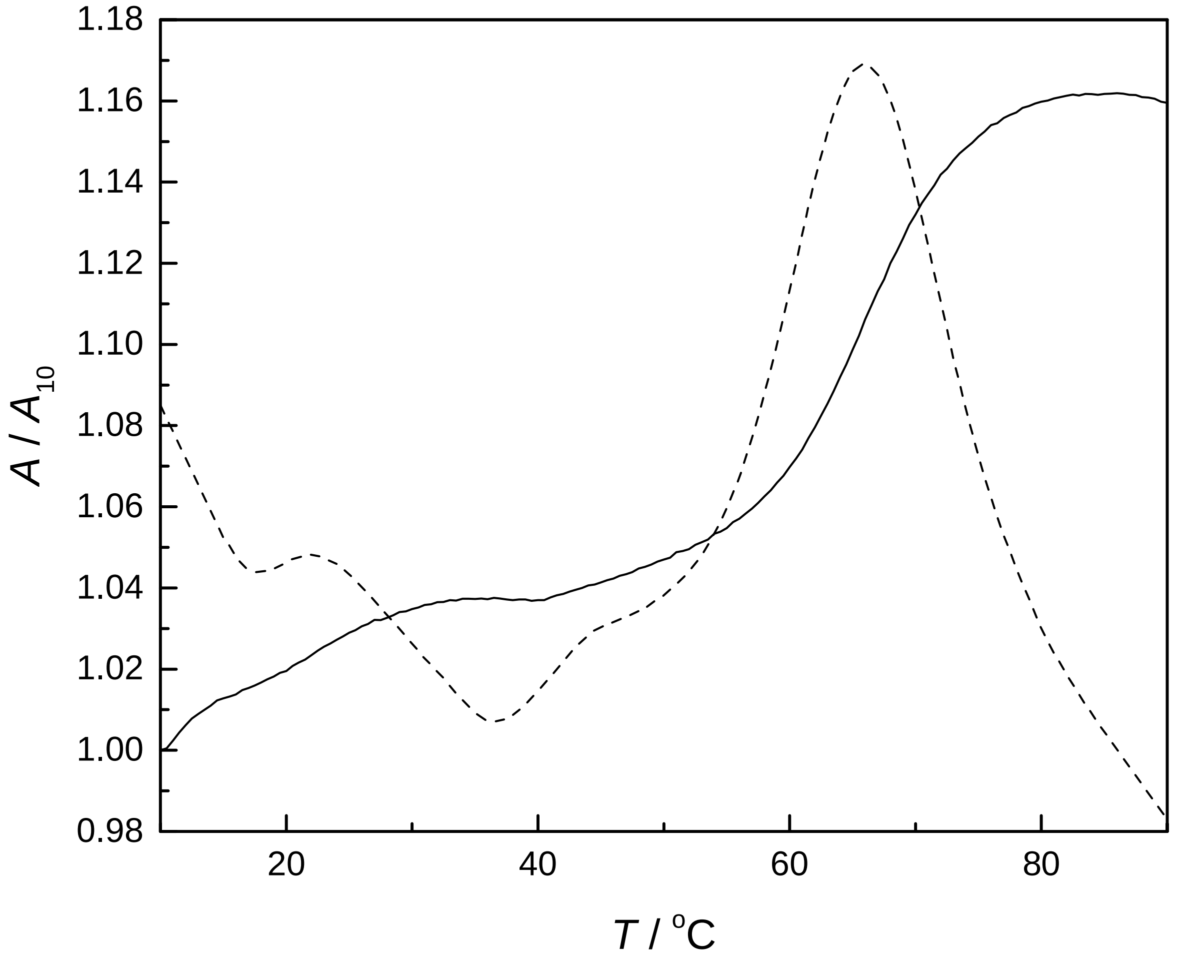

Figure S76. UV melting curve (solid line) and its first derivative (dashed line) of hairpin oligonucleotide ON1a; pH = 7.4 (20 mM cacodylate buffer); [ON1a] = 1.0 µM; *I*($NaClO_4$) = 0.10 M.

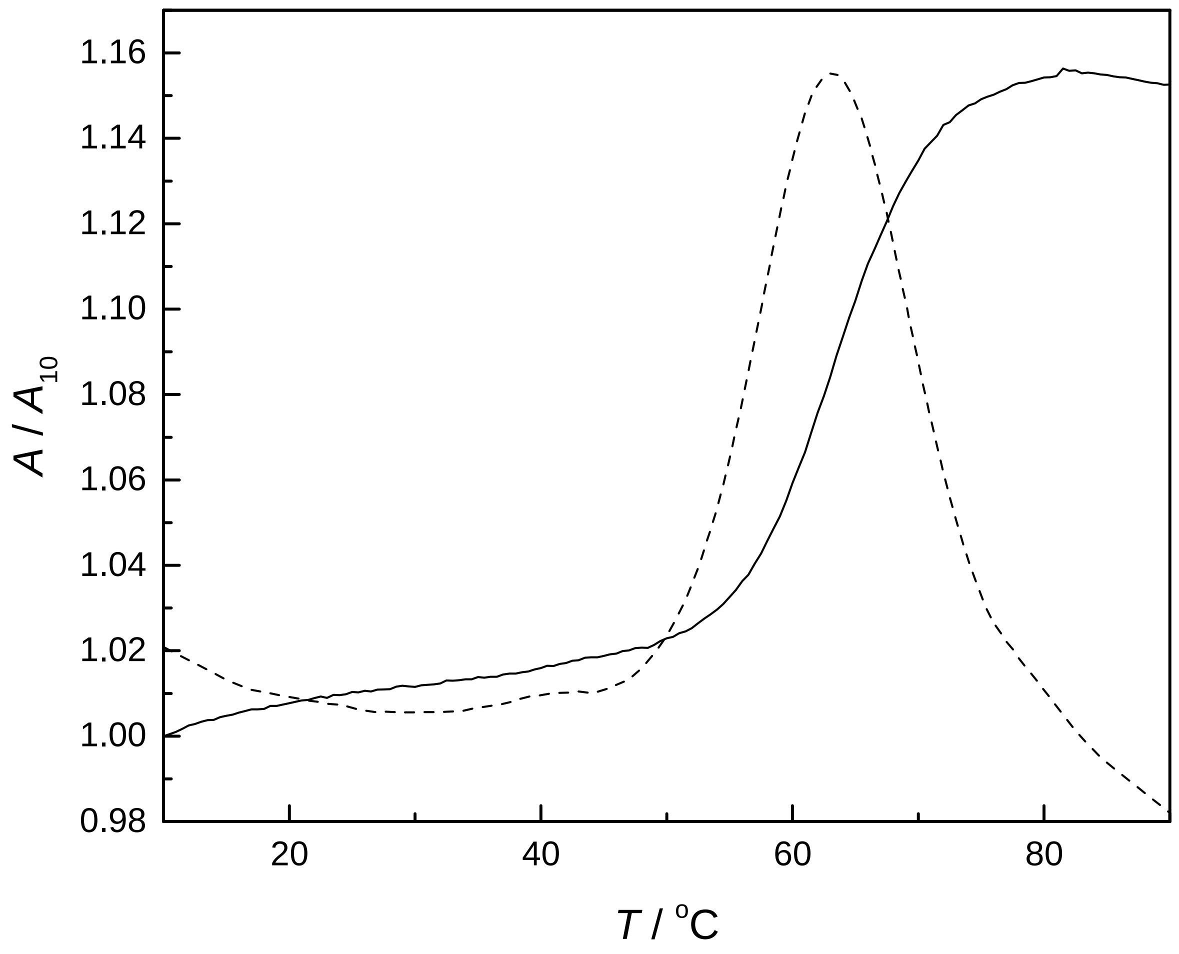

Figure S77. UV melting curve (solid line) and its first derivative (dashed line) of hairpin oligonucleotide ON1c; pH = 7.4 (20 mM cacodylate buffer); [ON1c] = 1.0 μM; $I(NaClO_4)$ = 0.10 M.

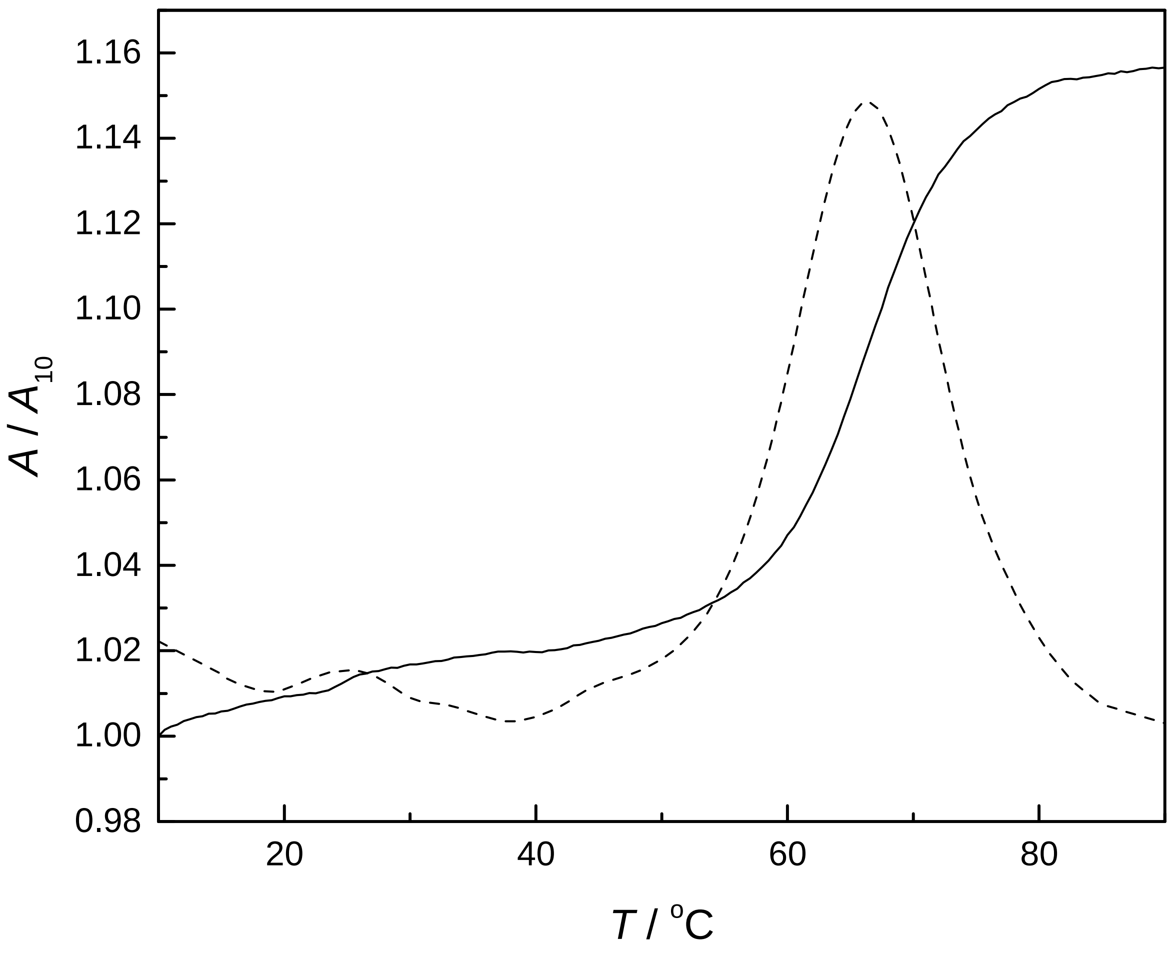

Figure S78. UV melting curve (solid line) and its first derivative (dashed line) of hairpin oligonucleotide ON1g; pH = 7.4 (20 mM cacodylate buffer); [ON1g] = 1.0 μM; *I*($NaClO_4$) = 0.10 M.

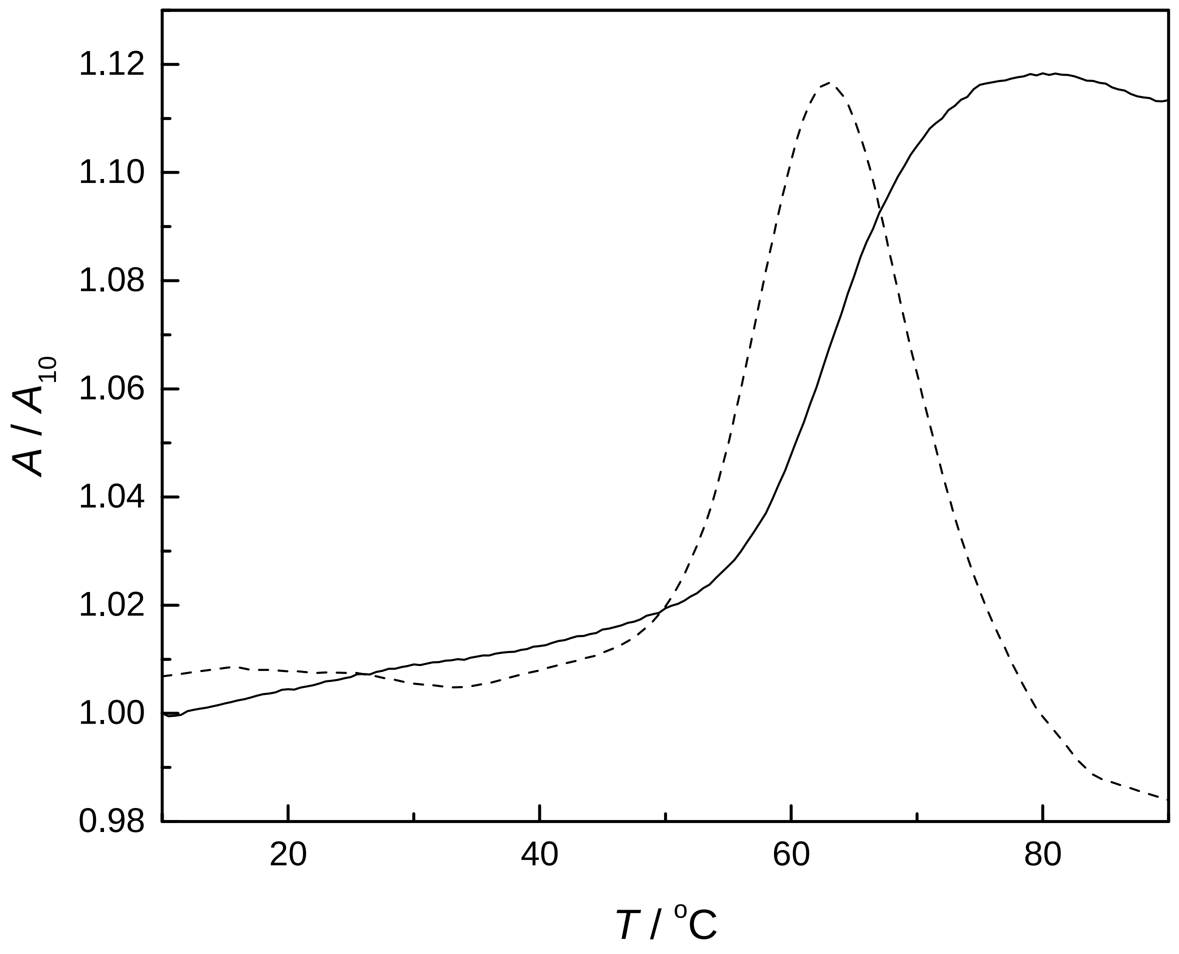

Figure S79. UV melting curve (solid line) and its first derivative (dashed line) of hairpin oligonucleotide ON1t; pH = 7.4 (20 mM cacodylate buffer); [ON1t] = 1.0 µM; *I*($NaClO_4$) = 0.10 M.

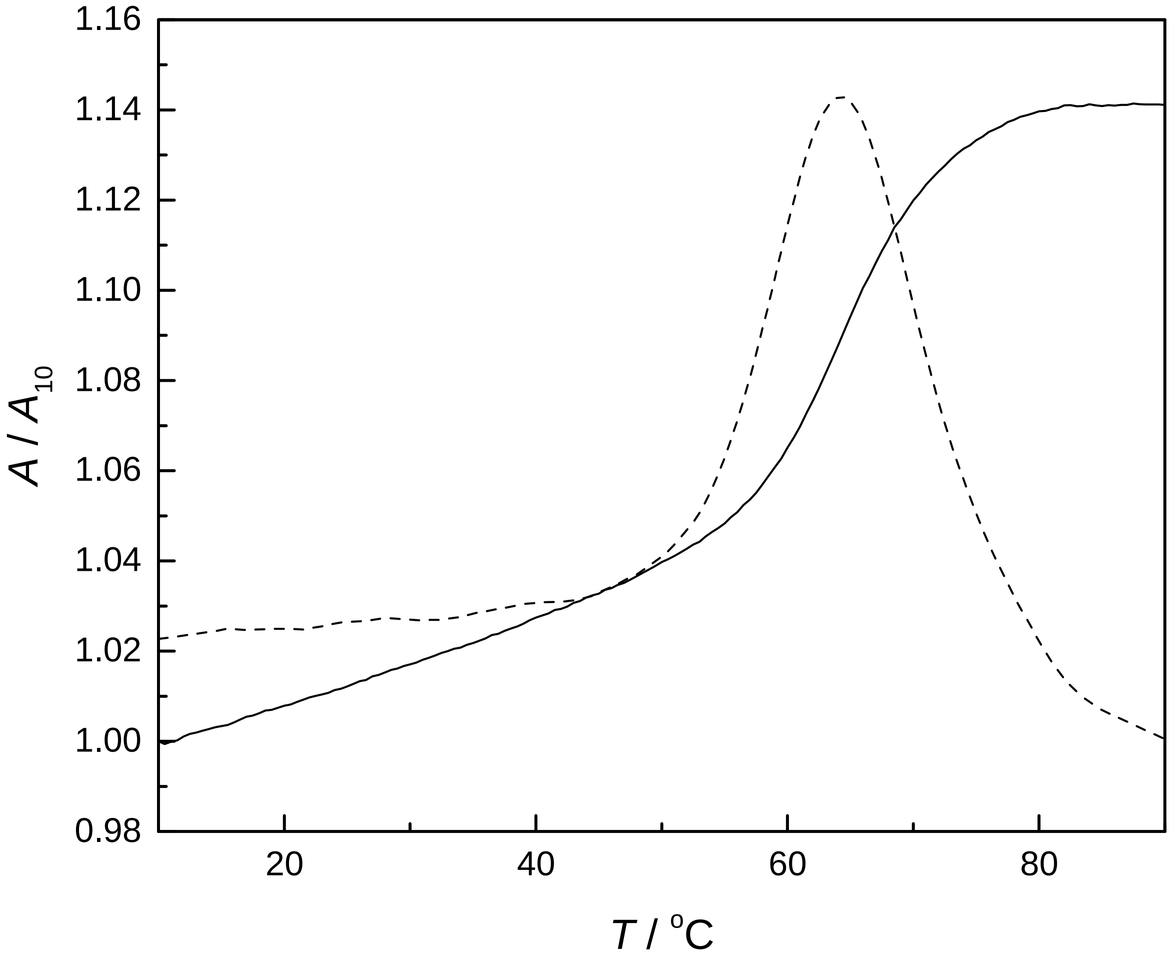

Figure S80. UV melting curve (solid line) and its first derivative (dashed line) of hairpin oligonucleotide ON1s; pH = 7.4 (20 mM cacodylate buffer); [ON1s] = 1.0 µM; $I(NaClO_4)$ = 0.10 M.
